# Trophic modulation of endophytes by rhizosphere protists

**DOI:** 10.1093/ismejo/wrae235

**Published:** 2024-11-20

**Authors:** Rasit Asiloglu, Seda Ozer Bodur, Solomon Oloruntoba Samuel, Murat Aycan, Jun Murase, Naoki Harada

**Affiliations:** Institute of Science and Technology, Niigata University, Nishi-ku, Niigata 950-2181, Japan; Graduate School of Science and Technology, Niigata University, Nishi-ku, Niigata 950-2181, Japan; Department of Plant and Microbial Biology, North Carolina State University, Raleigh, NC 27695, United States; Graduate School of Science and Technology, Niigata University, Nishi-ku, Niigata 950-2181, Japan; Graduate School of Bioagricultural Sciences, Chikusa-ku, Nagoya University, Nagoya 464-8601, Japan; Institute of Science and Technology, Niigata University, Nishi-ku, Niigata 950-2181, Japan

**Keywords:** protists, endophytic bacteria, root community, rhizosphere soil, trophic interaction, plant microbiome

## Abstract

The plant-microbe interactions, which is crucial for plant health and productivity, mainly occur in rhizosphere: a narrow zone of soil surrounding roots of living plants. The rhizosphere hosts one of the most intense habitats for microbial prey–predator interactions, especially between predatory protists and bacteria. Here, based on two key facts, microbial predators modulate rhizobacterial community composition, and the rhizobacterial community is the primary source of root microbiome, endophytes; we hypothesized that predation upon rhizobacteria would modulate the community composition of endophytic bacteria. The effects of three taxonomically distinct axenic protist species (*Acanthamoeba castellanii*, *Vermamoeba vermiformis*, and *Heteromita globosa*) were tested in this study. To examine the robustness of the hypotheses, the experiments were conducted in three soil types characterized by distinct bacterial communities and physicochemical properties. The bacterial community compositions were analyzed with high throughput sequencing. Bacterial gene abundances were estimated with a real-time-PCR method. The results showed that protists modulated endophytic communities, which originated in the rhizosphere soil. The modulation of endophytic communities by protists showed chaotic patterns rather than a deterministic effect under different soil types. The observed chaotic dynamics were further confirmed with an additional experiment, in which chaos was triggered by changes in the dilution rates of soil nutrients. Furthermore, the presence of predators enhanced the root colonization of endophytes. Our findings identify a key mechanism for the modulation of root endophytes and enhance understanding of underground plant-microbe interactions, which can lead to open new avenues for modulating the root microbiome to enhance crop production.

## Introduction

Phagotrophy, the ability of a cell to ingest alive or dead organic material as food, is one of the most impactful evolutionary events in the history of life, without which there would be no animals and no plants. Phagotrophy that distinguishes eukaryotes from all other forms of life has been spread across many lineages of protists enabling them to hunt, kill, and consume other microbes, especially bacteria, across various ecosystems [1]. The rhizosphere, a narrow zone of soil surrounding the roots of living plants, hosts one of the most intense habitats for microbial prey–predator interactions [2]. Plant roots deposit a wide range of organic and inorganic compounds [3] making the rhizosphere a nutrient-rich habitat for bacteria and thus increasing the bacterial populations in the rhizosphere [3, 4]. As bacteria are the primary food source of predatory protists, the bacteria-enriched rhizosphere attracts protists, creating everlasting prey–predator dynamics [2]. The simple phagotrophic behavior has complex outcomes for prey communities. Protists selectively feed on bacteria and the protist-preyed bacterial population markedly decreases, whereas the bacterial groups that can avoid predation take advantage of the trophic interactions through mainly nutrients released from the preyed bacterial cells (the nutrient turnover) [2, 5]. Protists show prey-selection patterns that are mainly species-specific; therefore, predator’s traits such as feeding mode and cell size play important roles for the outcome of the protist predation on bacterial community composition and activities [1, 2, 5]. Despite the selective feeding by protists being deterministic, chaos—complex systems that exhibit unpredictability with sensitive dependence on initial conditions [6]—occurs in microbial communities, host–microbe interactions, bacterial stress response, and microbial trophic interactions [7–10]. For instance, chaos has been demonstrated in a defined predator–prey system consisting of a protist and two bacterial prey species, where the changes in the dilution rates of the chemostat triggered chaos revealing different dynamics in trophic interaction depending on the initial nutrient levels [8]. Although less is known about whether protists modulate bacterial communities chaotically or deterministically, protist predation is considered one of the most crucial and complex factors controlling rhizobacterial communities [2, 11].

The rhizobacterial communities not only support plant growth but also play an essential role as the primary source of root endophytes [12, 13], a vital part of the plant microbiome that promotes plant productivity and health by enhancing immunity, nutrient acquisition, and tolerance to environmental stresses [13]. A shift in the source, the rhizobacterial communities, by environmental factors inevitability alters endophytic bacterial communities [12, 14, 15]. However, despite the importance of microbial trophic interaction in the rhizosphere soil (RS) [2], its effect on endophytic communities has never been studied. Here, based on two key facts, protists alter rhizobacterial community composition [2], and the rhizobacterial community is the primary source of root endophytes [12, 13], we hypothesized that protist predation upon rhizobacteria would modulate the community composition of endophytic bacteria associated with the plant roots. As the plant roots are colonized by endophytes—predatory protists are not known to be endophytic—, we assumed that plant roots can provide a protective shelter for rhizobacterial taxa with root colonization traits. Therefore, our 2^nd^ hypothesis is that the presence of protists would propel rhizobacteria with root colonization competence to escape into the roots resulting in enhanced root colonization. As predator–prey interactions show species-specific patterns [2, 5], we anticipated that both of our hypotheses could be influenced by the protist species. Therefore, the effects of three taxonomically distinct axenic protist species (*Acanthamoeba castellanii* [Ac], *Vermamoeba vermiformis* [Vv], and *Heteromita globosa* [Hg]) were tested in this study. To examine the robustness of the hypotheses, the experiments were conducted in three soil types characterized by distinct bacterial communities and physicochemical properties. As the previous studies in the rice rhizosphere already concluded the premises of our first hypothesis, protists alter the rhizobacterial communities in the rhizosphere of paddy fields [11] and the rhizobacteria are the major source of rice root endophytes [16], here, we studied with rice plants (*Oryza sativa* L. Nipponbare), one of the most essential crops feeding more than half of the world’s population.

In this study, we first obtained an indigenous protist-free bacterial community from each of the three soil types by a filtering method. Then each sterilized paddy field soil was re-colonized with its native bacterial community with and without the axenic protist isolates. One sterile rice seedling was transplanted into each microcosm. The vegetative stage is a critical period for rice root-associated microbial communities [16]; therefore, destructive sampling was performed at the 3rd and 6th week of rice growth. The RS and root endophytic (RE) bacterial community compositions were analyzed with a high throughput sequencing method. Bacterial gene abundances were estimated with a real-time PCR method.

## Materials and methods

### Soil samples and sterile rice seedlings

We collected paddy field soils from three different locations in Japan that demonstrate distinct bacterial communities and physicochemical properties of each other [17, 18]. Soil samples were taken from the plow layer (0–10 cm) of three Japanese paddy fields. Soil 1 (Stagnosol) was sampled on 18th April 2021 from Chita, Aichi (N34.56.47, E136.53.24); Soil 2 (Fluvisol) was sampled on 25th March 2021 from Shindori Station in the Field Center for Sustainable Agriculture and Forestry, Niigata University, Niigata (N37.85.69, E138.96.22); and Soil 3 (Andosol) was sampled on 23rd April 2021 from Hata, Matsumoto city, Nagano (N36.20.08, E137.87.03). We studied with a model rice plant, *O. sativa* L. Nipponbare. The sterile rice seedlings were prepared as described previously [19]. The details of soil sampling and sterile rice seedlings are provided in the supporting materials and methods.

### Protist-free bacterial community and the axenic protist isolates

The protist-free indigenous bacterial community was obtained by a filtration method (1.2 μm pore size mixed cellulose ester membrane filters [Advantec, Tokyo, Japan]) from the collected paddy field soils as described previously [20]. The axenic cultures of *A. castellanii* (30234) and *V. vermiformis* (previously known as *Hartmannella vermiformis*) (50256) were purchased from the American Type Culture Collection (ATCC). Axenic culture of *H. globosa* (Rhizaria; Cercozoa) (~10 μm) that was previously isolated from a paddy field soil [19] was obtained as described in the supporting materials and methods.

### Experimental setup and sampling

The soil was sterilized by γ irradiation (25 kGy, ^60^Co) at the School of Engineering, Nagoya University. The microcosms were established, as described previously [19]. Briefly, the sterile centrifuge tubes (volume: 50 ml) were filled with 40 g of sterile paddy field soil. Each soil type was re-inoculated with corresponding protist-free bacterial media (~10^7^ cells per g dry soil). To obtain a stable bacterial community, the microcosms were preincubated for a week under the submerged condition at room temperature (25°C) in the dark. We had 4 treatments (CK; Control with no protist addition; Ac, *A. castellanii; Vv, V. vermiformis*; Hg, *H. globosa)* for each soil type, making in total 12 treatments. Each treatment was prepared with 10 replications. About 500 cells g^−1^ soil of each axenic protist species were added into the microcosms, whereas CK treatment received the same amount of sterile water. One sterile rice seedling (14-day-old) was transplanted into each microcosm. The microcosms were incubated under submerged conditions in a growth chamber at 25/30°C (day/night) with a day length of 16 h (250 μmol m^−2^ s^−1^). Five replications of the microcosms for each treatment were destructively sampled at 3rd and 6th week. The sampling of each microcosm was performed as described in the supporting materials and methods.

### Molecular analysis, bioinformatics, and statistics

DNA was extracted from the RS (0.5 g) using ISOIL for Bead Beating (Nippon Gene, Tokyo, Japan) and from the roots (0.15 g) using ISOPLANT (Nippon Gene, Tokyo, Japan) according to the manufacturer's instructions and then eluted in TE buffer (50 μl). The V4 region of the 16S rRNA gene was amplified from the extracted DNA using the universal primers (515F and 806R) tailed with Illumina barcoded adapters (San Diego, CA, USA) [21]. Negative control was used in all steps from the DNA extraction to the PCR amplification to make sure contamination did not occur. MiSeq sequencing (Illumina) and primary analyses of raw FASTQ data were performed as described previously [19]. Total bacterial gene abundances were detected by a quantitative real-time PCR (qPCR) as described in the supporting materials and methods.

All analyses of bioinformatics and statistics are described in the supporting materials and methods.

### Additional (2nd) experiment

As trophic modulation of RE communities under different soil types indicated the possibility of *deterministic chaos,* here an additional experiment was conducted to confirm the chaotic dynamics controlling trophic modulation of RE communities. We used calcined clay as an inert soil substitute allowing us to control nutrient levels [15, 22]. The calcined clay was washed with ddH_2_O until the runoff was clear. Then it was sterilized with an autoclave (120°C, 20 min), followed by drying at 105°C until completely dehydrated. Protist-free bacterial community, the three protist isolates, and the sterile rice seedlings were prepared as described above. Here we used the bacterial community of Soil 1 as a model community. The sterile centrifuge tubes (volume: 50 ml) were filled with calcined clay (27 g DW, 40 ml) and a defined nutrient solution: Kimura B (pH: 5.8) [15] with dilution rates of ×1, ×0.75, and ×0.5. The dilution rates were determined based on a previously designed experiment [8] and the Kimura B solution is a commonly used nutrition in rice plants [15]. The rest of the experimental set-up conducted as described in the first experiment. Briefly, we had four treatments (CK; Control with no protist addition; Ac, *A. castellanii*; *Vv*, *V. vermiformis*; Hg, *Heteromita globosa*) for each nutrient concentration, making a total of 12 treatments. Each treatment was prepared with five replications. Although constant population densities for prey–predator interaction can be obtained in 5 days for simple systems, for instance, including one predator and two prey [8], our previous experiments showed that the effect of protists on bacteria at the community level can be clearly observed in 3 weeks [19, 20]. Therefore, we conducted a one-time sampling on the 3rd week. The sampling, the afterward molecular analysis (DNA extraction, PCR amplification, Miseq sequencing, and qPCR analysis), and the bioinformatics analyses were the same as previously described. All of the raw sequence data obtained in this study have been deposited in the NCBI database under the BioProject ID PRJNA1106748.

## Results and discussion

### Protists modulate RE communities

**Figure 1:**
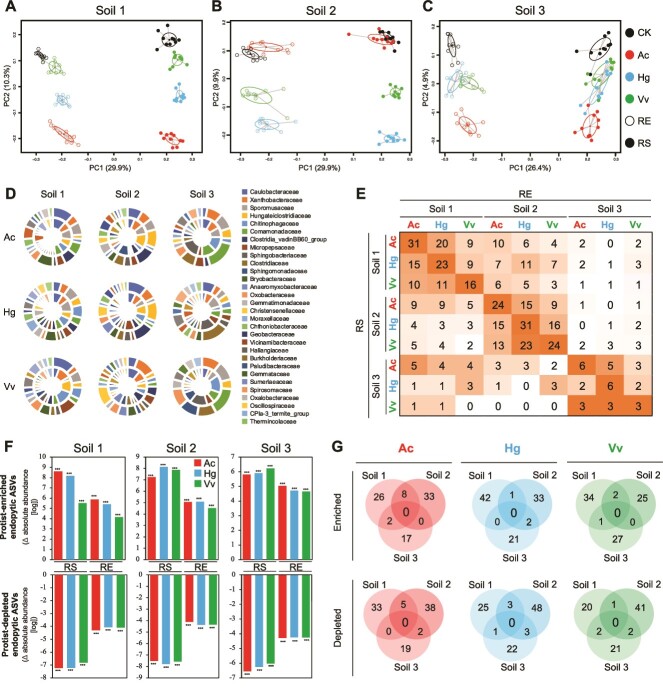
**Trophic modulation of RS and RE bacterial communities by protists based on absolute abundances of bacterial taxa.** (**A–C**) principal coordinate analysis (PCoA) based on the Bray–Curtis dissimilarities calculated from the log transformed absolute abundances of bacterial taxa for soil 1(**a**), 2(**b**), and 3(**c**) showing the effect of protists with confidence ellipses of the eigenvalues of the covariance matrix. Black, control without protists (CK); red, *Acanthamoeba castellanii* (Ac); blue, *Heteromita globosa* (Hg); green, *Vermamoeba vermiformis* (Vv); filled circles, rhizosphere soil (RS); empty circles, root endophytes (RE). (D) pie graph showing the similarities in the family level distribution of protist affected (enriched/depleted, *P* < 0.01) bacterial ASVs detected by DESeq based on the absolute abundances in RS (outer circle) and its corresponding RE (inner circle) communities in each treatment. Each protist treatment compared with its corresponding control. The data is the total number of ASVs and combination of enriched and depleted taxa showing the top 30 families. See Figs. S7–S8 for enriched and depleted ASVs, separately. (**E**) matrix showing the total numbers of shared protist-affected (enriched and depleted) ASVs between RS and RE treatments. The gradient orange color indicates the higher numbers in each row. See Fig. S11 for the separate results of the enriched and depleted ASVs. **f**, Protist affected RE ASVs and their distribution in the RS. The data shows the absolute abundance of protist-enriched and –depleted endophytic ASVs compared to the control treatment in RE and its corresponding RS communities. Asterisk indicates a significant difference from the control treatment (Kruskal-Wallis test; ***, *P* < 0.001; **, *P* < 0.01, *, *P* < 0.05; NS, *P* > 0.05). See Figs. S11–S13 for detailed explanation and the dataset. (**G**), Venn diagram showing overlap of the protist enriched and depleted RE ASVs among the three soils for each protist. See Fig. S23 for those of RS ASVs. All of the data presented in this fig. Calculated based on the absolute abundances. See Fig. S6 for the results based on the relative abundances. **d-g** represent the results incorporating combined data from the 3rd and 6th weeks.

Each soil consisted of different amplicon sequence variants (ASVs) and the bacterial communities of RS and RE, as well as the enriched and depleted ASVs showed similarities with previous reports on rice plants [16, 23] (Supporting results; Figs. S1–S5, Tables S1–S8). Protists did not have a significant effect on the alpha diversity (Fig. S2). Analyses based on both relative and absolute abundances showed that protists had a significant effect on bacterial community compositions (Tables S1, S5) in both RE (Tables S3, S7) and RS (Tables S4, S8). Principal coordinate analysis (PCoA) based on Bray–Curtis distance (both relative and absolute abundances) revealed that the protist treatments in both RS and RE formed distinct clusters from the CK treatment (Figs. 1A–C, S6A–C). The pattern of separation was consistent in both RS and RE in each soil (Figs. 1A–C, S6A–C) and the two-way PERMANOVA analysis on protists and habitats showed that protist-habitat interaction was less significant (Tables S2, S6), meaning protists had similar and consistent effects on both RS and RE communities. To further support this finding, we analyzed protist-enriched and -depleted ASVs by comparing each protist treatment with the control (combined data from the 3rd and 6th weeks) separately for RS and RE communities in each soil using the differential abundance analysis (DESeq) based on both relative and absolute abundances. To reveal protist-affected taxa, we focused on ASVs rather than higher taxonomic units due to the predation dynamics of protists as they selectively feed on bacteria at the genus or even species level [2, 5, 24]. Then, the ASVs were grouped at family, class, or phylum levels. We first compared the protist-affected (enriched and depleted) ASVs between RS and its corresponding RE communities, which showed similarities at family (Figs. 1D, S6D, S7–S8) and class (Figs. S9–S10) levels. As we obtained similarities between RS and RE in each protist treatment, we produced a matrix that represents the shared protist-affected ASVs between RS and RE samples for each treatment (Figs. 1E, S6E, S11–S12). RS, along with its associated RE, always exhibited the highest number of shared protist-affected ASVs compared to other RE treatments, indicating the similarities of the effects of protists on the RS and RE communities within each soil, which supports our first hypothesis: protist predation upon rhizobacteria alters the community composition of RE bacteria.

We asked whether protist-affected ASVs belonging to RE showed a similar distribution in RS. First, we created an ASV table exclusively consisting of protist-affected RE ASVs. To see the distribution of protist-affected ASVs in all treatments, we prepared relative (Figs. S13–S15) and absolute abundances (Figs. S16–S18) of protist-enriched and depleted RE ASVs in each treatment. A summarized results focusing on the protist-enriched and depleted RE ASVs compared to the CK treatment in RE and RS communities showed that the protist-enriched/depleted RE ASVs also significantly enriched/depleted in their corresponding RS (Figs. 1F and S6F). Similar patterns were also confirmed with the relative abundance data (Fig. S6F). Taken together, all three protists significantly affected RE bacterial communities, which originated in the RS. Therefore, we thought that the effect of protists on endophytic communities could be predicted by their effect on the RS communities. To verify this, we used random forest (RF) analysis [25]. RF predictions are based on splitting data into two parts: training data, and test data, where the results obtained from the training data are used to validate the results on the test data [25, 26]. However, in this specific context to confirm that the effects of protists on RE communities could be predicted by their effect on RS communities, we trained RF with RS samples, and the RE samples were used as test data. The CK treatment (without protists) had a prediction accuracy of 75.8%. The presence of protists increased the prediction accuracies (*P* < 0.01), especially Ac (91.9%) followed by Hg (83.4%), and Vv (79.2%), whereas the prediction accuracy was lower in the non-related treatments (Fig. S19). Protist-affected ASVs in each soil were further classified using RF predictions in each soil to confirm DESeq-based results (Figs. 1D-E and S6D-E), which reproduced similar patterns (Figs. S20–S21), re-confirming our first hypothesis.

### Chaos in the roots

Using three soil types allowed us to evaluate whether the same protist isolates have deterministic effects on the RS and RE communities. Deterministic effects of protists on RE communities were evaluated by comparing the effects of each protist (the enriched and depleted ASVs) in the three soils. Over 90% of the protist-enriched and -depleted ASVs were specific to each soil (Figs. 1G, S6G, S22–S23). This could be a result of ASVs not being present in different soils due to distinct initial bacterial communities; however, on average more than 60% of the protist-affected ASVs were present in all soil types (Fig. S24). Indeed, several ASVs that enriched or depleted in one soil showed an opposite trend in the other two soils (Figs. S25–S26). Further, the enriched and depleted ASVs were assigned to families, and even at the family level, the similarly affected families in three soils were only 4.2% (Figs. S27–S28), indicating that the protist isolates did not have a deterministic effect on RS and RE communities across different soil types.

Although the selective feeding trait of protists may indicate a deterministic mechanism, the deterministic effect of a predatory protist can demonstrate unpredictable outcomes across varying environmental conditions owing to its sensitivity to initial conditions, which was previously shown as *deterministic chaos* [8]. In our study, initial conditions were different on many levels including different bacterial communities, soil nutrients, and soil physical properties. Among them, soil nutrients are recognized as a key factor for the chaotic patterns observed in prey–predator interactions [8]. Therefore, an additional experiment with the bacterial community of Soil 1 was conducted to further confirm that protists modulate RS and RE communities through the intricate dynamics of deterministic chaos, which can be triggered by the dilution rates of initial nutrient media [8]. The experimental setup was the same as the first experiment, except that herein, instead of soil, we used calcined clay as an inert soil substitute allowing us to precisely control nutrient levels [15, 27] (Kimura B nutrient solution [pH: 5,8] with dilution rates of ×1.00, ×0.75, and ×0.50). Based on chaos theory [6], a small change in the initial condition such as the dilution rate of the nutrient media can lead to unpredictable results in prey–predator interactions [8]. Therefore, we hypothesized that the dilution rate of the nutrient solution would trigger chaos resulting in protists affecting different bacterial taxa and forming distinct bacterial communities in each dilution rate.

**Figure 2:**
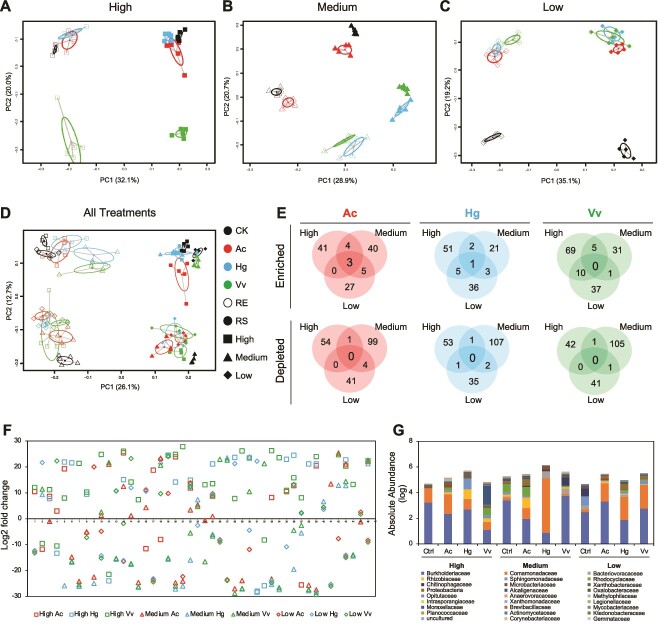
**Trophic modulation of RS and RE bacterial communities by protists in three different nutrient levels based on absolute abundances of bacterial taxa. A-d**, principal coordinate analysis (PCoA) based on the bray–Curtis dissimilarity calculated from the log transformed absolute abundances of bacterial taxa for dilution rates of ×1.00 (**A**, high,), ×0.75 (**B**, medium,), and ×0.50 (**C**, low) of the nutrient media (Kimura B nutrient solution [pH: 5,8]) showing the effect of protists with confidence ellipses based on the eigenvalues of the covariance matrix (**d**, all treatments together). Black, control without protists (CK); red, *Acanthamoeba castellanii* (Ac); blue, *Heteromita globosa* (Hg); green, *Vermamoeba vermiformis* (Vv); filled circles, rhizosphere soil (RS); empty circles, root endophytes (RE). **E**, Venn diagram showing overlap of the protist enriched and depleted RE ASVs detected by DESeq based on the absolute abundances among the three dilution rates for each protist. See Fig. S34 for those of RS ASVs. (**F**) fold change of 46 RE ASVs that showed chaotic patterns (enriched or depleted at one dilution rate, each also exhibiting a contrasting trend in at least one of another dilution rates). Each value was calculated comparing each protist treatment with its corresponding control in each dilution rate using DESeq based on the absolute abundances. (**G**) the absolute abundances of the 46 RE ASVs that showed chaotic patterns in RE communities grouped at the family level. See Fig. S36 for the absolute abundances of the 46 RE ASVs in RS communities. All of the data presented in this fig. Calculated based on the absolute abundances. See Fig. S29 for the results based on the relative abundances.

The protists significantly altered the RS and RE communities (Figs. S29–S30 and Tables S9–S16). The PCoA analysis based on absolute and relative abundances revealed that the protist treatments in both RS and RE formed distinct clusters from the CK treatment and the pattern of separation was consistent in both RS and RE in each dilution rate (Figs. 2A–C, S29A–C), which was similar to results obtained in the first experiment (Figs. 2A–C, S6A–C). A matrix representing the shared protist-affected (enriched/depleted) ASVs between RS and RE samples for each treatment also confirmed the results obtained in the first experiment (Figs. S31–S32). To verify the hypothesis that protists would affect different bacterial taxa and form distinct communities in each dilution rate, we first made a PCoA analysis based on absolute abundances with all of the dilution rates, in which RS and RE communities were grouped into two; however, no consistent pattern was observed for neither protists nor dilution rates (Figs. 2D). PCoA analysis based on relative abundance data also showed similar results (Fig. S29D). Later we checked the protist-affected RE ASVs in each dilution rate for each protist isolate using DESeq, considering both relative and absolute abundances, as previously explained. Protist isolates affected distinct RE ASVs in each dilution rate and the number of commonly enriched/depleted ASVs in all dilution rates was zero, except for Ac-enriched ASVs that was below 10% (Figs. 2E, S29E, S33–S34). Taken together, the results confirmed our hypothesis that the dilution rates of the nutrient solution triggered chaotic dynamics in the trophic interaction between protists and bacteria, resulting in protists affecting different bacterial taxa and forming distinct RE communities in each dilution rate. To delve deeper into the analysis of ASVs exhibiting chaotic dynamics, we identified ASVs that significantly enriched or depleted at one dilution rate, each also exhibiting a contrasting trend in at least one of the dilution rates for each protist (Figs. 2f, S29F). The total absolute and relative abundances of the protist-enriched/depleted ASVs that showed chaotic patterns were more than half in RE (Figs. 2G, S29G) and RS treatments (Figs. S35–S36), indicating the importance of chaos for whole bacterial community composition.

### Species-specific effects of protists on the bacterial communities

**Figure 3:**
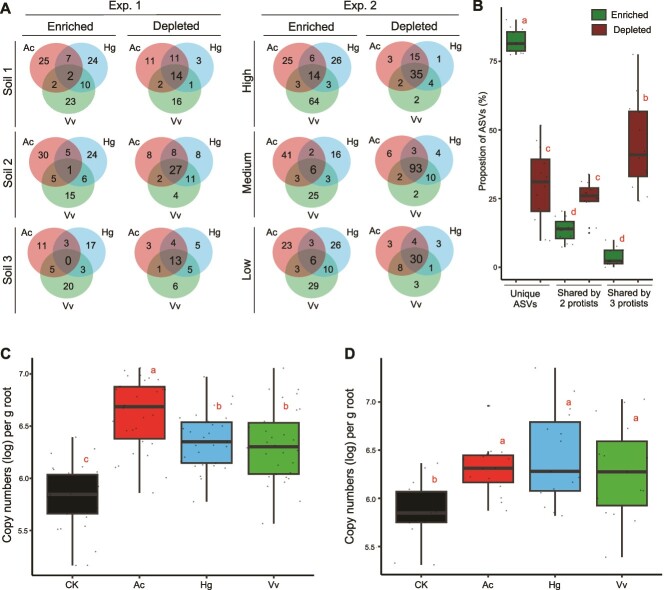
**Species specific effect of protists on bacterial communities and the effect of protists on endophytic bacterial population based on the absolute abundances**. (**A**) Venn diagram showing overlap of the protist enriched and depleted RE ASVs detected by DESeq based on the absolute abundances among the three protists in each dilution rate. See Figs. S39 and S41 for those of RS ASVs. (**B**) box plot representing within group variance of unique and shared ASVs among the three protist treatments. Dark green, enriched ASVs, dark red, depleted ASVs. The data is represents all treatments from both Experiments 1 and 2. **c-d**, box plot representing within group variance of 16S rRNA gene copy numbers in RE treatments from Experiments 1 (**C**) and 2 (**D**). See Figs. S42–S43 for detailed results. Black, control without protists (CK); red, *Acanthamoeba castellanii* (Ac); blue, *Heteromita globosa* (Hg); green, *Vermamoeba vermiformis* (Vv). (**B, C, D**) the central line in the boxplot represents the median, box hinges represent first and third quartiles. Lines indicate minimum and maximum values. Different letters represent significant differences (*P* < 0.05, ANOVA with Tukey's post hoc test). All of the data presented in this figure was calculated based on the absolute abundances. The results of Experiment 1 represent the results incorporating combined data from the 3rd and 6th weeks.

To reveal the species-specific effect of the three protist isolates, we first checked unique and shared ASVs among the protist treatments in each condition (Fig. S37), which showed that nearly half of the ASVs were unique to the treatments in RS and RE. We then compared the Ac-, Hg-, and Vv-enriched and depleted ASVs in the two experiments with relative and absolute abundances, which showed consistent results for RS and RE samples. In both experiments, over 60% of the ASVs were unique to each protist, whereas less than 15% of the ASVs were shared by all protists (Figs. 3A, S38–S41), indicating that protists showed species-specific effects on shaping RS and RE communities. Moreover, we observed a significant difference between the enriched and depleted ASVs: protist-enriched ASVs showed a stronger species-specific effect consistently in both experiments (Figs. 3A-B and S38–S41) with over 75% ASVs being unique, whereas the depleted ASVs showed significantly higher similarities among the three protist treatments (Figs. 3B, S40C). Although protists selectively feed and have different prey preferences, preferred prey species by protists may overlap to some extent [1, 2, 28], which could explain the relatively higher similarities of the depleted ASVs among the three protist treatments. However, to the best of our knowledge, protists showing stronger species-specific effects on the enriched ASVs is a new result. A most known mechanism of protists to enhance bacterial growth is nutrient turnover: the release of excreted nutrients through protist-predation that can be used by the remaining bacteria [2]. The content and amount of the excreted nutrients depend on the protists’ nutritional needs, which can be species-specific [29]. Therefore, we can speculate that even if different protists feed on the same bacterial taxa, the excreted nutrients may vary among the nutritional needs of protist species, resulting in a stronger species-specific effect on the enriched taxa, rather than depleted taxa. Further studies should confirm our findings, which could enhance our understanding on prey–predator interactions and their effects on the prey communities.

### Protists enhance the endophytic population

The number of protists did not differ based on the dilution rates of nutrients (Fig. S42A). The bacterial gene abundances in RS samples were not affected by the protists (Fig. S3). Despite the decrease in preyed bacterial populations due to protist predation, the nutrients released by protists serve as resources for other bacterial species, reducing competition and allowing compensation [2, 5]. Therefore, despite protist predation, the preyed bacteria were likely replaced by predation-resistant species [2, 5, 17]. The balance between losses and gains in the bacterial taxa could also explain why alpha diversity indexes were unaffected by the protists. The bacterial gene abundances in the RE samples were significantly higher in the protist treatments compared to the control in both experiments (Figs. 3C-D, S3, S42B), confirming our 2^nd^ hypothesis that the presence of protists would enhance the root colonization. Although this is the first reference for the increased endophytic bacterial population by the predators, previous studies showed that protists increase the population of bacterial species known to be endophytic, such as *Azopsirillum* sp. [19] and *Pseudomonas* spp. [30]. Escaping from predators is among the most often used survival mechanism by bacteria [31]. We suspect that the presence of the predators, protists, may propel bacteria to escape into the plant roots that can be exclusively used by endophytic bacteria as a protective shelter. Two primary bacterial traits often needed for endophytic colonization are motility and production of exopolysaccharides (EPS) [13, 32], and both motility and EPS production are well-known strategies to survive protist predation [31]. Therefore, bacteria with root colonization traits potentially have an important chance to survive protist predation by escaping into the roots, which is likely to be the main reason for the increased endophytic population in the presence of predators. In addition, protists enhance the biomass of rice plants [11], which was also observed in our experiments (Fig. S43). The increase in plant biomass can influence the endophytic root colonization through higher root density and increased amount of root exudates [13]. Although the mechanism of the protist-enhanced endophytic population remains to be explored, the effects of protists showed deterministic patterns under varied environmental conditions (Figs. S3, S42D). As endophytes play important roles in enhancing plants’ immunity, nutrient acquisition, and tolerance to environmental stresses [13], our finding indicates that protists could be an important facilitator for improved plant health and resilience, as well as enhanced crop productivity.

## Conclusion

In conclusion, here we provided an insight into underground plant-microbe interactions, where predatory protists modulate root endophytes with chaotic dynamics triggered by soil nutrients in microcosm experiments. Under natural conditions, the soil bacterial communities are already under the influence of predatory protists, and when the roots elongate into the soil, they face already protist-modulated communities that are most likely shaped with the chaotic dynamics. Acknowledging and understanding chaos in ecology is essential for advancing our knowledge of complex ecological systems such as prey–predator interactions, predicting their behavior, and managing them sustainably in the face of environmental change [6–8], which can lead to open new avenues for modulating the root microbiome to enhance crop production.

## Supplementary Material

Supplementary_Information_wrae235

## Data Availability

The raw sequence data obtained in this study have been deposited in the NCBI database under the BioProject ID PRJNA1106748.

## Code availability

The codes were provided with the supplementary information.
