# Supplementary material for: Trophic modulation of endophytes by rhizosphere protists
Source: ISME J. 2024 Nov 20;18(1):wrae235. doi: 10.1093/ismejo/wrae235 (PMC11630321; doi:10.1093/ismejo/wrae235)
Supplement: Supplementary_Information_wrae235 [file supplementary_information_wrae235.pdf]

Supporting Information for

Trophic modulation of endophytes by rhizosphere protists

Rasit ASILOGLU[1*], Seda Ozer BODUR[2], Solomon Oloruntoba SAMUEL[3], Murat AYCAN[2], Jun MURASE[4], Naoki HARADA[1]

1. Institute of Science and Technology, Niigata University, Niigata 950-2181, Japan
2. Graduate School of Science and Technology, Niigata University, Niigata 950-2181, Japan
3. Department of Plant and Microbial Biology, North Carolina State University, Raleigh, NC 27695, USA
4. Graduate School of Bioagricultural Sciences, Nagoya University, Nagoya 464-8601, Japan

***Corresponding author:** Rasit ASILOGLU

Address: Laboratory of Applied Protistology, Institute of Science and Technology, Niigata University, 8050 Ikarashi 2-no-cho, Nishi-ku, Niigata 950-2181, Japan

TEL: +81-25-262-6670

Email: asiloglu@agr.niigata-u.ac.jp

This PDF file includes:

Supporting materials and methods

Supporting Results

Figures S1 to S43

Tables S1 to S16

SI References

Codes

SI Materials and Methods

Soil samples and sterile rice seedlings

Soil samples were obtained from at least five locations in each field. The soil samples for each soil type were sieved (<2 mm), individually mixed to homogenize, and then stored at 4 °C. The details of sampling sites and the physicochemical properties of the soils were published elsewhere[1]. Briefly, Soil 1 had relatively higher silt content (33.8%), and Soil 3 had relatively higher clay content (41.5%), while the sand content of Soil 2 (48%) was higher than the other soils. Total Carbon and Nitrogen contents of soil 3 (45.7 and 4.0 mg g $soil^{-1}$) were almost 4 times more than those of soil 1 and 2. The organic matter content of Soil 3 was higher than those of soil 1 and 2. The pH of all soils did not significantly differ among the soil types (ranged from 5.05 to 5.66).

The outer layer of the rice seeds was removed and the seeds were disinfected with 0.2 M sodium hypochlorite (NaClO) by shaking for 5 min at 90 rpm. Next, the seeds were soaked in 70% EtOH for 60 s and then washed with sterile ultra-pure H_2O in a new sterile tube for 5 min at 90 rpm. After three-time washing, the seeds were sowed in MS agar media (Sigma-Aldrich) in a sterile growth bottle in a growth chamber at 24 °C in the dark. After germination, the light condition was set to 250 µmol m^{-2} s^{-1} with a day length of 12 h. The total period from sowing to transplanting was 14 days.

Protist-free bacterial community and the axenic protist isolates

The protist-free indigenous bacterial community was obtained by a filtration method (1.2 µm pore size mixed cellulose ester membrane filters [Advantec, Tokyo, Japan]) from the collected paddy field soils as described previously [2]. Briefly, 300 mL ultra-pure H_2O was added to 200 g of the soil. We used a blender to mix the soil followed by sonication, as it showed a higher recovery of bacterial cells from the soil. Then the slurry mixture was shaken for an hour at 170 rpm min^{-1} and then filtered (< 500 µm) to eliminate the soil particles. Next, we used a vacuum filter (first 5µm, then 1.2 µm pore size mixed cellulose ester membrane filters [Advantec, Tokyo, Japan]) to separate protists from bacteria. Then the bacterial media was washed 3 times with sterile water by centrifugation (4000 g, 10 mins) to exclude the nutrients that come from the soil. The 50 µL of protist-free bacterial inoculum (n = 96) was cultured in 100 µL of the amoeba saline solution [3] in 96-well microtiter plates for three weeks at 20ºC. The absence of protists was confirmed weekly with an inverted microscope at ×100, ×200 and ×400 magnifications (Nikon Eclipse TE2000-S, Tokyo, Japan).

The protists (*Acanthamoeba castellanii* (30234™) and *Vermamoeba vermiformis* (50256™)) were grown in the PYG medium (ATCC® medium 712) and Modified PYNFH medium (ATCC® medium 1034), respectively, as described on the product sheet supplied by ATCC. Briefly, 0.25 ml peak density (about 2 weeks growth) culture was transferred into 5 ml fresh medium and incubated at 20°C under dark conditions. Prior to the experiment, the cells were centrifuged at 1 000 *g* for 10 mins and washed with sterile water three times to eliminate the nutrients that came from the growth media.

Axenic culture of *Heteromita globosa* (Rhizaria; Cercozoa) (~10 µm) that was previously isolated from a paddy field soil[4] was obtained as follows. First, *H. globosa* was grown for two weeks on autoclave-killed bacteria (*Escherichia coli* MG1655). Then the media was centrifuged at 1 000 *g* for 10 mins to separate bacteria and *H. globosa* cells. The supernatant was re-incubated with a mixture of antibiotics (Polymyxin B [50 mg L^{-1}], Streptomycin [50 mg L^{-1}], and Ampicilin [50 mg L^{-1}]) to kill the co-isolated bacteria in the media. Long incubation of *H. globosa* with antibiotics killed the protist, therefore, after 24 hours, 0.5 µL of media was transferred to a PYG medium (ATCC® medium 712) with autoclave-killed bacteria (*Escherichia coli* MG1655). After the growth of *H. globosa,* the 24-hour antibiotic application were repeated 3 times. Then, *H. globosa* cells were centrifuged at 1 000 *g* for 10 mins and washed with sterile water three times to separate the dead bacteria from protist cells and to eliminate the nutrients that came from the growth media. The absence of alive bacteria was checked with the following method: 500 µL of the washed *H. globosa* culture was added to the 1% agar media (Wako Pure Chemical Industries Ltd., Tokyo, Japan) containing either 1/10 TSA, lysogeny broth (LB), or nutrient broth (18 g L^{-1}) (Eiken Chemical Co. Ltd., Tokyo, Japan) media. Three bacterial growth media was used because they can support a wide range of different bacterial growth. Then we checked the absence of bacterial colonies twice a week for three weeks of incubation at 30ºC in the dark.

Sampling of the microcosms

Five replications of the microcosms for each treatment were destructively sampled at 3rd and 6th week. The sampling of each microcosm was performed as follows. The surface water of the microcosms was removed, and the shoots were cut off. The dry plant biomass was measured after drying the shoots at 60°C for three days. The whole root system with the surrounding soil was transferred from the microcosms into a 300 mL sterilized beaker after being shaken vigorously by hand to get rid of the non-rhizosphere soil. The rice roots were carefully

collected with the surrounding rhizosphere soil (RS) with a sterile tweezer and put into a sterile centrifuge tube (volume: 50 mL). Twenty mL of sterile H_2O was added to the centrifuge tube in order to wash off the RS surrounding the roots. Then the tubes were shaken in a shaker for 30 mins (90 rpm min^{-1}). The tubes were centrifuged at 1 000 g for 10 mins for separation of the RS from the roots. The roots were taken out to a new centrifuge tube, and the remaining soil was collected as the RS. To obtain endophytic bacterial samples (RE), the collected roots were washed with sterile H_2O and sonicated for 30 s, repeated 3 times. This method has been shown to effectively remove microbes from the root surface[5]. Then, the roots were surface sterilized with $NaCl_4$ (5 mins) and then 70% EtOH (2 mins) followed by three-time washing with sterile H_2O. Afterward, the roots were soaked in liquid nitrogen and crushed to homogenize. The homogenized roots and the collected RS were stored at -80 $^{\circ}$C until DNA extraction.

Molecular analysis

Negative control was used in all steps from the DNA extraction to the PCR analyses to make sure contamination did not occur. Illumina MiSeq sequencing and primary analyses of raw FASTQ data were performed as described previously [4]. The raw sequence data obtained in this study have been deposited in the NCBI database under the BioProject ID PRJNA1106748.

Total bacterial gene abundances were detected by a quantitative real-time PCR (qPCR). The RE communities consisted of mitochondria and chloroplast from the roots ranged from 10% to 70% (results obtained after bioinformatics). Since using a different primer set can amplify mitochondria and chloroplast at different rates, which can mislead the results, the qPCR analysis was conducted with the same primers as used in amplicon sequencing (515F and 806R) without the Miseq barcode adaptor to obtain absolute abundances, which is an often-used method[6, 7] . One µL of the standardized DNA extracts (5 ng μL^{-1}) was used in the qPCR analysis. The qPCR reaction (25 µL) contained 10 pmol of each primer and 5 µL of SsoFastTM EvaGreen supermix (Bio-Rad, Hercules, CA, USA) and ran in a CFX96TM Real-Time System (Bio-Rad). The qPCR program started with an initial denaturation step of 180 s at 95 °C, followed by 40 cycles of denaturation (45 s, 95 °C) and primer annealing (60 s, 60 °C) with a final step of primer extension of 30 s at 72 °C. The quality and size of the generated amplicons were checked by gel electrophoresis and melting curve analysis. Copy numbers for each gene were calculated using a regression equation for each assay relating the cycle threshold (Ct) values to the known number of copies in the standards of *E. coli*

MG1655. The obtained qPCR results were re-calculated based on the results of the taxonomic assignment in order to exclude the sequences identified as mitochondria and chloroplast.

Bioinformatics and Statistics

DADA2 (Callahan et al., 2016) in QIIME2 (version 2021.11) was used for error correction, removal of forward and reverse primers, quality filtering, doubleton removal, and chimera removal of the Illumina amplicon sequences, with reads truncated at 210 bp and 140 bp for forward and reverse reads, corresponding to a quality score > 30, and allowing forward and reverse sequences to overlap > 90 bp. QIIME2's q2-feature-classifier plugin was used for taxonomy assignment against the latest SILVA reference database (release 138.1)[8]. The unidentified sequences and the sequences identified as Archaea, Mitochondria, and Chloroplast were filtered (Qiime taxa filter-table/seq). We obtained 6,502,811 high-quality sequences with a median frequency of 24,282 reads corresponding to 5,545 amplicon sequence variants (ASVs). DADA2 has been used to denoise the paired-end sequences into (ASVs) by random subsampling at 10 000 reads. Two samples (RS community_Soil 1_Vv treatment_3th Week_3th replicate and RE community_Soil 3_Vv treatment_6th Week_1st replicate) with lower than 10,000 reads were excluded from further analyses. Alpha diversity was computed in QIIME2 using diversity metrics of the richness (observed ASVs) and Shannon index. Up to this stage, all samples were analyzed together. However, since the initial bacterial community was different in each soil sample, the rest of the bioinformatics and biostatistics analyses were separately conducted for each soil type by extracting the samples with Qiime2's filter-samples plugin, unless otherwise stated. Qiime2's diversity beta-group-significance plugin was used to calculate the distance matrix (Bray–Curtis). Statistical differences in community composition were assessed using permutational multivariate analysis of variance (PERMANOVA with 999 random permutations) using the adonis function as implemented in the Vegan package, R program version 4.2.2 (2022.10.31; https://www.r-project.org/). Principal coordinate analysis (PCoA) based on the Bray–Curtis distance matrix was used to visualize the bacterial beta diversities using Phyloseq and Vegan packages in R with the betadisper function. First, ASV table was obtained from the Phyloseq package and then Bray–Curtis distance matrix was created using *bcdist* function in Ecodist package. Venn diagram, DESeq and Random Forest analysis were conducted in R. For this, we first obtained Phyloseq objects using the qiime2 derived rooted-tree, table, and taxonomy files, and the metadata file. Unique and shared ASVs among treatments were detected using the microbiome package

in R. Protist-enriched and -depleted taxa were obtained using the DESeq function in the DESeq2 package in the R program, which models raw counts using a negative binomial GLM, taking into account sample library size and the dispersion for each ASV [9, 10]. Although differential abundance analysis tools have limitations, DESeq2 is one of the recommended tools for microbial datasets[11]. Using this model, we compared each protist treatment with the control treatment with both relative and absolute abundances. The DESeq was done individually for RS and RE samples in each soil for each protist treatment.

The absolutes abundances were calculated as follows: First, the ASV table was obtained from the phyloseq object and the relative abundances were calculated. Then, the relative abundances were multiplied with the qPCR results to obtain an absolute abundance ASV table. The absolute abundance-based analyses were conducted as described above with modifications. For PERMANOVA and PCoA analyses, a log transform was applied to the absolute abundance ASV table to handle heteroscedasticity in the data[6] using *log* function in R, then Bray–Curtis distance matrix was prepared. For the DESeq, the ASV table in the phyloseq object was replaced with absolute abundances ASV table without transforming to log values. The rest of the analyses for absolute abundances were conducted as described above unless otherwise stated.

Random forest (RF) analysis[12, 13] was used for two analyses. Since the results of the DESeq showed that protists' effect on bacterial ASVs were similar for RS and RE samples, our first aim in using RF was to test whether protist-modulation of RE communities can be predicted based on the effects of protists on the RS communities. In the first analysis, the RS community dataset was used for training to learn the effects of the protists on the RS bacterial communities, helping the RF model to understand which bacterial ASVs classified in the protist treatments in RS. The RE dataset, on the other hand, was used for testing in order to make predictions using a random forest algorithm. By inputting the RS samples into the trained random forest model, we could obtain predictions for the corresponding and other RE bacterial communities. The OOB estimate for error rate and test set error rate varied with repeated RF analysis, therefore the RF analysis was repeated 5 times. The maximum OOB estimate for error rate was 9.24%. The average values of test set error rate were used to evaluate the prediction accuracy. Our second aim to use RF was to confirm the DESeq results. For this, samples were separated in the same way as the DESeq to compare each protist treatment with the control treatment individually for RS and RE samples in each soil. The number of test samples was 25%. Both RF analyses were conducted in the R program using the *randomForest* function in the

randomForest package (version 4.7-1.1). The significance of each predictor was evaluated by the rfPErmute package (v2.5.1)[14].

All of the statistical analyses were performed in R program version 4.2.2 (2022.10.31; https://www.r-project.org/) unless otherwise specified. For pairwise comparisons, the Kruskal-Wallis test was done using *kruskal.test* function. For ANOVA analysis, first, the normality assumption of the data and homogeneity of variances within each group was confirmed ($P > 0.05$) using the Shapiro-Wilk test and the Bartlett test, respectively. Then ANOVA test was conducted using *aov* function. All of the codes used in this study were provided in a supplementary file named *codes.txt*.

SI Results

Bacterial community composition of the rhizosphere and roots in three soils (Exp. 1)

Each soil consisted of mainly unique amplicon sequence variants (ASVs): 72.8% of the ASVs were unique to the soil types (Fig. S1). PERMANOVA analysis showed that all three soil types have different communities (Table S1), which is in line with our intention to test the hypotheses with different bacterial communities. A comparison of RS and RE communities showed that RS always had higher total and unique ASVs than RE communities (Fig. S1). The richness (ASVs) and Shannon index of the RE community were on average 220 and 6.16 (Fig. S2), which is similar to previous reports, while the RS richness (Fig. S2) was lower than expected[5]. This is probably due to that the filtering method used in this study may have eliminated several rhizobacterial species. Nevertheless, the bacterial gene abundances were higher in the rhizosphere soil than those of the endophytes, which showed similarity to the field conditions in both RE and RS (Fig. S3). The bacterial community composition of both RS and RE showed similar patterns with previous reports[5, 15]. Overall, Proteobacteria (Gamma and Alpha), Bacteroidota (Bacteroidia and Kryptonia), Firmicutes (Clostridia, Negativicutes, Desulfitobacteriia, and Bacilli), Desulfobacterota (Desulfuromonadia and Desulfovibrionia), and Verrucomicrobiota (Verrucomicrobiae) was dominant in our samples (Fig. S4). The bacterial community composition of RS and RE was significantly different (Table S1-2). Enriched and depleted phyla were detected with DESeq analysis. Among the top 10 abundant phyla, Proteobacteria, Bacteroidota, Verrucomicrobiota, and Actinobacteria were enriched in RE, while Firmicutes, Acidobacteriota, Desulfobacterota, Cyanobacteria, and Bdellovibrionota were enriched in RS (Fig. S5), which is consistent with previous reports[5, 15]. We observed slight differences in the enriched and depleted phyla depending on the soil type (Fig. S5).

SI References

1. Bodur SO, Samuel SO, Suzuki K, Harada N, Asiloglu R. Nitrogen-based fertilizers differentially affect protist community composition in paddy field soils. *Soil Ecology Letters* 2024; **6**: 230221.
2. Asiloglu R, Kenya K, Samuel SO, Sevilir B, Murase J, Suzuki K, et al. Top-down effects of protists are greater than bottom-up effects of fertilisers on the formation of bacterial communities in a paddy field soil. *Soil Biol Biochem* 2021; **156**: 108186.
3. Page FC. A new key to freshwater and soil gymnamoebae: with instructions for culture. *Freshwater Biological Association*. 1988. Ambleside, p 121.

4. Asiloglu R, Shiroishi K, Suzuki K, Turgay OC, Murase J, Harada N. Protist-enhanced survival of a plant growth promoting rhizobacteria, Azospirillum sp. B510, and the growth of rice (Oryza sativa L.) plants. *Applied Soil Ecology* 2020; **154**: 103599.
5. Edwards J, Johnson C, Santos-Medellín C, Lurie E, Podishetty NK, Bhatnagar S, et al. Structure, variation, and assembly of the root-associated microbiomes of rice. *Proceedings of the National Academy of Sciences* 2015; **112**: E911–E920.
6. Barlow JT, Bogatyrev SR, Ismagilov RF. A quantitative sequencing framework for absolute abundance measurements of mucosal and lumenal microbial communities. *Nat Commun* 2020; **11**: 2590.
7. Tkacz A, Hortala M, Poole PS. Absolute quantitation of microbiota abundance in environmental samples. *Microbiome* 2018; **6**: 110.
8. Quast C, Pruesse E, Yilmaz P, Gerken J, Schweer T, Yarza P, et al. The SILVA ribosomal RNA gene database project: improved data processing and web-based tools. *Nucleic Acids Res* 2012; **41**: D590–D596.
9. McMurdie PJ, Holmes S. Waste Not, Want Not: Why Rarefying Microbiome Data Is Inadmissible. *PLoS Comput Biol* 2014; **10**: e1003531.
10. Love MI, Huber W, Anders S. Moderated estimation of fold change and dispersion for RNA-seq data with DESeq2. *Genome Biol* 2014; **15**: 550.
11. Calgaro M, Romualdi C, Waldron L, Risso D, Vitulo N. Assessment of statistical methods from single cell, bulk RNA-seq, and metagenomics applied to microbiome data. *Genome Biol* 2020; **21**: 191.
12. Breiman L. Random forests. *Mach Learn* 2001; **45**: 5–32.
13. Roguet A, Eren AM, Newton RJ, McLellan SL. Fecal source identification using random forest. *Microbiome* 2018; **6**: 185.
14. Archer E. rfPermute: Estimate Permutation p-Values for Random Forest Importance Metrics. *R package version* 2013; **1.5.2**.
15. Xu Y, Ge Y, Song J, Rensing C. Assembly of root-associated microbial community of typical rice cultivars in different soil types. *Biol Fertil Soils* 2020; **56**: 249–260.

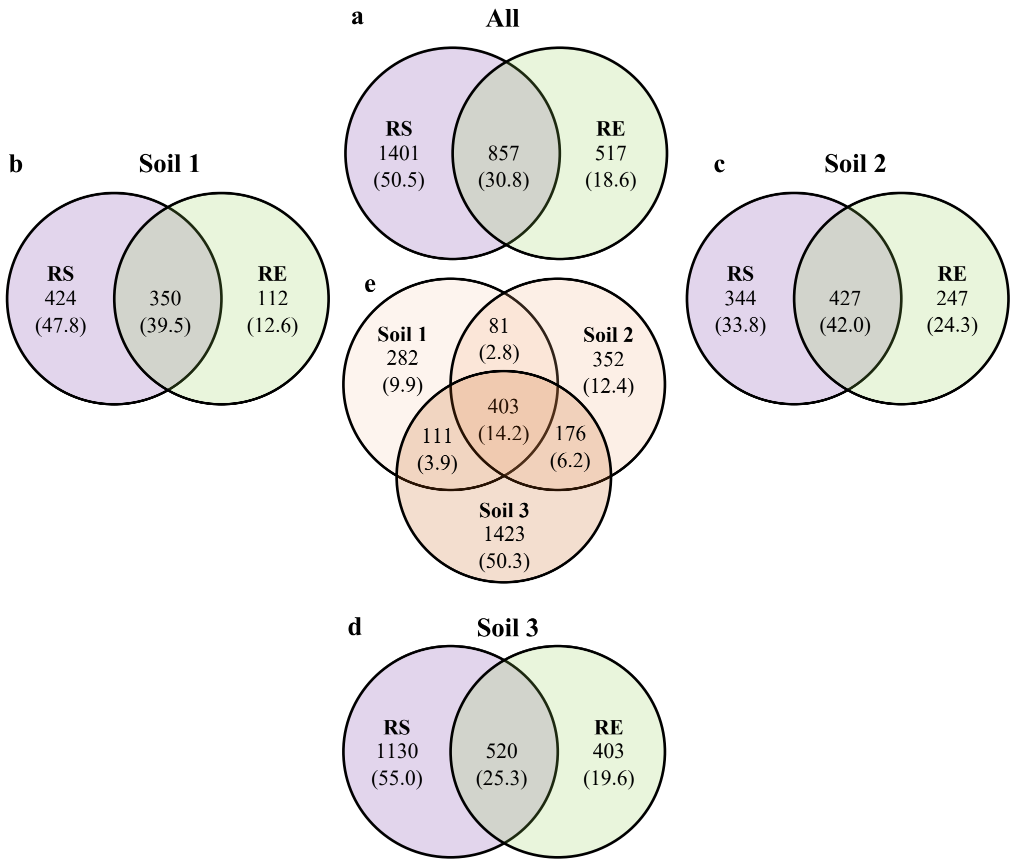

Fig. S1. Venn diagram showing overlap of the ASVs among the communities of rhizobacteria (RS) and root endophytes (RE) in all soils (**a**), Soil 1 (**b**), Soil 2 (**c**), Soil 3 (**d**) and overlap of the ASVs among the three soils (**e**). Numbers indicate total ASVs, number in parentheses indicates percentage values.

Table S1. Permutational multivariate analysis of variance (PERMANOVA) results based on Bray-Curtis dissimilarities calculated from the **relative abundances** of bacterial taxa for the effects of Protists, Soil types, and Habitat (RS and RE).

Factors	Df	SumOfSqs	R2	F	p values	SF
**Protists**	3	2.507	0.050	6.765	0.001	***
**Soil type**	2	8.934	0.180	36.159	0.001	***
**Habitat**	1	2.200	0.044	17.812	0.001	***
**Protists:Soil type**	6	1.620	0.033	2.185	0.001	***
**Protists:Habitat**	3	0.618	0.012	1.667	0.022	*
**Soil type:Habitat**	2	5.438	0.109	22.011	0.001	***
**Protists:Soil type:Habitat**	6	1.980	0.040	2.672	0.001	***
**Residual**	214	26.436	0.532			
**Total**	237	49.732	1.000			

SF, significant factor (***, <0.001; **, <0.01; *, <0.05)

Table S2. PERMANOVA results based on Bray-Curtis dissimilarities calculated from the **relative abundances** of bacterial taxa for the effects of Protists and Habitat (RS and RE).

Factors	Df	SumOfSqs	R2	F	Pr(>F)	SF
**Protists**	3	2.507	0.050	4.331	0.001	***
**Habitat**	1	2.230	0.045	11.561	0.001	***
**Protists:Habitat**	3	0.619	0.012	1.070	0.337	**NS**
**Residual**	230	44.375	0.892			
**Total**	237	49.732	1.000			

SF, significant factor (***, <0.001; **, <0.01; *, <0.05; NS, not significant)

Table S3. PERMANOVA results based on Bray-Curtis dissimilarities calculated from the **relative abundances** of bacterial taxa for the effects of Protists and Soil type on RE community.

Factors	Df	SumOfSqs	R2	F	Pr(>F)	SF
**Protists**	3	1.9192	0.09555	8.9088	0.001	***
**Soil type**	2	7.2297	0.35996	50.3408	0.001	***
**Protists:Soil type**	6	3.2526	0.16194	7.5492	0.001	***
**Residual**	107	7.6834	0.38255			
**Total**	118	20.0849	1			

SF, significant factor (***, <0.001; **, <0.01; *, <0.05; NS, not significant)

Table S4. PERMANOVA results based on Bray-Curtis dissimilarities calculated from the **relative abundances** of bacterial taxa for the effects of Protists and Soil type on RS community.

Factors	Df	SumOfSqs	R2	F	Pr(>F)	SF
**Protists**	3	1.8694	0.10018	8.4743	0.001	***
**Soil type**	2	6.1638	0.33031	41.9128	0.001	***
**Protists:Soil type**	6	2.7595	0.14788	6.2546	0.001	***
**Residual**	107	7.8678	0.42163			
**Total**	118	18.6605	1			

SF, significant factor (***, <0.001; **, <0.01; *, <0.05; NS, not significant)

Table S5. PERMANOVA results based on Bray-Curtis dissimilarities calculated from the log transformed **absolute abundances** of bacterial taxa for the effects of Protists, Soil types, and Habitat (RS and RE).

**Factors**	**Df**	**SumOfSqs**	**R2**	**F**	**p values**	**SF**
**Protists**	3	3.214	0.05925	10.3703	0.001	***
**Soil type**	2	8.494	0.15662	41.1159	0.001	***
**Habitat**	1	10.316	0.1902	99.8644	0.001	***
**Protists:Soil type**	6	4.987	0.09196	8.047	0.001	***
**Protists:Habitat**	3	1.064	0.01963	3.4348	0.001	***
**Soil type:Habitat**	2	2.422	0.04466	11.7244	0.001	***
**Protists:Soil type:Habitat**	6	1.634	0.03012	2.6358	0.001	***
**Residual**	214	22.106	0.40757			
**Total**	237	54.237	1			

SF, significant factor (***, <0.001; **, <0.01; *, <0.05)

Table S6. PERMANOVA results based on Bray-Curtis dissimilarities calculated from the log transformed **absolute abundances** of bacterial taxa for the effects of Protists and Habitat (RS and RE).

**Factors**	**Df**	**SumOfSqs**	**R2**	**F**	**Pr(>F)**	**SF**
**Protists**	3	3.214	0.05925	6.213	0.001	***
**Habitat**	1	10.304	0.18998	59.7633	0.001	***
**Protists:Habitat**	3	1.063	0.01961	2.0558	0.002	**
**Residual**	230	39.656	0.73116			
**Total**	237	54.237	1			

SF, significant factor (***, <0.001; **, <0.01; *, <0.05; NS, not significant)

Table S7. PERMANOVA results based on Bray-Curtis dissimilarities calculated from the log transformed **absolute abundances** of bacterial taxa for the effects of Protists and Soil type on RE community.

**Factors**	**Df**	**SumOfSqs**	**R2**	**F**	**Pr(>F)**	**SF**
**Protists**	3	2.1203	0.10902	7.7742	0.001	***
**Soil type**	2	4.8437	0.24906	26.6402	0.001	***
**Protists:Soil type**	6	2.7568	0.14175	5.054	0.001	***
**Residual**	107	9.7274	0.50017			
**Total**	118	19.4481	1			

SF, significant factor (***, <0.001; **, <0.01; *, <0.05; NS, not significant)

Table S8. PERMANOVA results based on Bray-Curtis dissimilarities calculated from the log transformed **absolute abundances** of bacterial taxa for the effects of Protists and Soil type on RS community.

**Factors**	**Df**	**SumOfSqs**	**R2**	**F**	**Pr(>F)**	**SF**
**Protists**	3	2.1568	0.08809	6.2145	0.001	***
**Soil type**	2	6.0882	0.24865	26.3136	0.001	***
**Protists:Soil type**	6	3.8615	0.15771	5.5633	0.001	***
**Residual**	107	12.3783	0.50555			
**Total**	118	24.4848	1			

SF, significant factor (***, <0.001; **, <0.01; *, <0.05; NS, not significant)

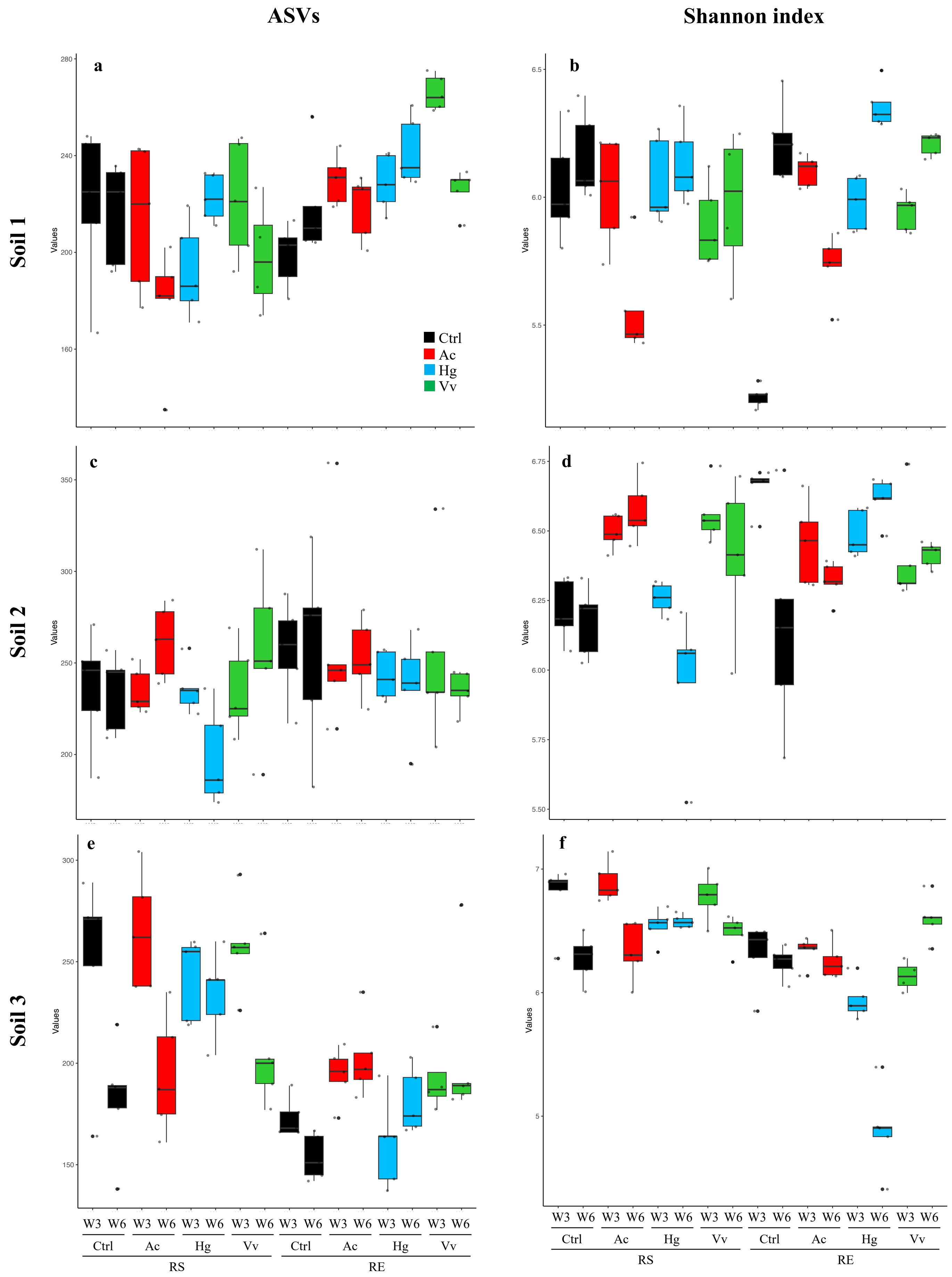

Fig. S2. Box plot representing within group variance of ASVs (**a,c,e**) and Shannon index (**b,d,f**) in soil 1 (**a,b**), 2 (**c,d**), and 3 (**e,f**). The central line in the boxplot represents the median, box hinges represent first and third quartiles. Lines indicate minimum and maximum values. Black, control without protists (Ctrl); red, *Acanthamoeba castellanii* (Ac); blue, *Heteromita globosa* (Hg); green, *Vermamoeba vermiformis* (Vv); RS, rhizosphere soil; RE, root endophytes; W, weeks after incubation.

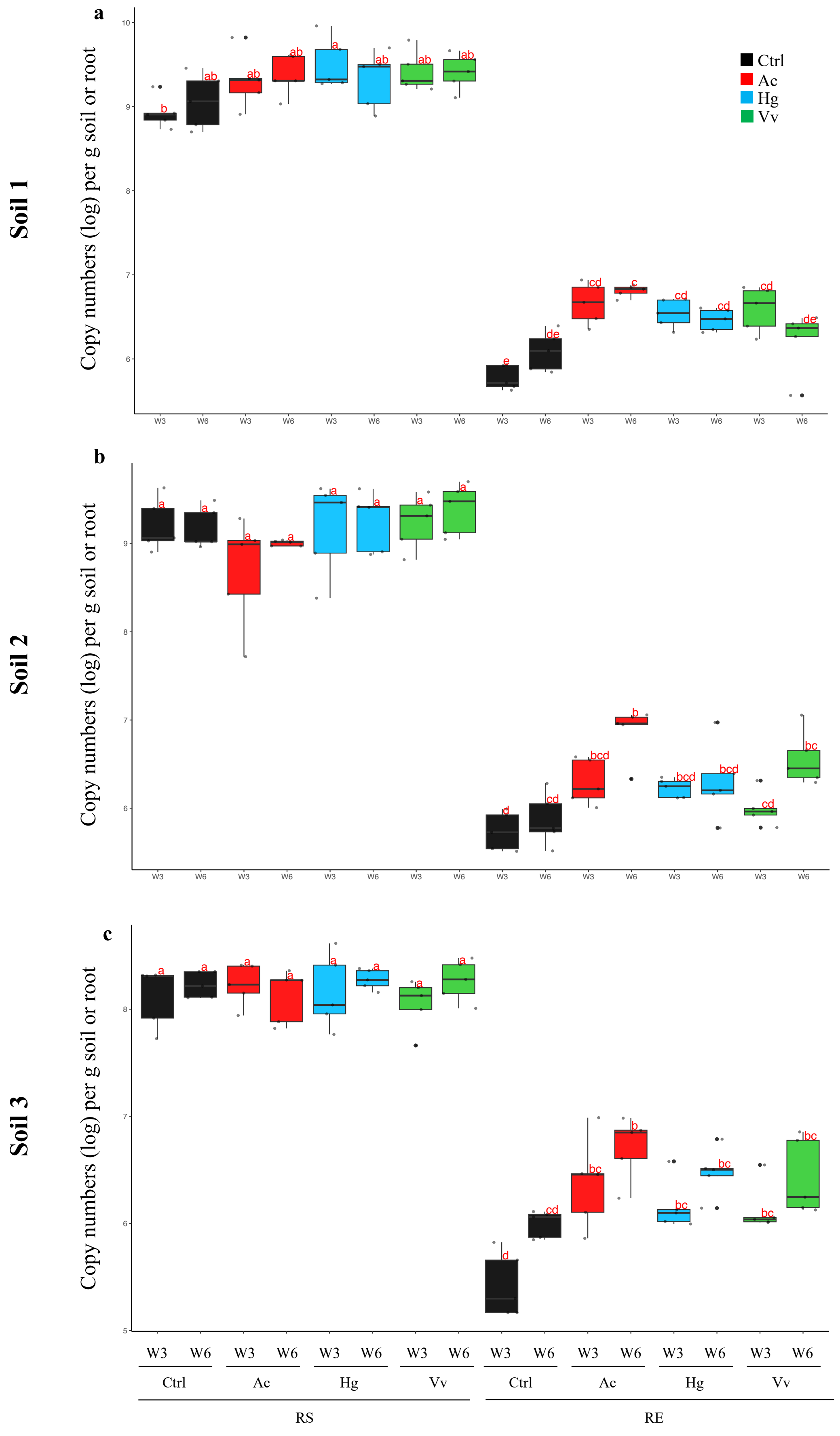

Fig. S3. Box plot representing within group variance of 16S rRNA gene copy numbers in soil 1 (**a**), 2 (**b**), and 3 (**c**). The central line in the boxplot represents the median, box hinges represent first and third quartiles. Lines indicate minimum and maximum values. Different letters represent significant differences (p<0.05, ANOVA with Tukey's post hoc test). Black, control without protists (Ctrl); red, *Acanthamoeba castellanii* (Ac); blue, *Heteromita globosa* (Hg); green, *Vermamoeba vermiformis* (Vv); RS, rhizosphere soil; RE, root endophytes; W, weeks after incubation.

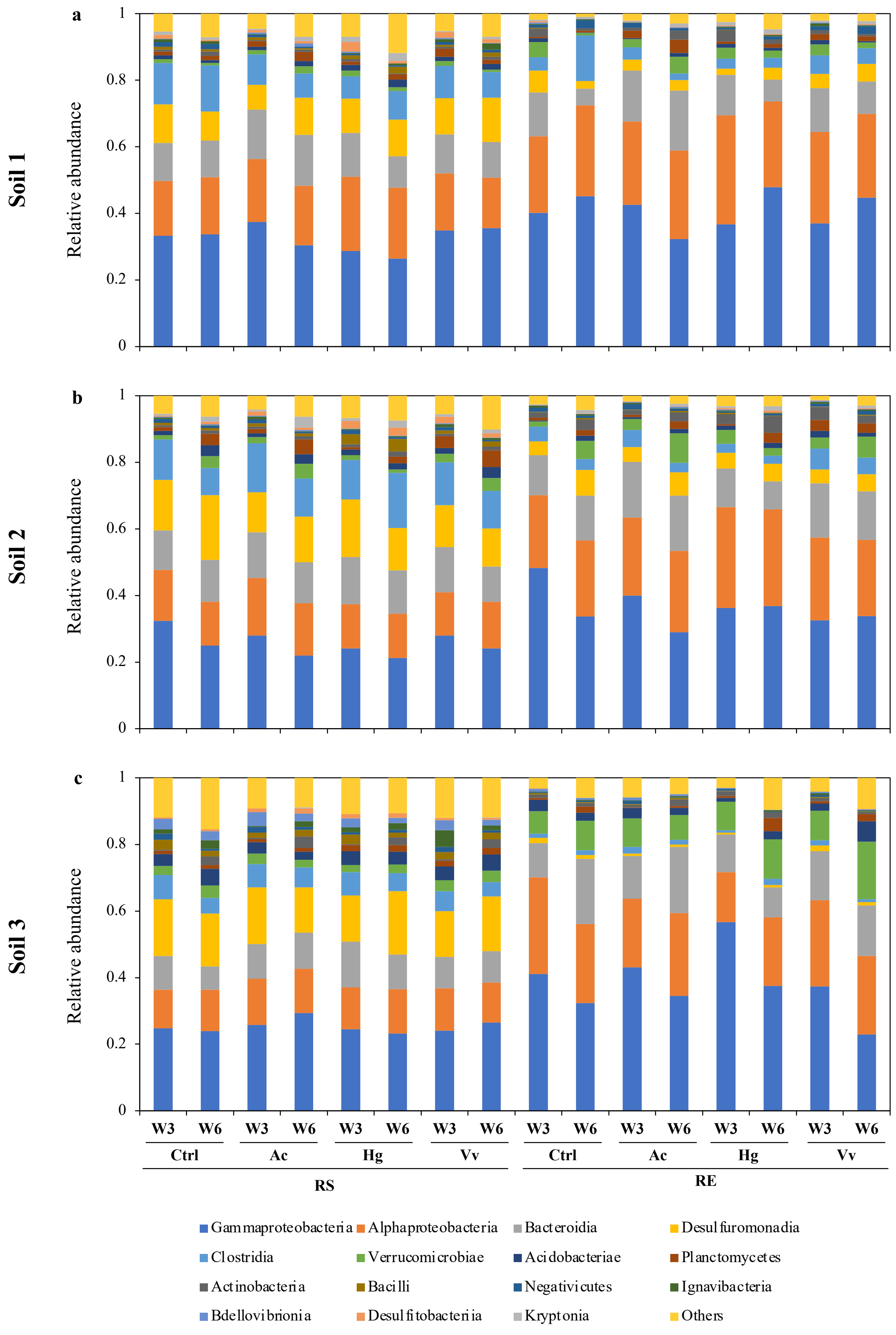

Fig. S4. Taxa barplot representing relative abundance of bacterial taxa at class level (n=5) in soil 1 (**a**), 2 (**b**), and 3 (**c**). Ctrl, control without protists; Ac, *Acanthamoeba castellanii*; Hg, *Heteromita globosa*; Vv, *Vermamoeba vermiformis*; RS, rhizosphere soil; RE, root endophytes; W, weeks after incubation.

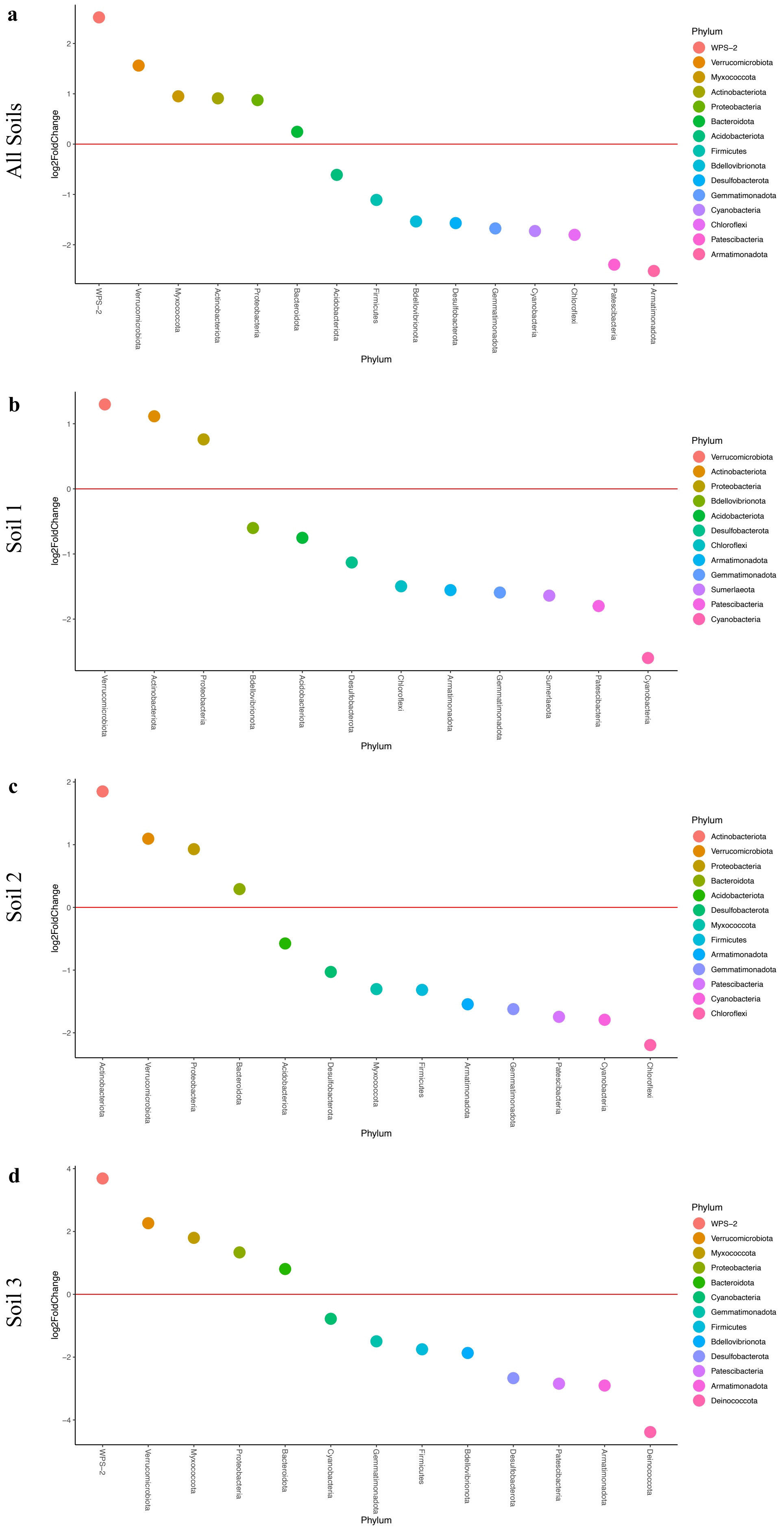

Fig. S5. Pairwise comparison (DeSeq analysis based on relative abundances) of bacterial ASVs in rhizosphere soil (RS) and roots (RE) showing the differential abundant ASVs ($p < 0.01$) in RE communities of all soils (**a**), Soil 1 (**b**), Soil 2 (**c**), and soil 3 (**d**). ASVs were assigned to phylum (x-axis, colors). Positive " log2 Fold Change " values (y-axis) indicate for higher abundance in RE samples and negative values indicate higher abundances in RS samples in all samples, Soil 1, 2, and 3.

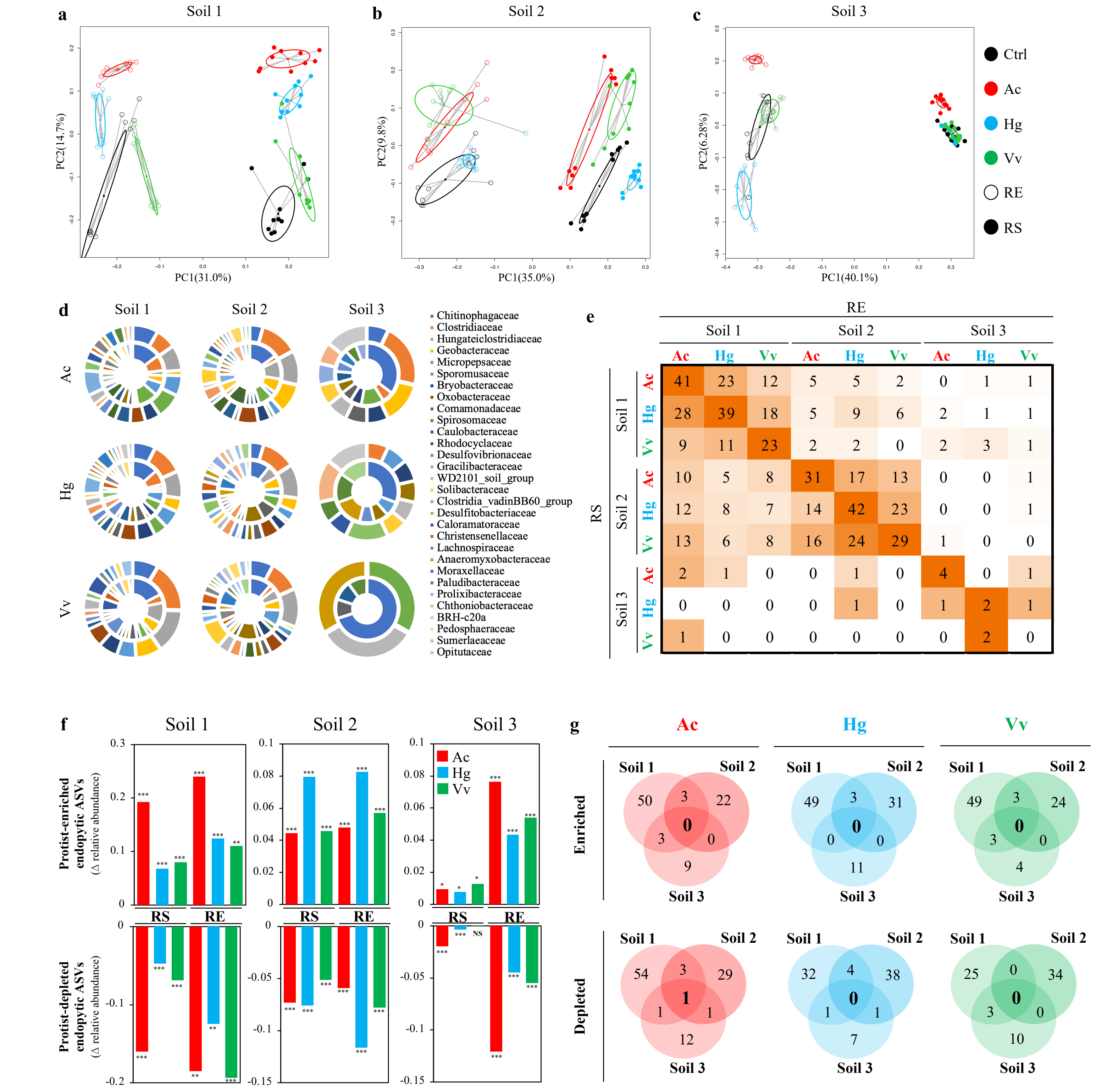

Fig. S6. Trophic regulation of rhizosphere soil (RS) and root endophytic (RE) bacterial communities by protists based on relative abundances of bacterial taxa. **a-c**, Principal Component Analysis (PCA) based on the Bray–Curtis dissimilarity index of the relative abundances of bacterial communities in soil 1(**a**), 2(**b**), and 3(**c**) showing the effect of protists with confidence ellipses based on the eigenvalues of the covariance matrix. Black, control without protists (Ctrl); red, *Acanthamoeba castellanii* (Ac); blue, *Heteromita globosa* (Hg); green, *Vermamoeba vermiformis* (Vv); filled circles, rhizosphere soil (RS); empty circles, root endophytes (RE). **d,** Pie graph showing the similarities in the family level distribution of the protist affected (enriched/depleted, $p<0.01$) bacterial ASVs detected by DESeq based on the relative abundances in RS (outer circle) and its corresponding RE (inner circle) communities in each treatment. Each protist treatment compared with its corresponding control. The data is the total number of ASVs and combination of enriched and depleted taxa showing the top 30 families. See Fig. S7-8 for for enriched and depleted ASVs, separately. **e,** Matrix showing the total numbers of shared protist-affected (enriched and depleted) ASVs between RS and RE treatments. The gradient orange color indicates the higher numbers in each row. See Fig. S9 for the separate results of the enriched and depleted ASVs. **f,** Protist affected RE ASVs and their distibution in the RS. The data shows the relative abundance of protist-enriched and –depleted endophytic ASVs compared to the control treatment in RE and its corresponding RS communities. Asterisk indicates a significant difference from the control treatment (Kruskal-Wallis test [n=10]; ***, $p<0.001$; **, $p<0.01$, *, $p<0.05$; NS, $p>0.05$). See Fig. S11-13 for detailed explanation and the dataset. **g,** Venn diagram showing overlap of the protist enriched and depleted RE ASVs among the 3 soils for each protist. See Fig. S22 for those of RS ASVs.

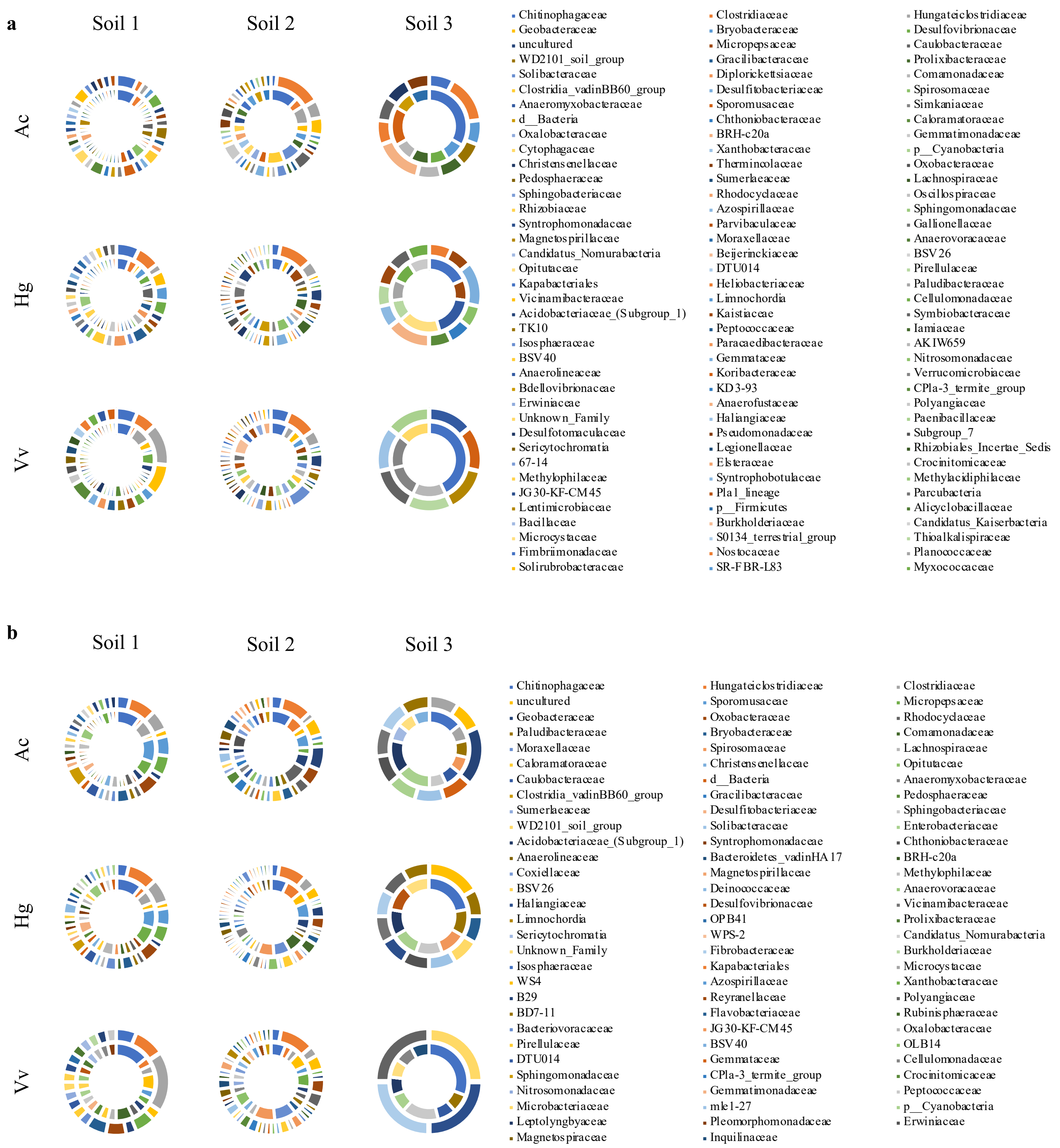

Fig. S7. Pie graph showing the similarities in the family level distribution of protist enriched (**a**) and depleted (**b**)($p<0.01$) bacterial ASVs detected by DESeq based on the **relative abundances** in RS (outer circle) and its corresponding RE (inner circle) communities in each treatment. Each protist treatment compared with its corresponding control. The data is the total number of ASVs grouped at Family level. Colors indicate families.

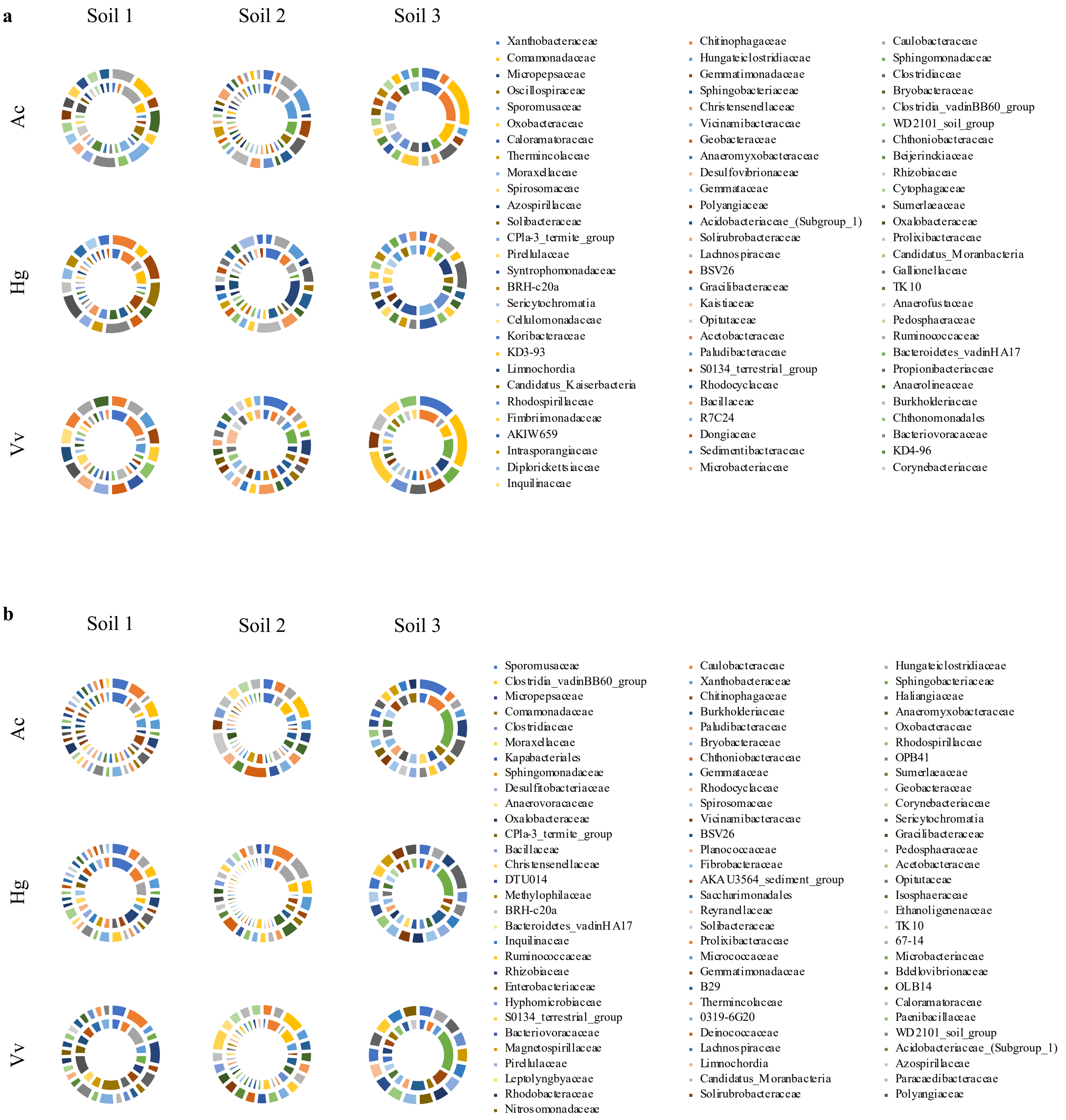

Fig. S8. Pie graph showing the similarities in the family level distribution of protist enriched (**a**) and depleted (**b**)(p<0.01) bacterial ASVs detected by DESeq based on the **absolute abundances** in RS (outer circle) and its corresponding RE (inner circle) communities in each treatment. Each protist treatment compared with its corresponding control. The data is the total number of ASVs grouped at Family level. Colors indicate families.

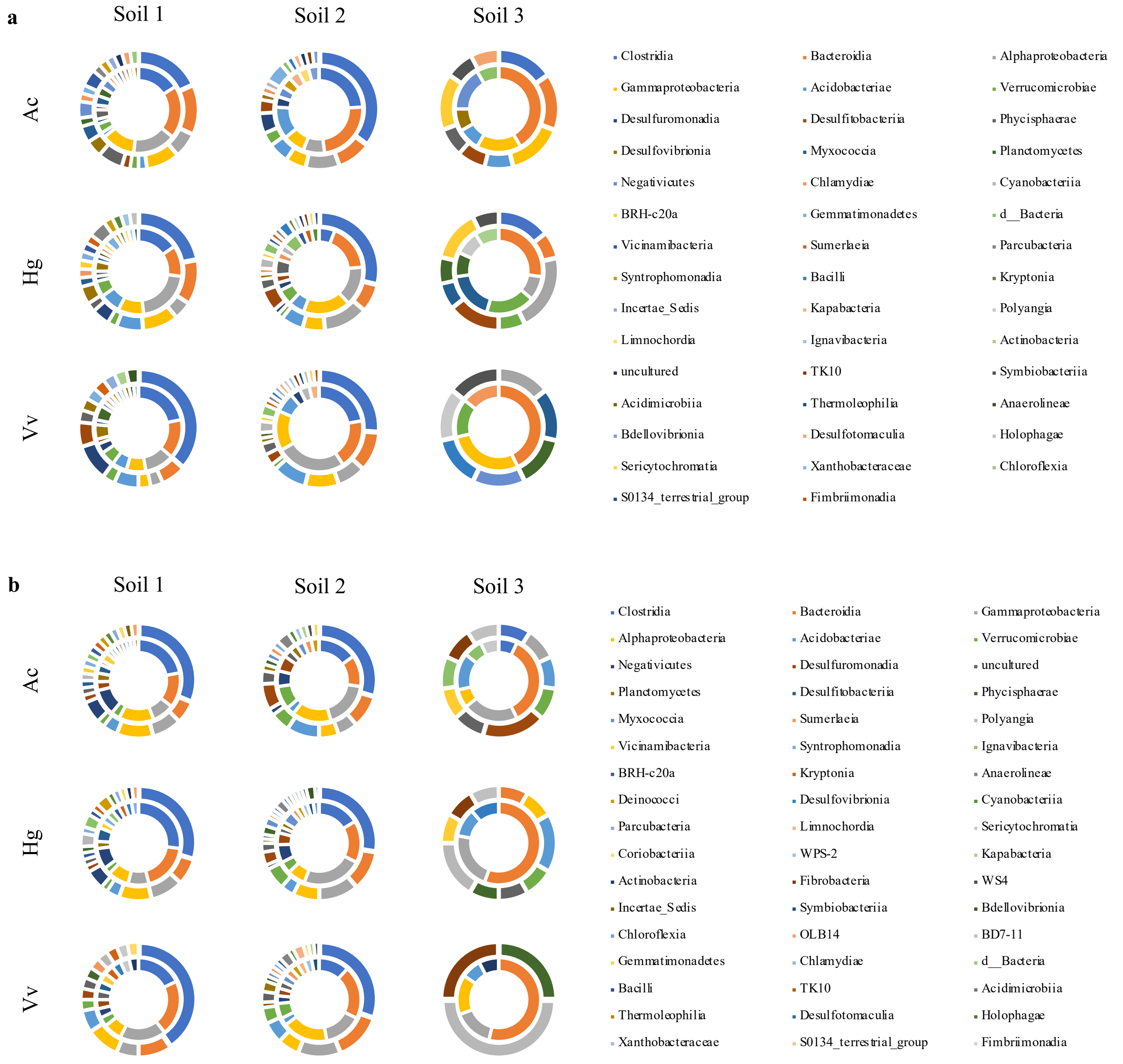

Fig. S9. Pie graph showing the similarities in the class level distribution of protist enriched (**a**) and depleted (**b**)(p<0.01) bacterial ASVs detected by DESeq based on the **relative abundances** in RS (outer circle) and its corresponding RE (inner circle) communities in each treatment. Each protist treatment compared with its corresponding control. The data is the total number of ASVs grouped at Class level. Colors indicate classes.

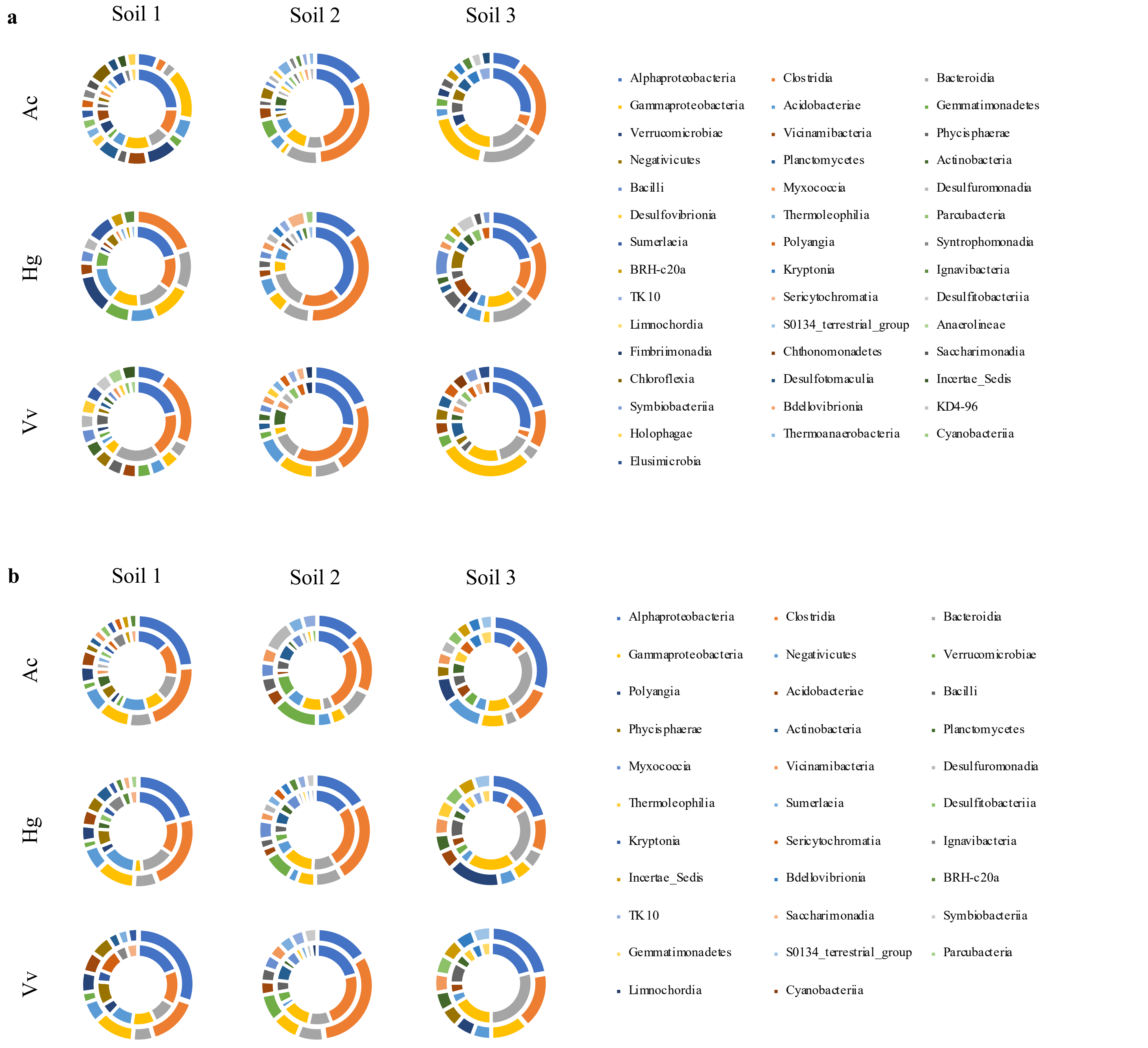

Fig. S10. Pie graph showing the similarities in the class level distribution of protist enriched (**a**) and depleted (**b**)(p<0.01) bacterial ASVs detected by DESeq based on the **absolute abundances** in RS (outer circle) and its corresponding RE (inner circle) communities in each treatment. Each protist treatment compared with its corresponding control. The data is the total number of ASVs grouped at Class level. Colors indicate classes.

a

RS \ RE		Soil 1 Ac	Soil 1 Hg	Soil 1 Vv	Soil 2 Ac	Soil 2 Hg	Soil 2 Vv	Soil 3 Ac	Soil 3 Hg	Soil 3 Vv
Soil 1	Ac	19	7	7	2	2	0	0	1	1
	Hg	8	22	12	1	3	1	2	1	1
	Vv	3	5	16	2	1	0	1	3	1
Soil 2	Ac	4	3	7	15	5	3	0	0	1
	Hg	5	3	5	2	19	6	0	0	1
	Vv	6	2	6	4	9	13	0	0	0
Soil 3	Ac	1	1	0	0	1	0	3	0	1
	Hg	0	0	0	0	1	0	0	1	0
	Vv	1	0	0	0	0	0	0	2	0

b

RS \ RE		Soil 1 Ac	Soil 1 Hg	Soil 1 Vv	Soil 2 Ac	Soil 2 Hg	Soil 2 Vv	Soil 3 Ac	Soil 3 Hg	Soil 3 Vv
Soil 1	Ac	22	16	5	3	3	2	0	0	0
	Hg	20	17	6	4	6	5	0	0	0
	Vv	6	6	7	0	1	0	1	0	0
Soil 2	Ac	6	2	1	16	12	10	0	0	0
	Hg	7	5	2	12	23	17	0	0	0
	Vv	7	4	2	12	15	16	1	0	0
Soil 3	Ac	1	0	0	0	0	0	1	0	0
	Hg	0	0	0	0	0	0	1	1	1
	Vv	0	0	0	0	0	0	0	0	0

Fig. S11. Matrix showing the total numbers of shared protist enriched (**a**) and depleted (**b**) ASVs between RS and RE treatments detected by DESeq based on the **relative abundances**. The gradient green and red color indicates the higher numbers in each row for enriched and depleted ASVs, respectively.

a

RS \ RE		Soil 1 Ac	Soil 1 Hg	Soil 1 Vv	Soil 2 Ac	Soil 2 Hg	Soil 2 Vv	Soil 3 Ac	Soil 3 Hg	Soil 3 Vv
Soil 1	Ac	15	5	2	3	0	0	2	0	2
	Hg	2	8	2	1	3	1	2	1	3
	Vv	3	3	10	1	0	0	1	1	1
Soil 2	Ac	6	8	5	14	4	2	1	0	1
	Hg	2	1	2	3	12	3	0	1	2
	Vv	3	3	2	4	8	10	2	3	3
Soil 3	Ac	2	2	1	2	1	1	3	1	0
	Hg	0	0	2	1	0	3	0	4	1
	Vv	1	1	0	0	0	0	1	2	2

b

RS \ RE		Soil 1 Ac	Soil 1 Hg	Soil 1 Vv	Soil 2 Ac	Soil 2 Hg	Soil 2 Vv	Soil 3 Ac	Soil 3 Hg	Soil 3 Vv
Soil 1	Ac	16	15	7	7	6	4	0	0	0
	Hg	13	15	7	6	8	6	0	0	0
	Vv	7	8	6	5	5	3	0	0	0
Soil 2	Ac	3	1	0	10	11	7	0	0	0
	Hg	2	2	1	12	19	13	0	0	0
	Vv	2	1	0	9	15	14	0	0	0
Soil 3	Ac	3	2	3	1	2	1	3	4	3
	Hg	1	1	1	0	0	0	2	2	1
	Vv	0	0	0	0	0	0	2	1	1

Fig. S12. Matrix showing the total numbers of shared protist enriched (**a**) and depleted (**b**) ASVs between RS and RE treatments detected by detected by DESeq based on the **absolute abundances**. The gradient green and red color indicates the higher numbers in each row for enriched and depleted ASVs, respectively.

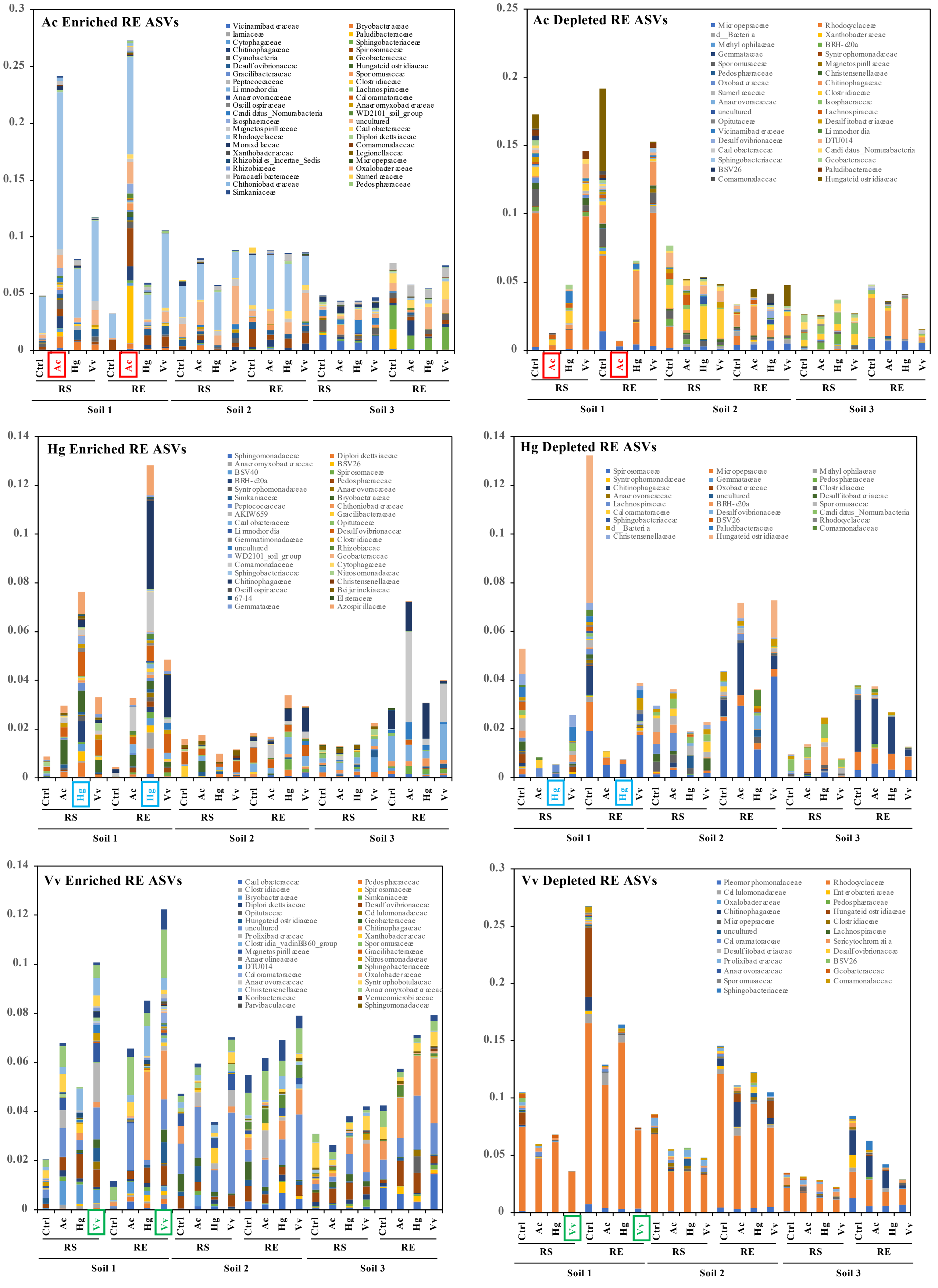

Fig. S13. Relative abundances of the protist enriched (right) and depleted (left) RE ASVs grouped at Family level (colors of taxa bar plot) in each treatment of Soil 1. Each Figure was prepared for enriched/depleted ASVs detected by DESeq based on the **relative abundances** by each protist exclusively in RE samples. The color in the x axis for RE samples indicate the source of the ASVs (where those ASVs were enriched or depleted by represented protist isolate), and the the color in the x axis for RS samples indicate its corresponding rhizosphere. The data represented in **Fig. S6f** was prepared by subtracting the total relative abundance of ASVs in protist treatment (colored samples in X axis) from its control (the first control on the left).

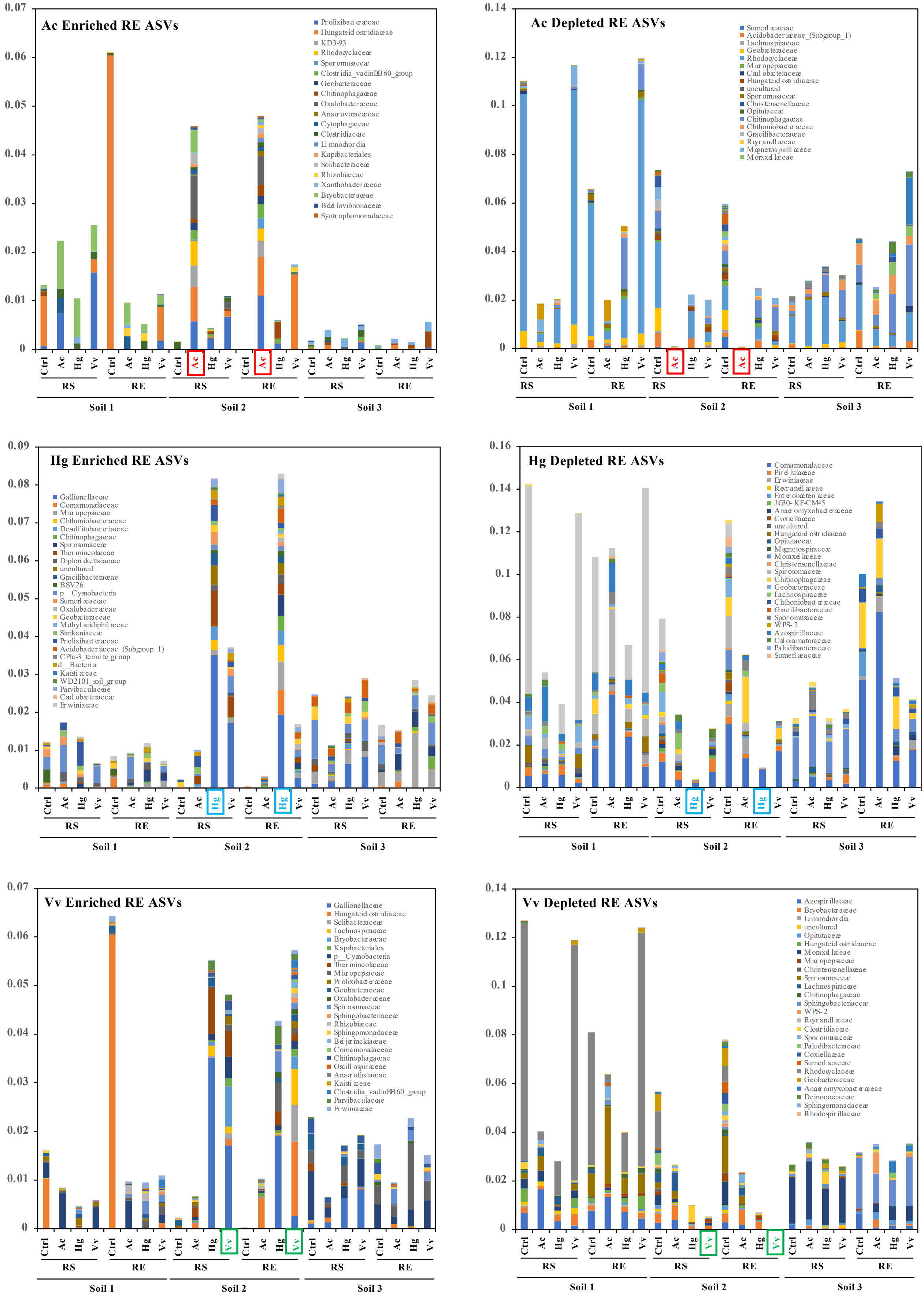

Fig. S14. Relative abundances of the protist enriched (right) and depleted (left) RE ASVs grouped at Family level (colors of taxa bar plot) in each treatment of Soil 2. Each Figure was prepared for enriched/depleted ASVs detected by DESeq based on the **relative abundances** by each protist exclusively in RE samples. The color in the x axis for RE samples indicate the source of the ASVs (where those ASVs were enriched or depleted by represented protist isolate), and the the color in the x axis for RS samples indicate its corresponding rhizosphere. The data represented in **Fig. S6f** was prepared by subtracting the total relative abundance of ASVs in protist treatment (colored samples in X axis) from its control (the first control on the left).

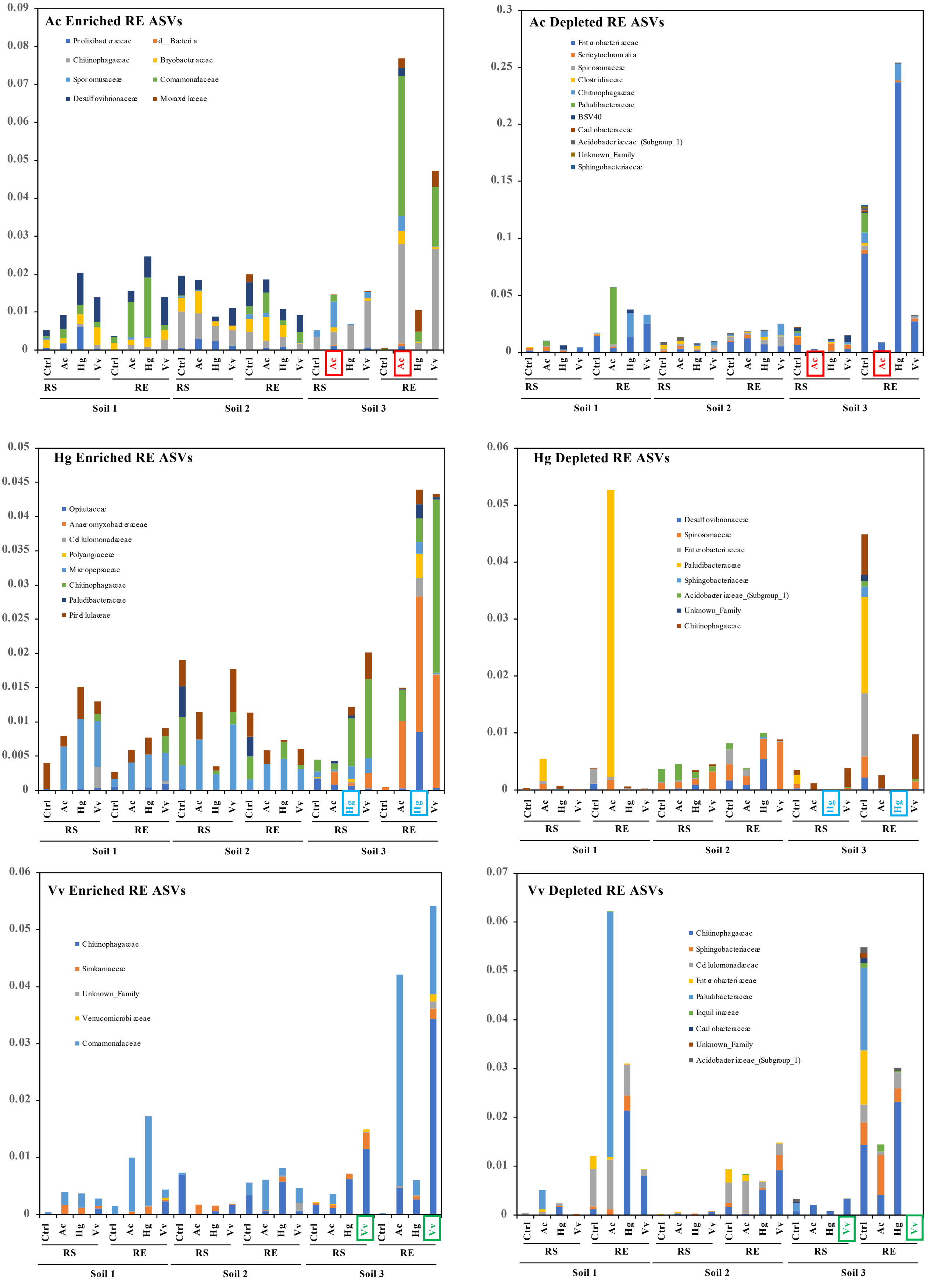

Fig. S15. Relative abundances of the protist enriched (right) and depleted (left) RE ASVs grouped at Family level (colors of taxa bar plot) in each treatment of Soil 3. Each Figure was prepared for enriched/depleted ASVs detected by DESeq based on the **relative abundances** by each protist exclusively in RE samples. The color in the x axis for RE samples indicate the source of the ASVs (where those ASVs were enriched or depleted by represented protist isolate), and the the color in the x axis for RS samples indicate its corresponding rhizosphere. The data represented in **Fig. S6f** was prepared by subtracting the total relative abundance of ASVs in protist treatment (colored samples in X axis) from its control (the first control on the left).

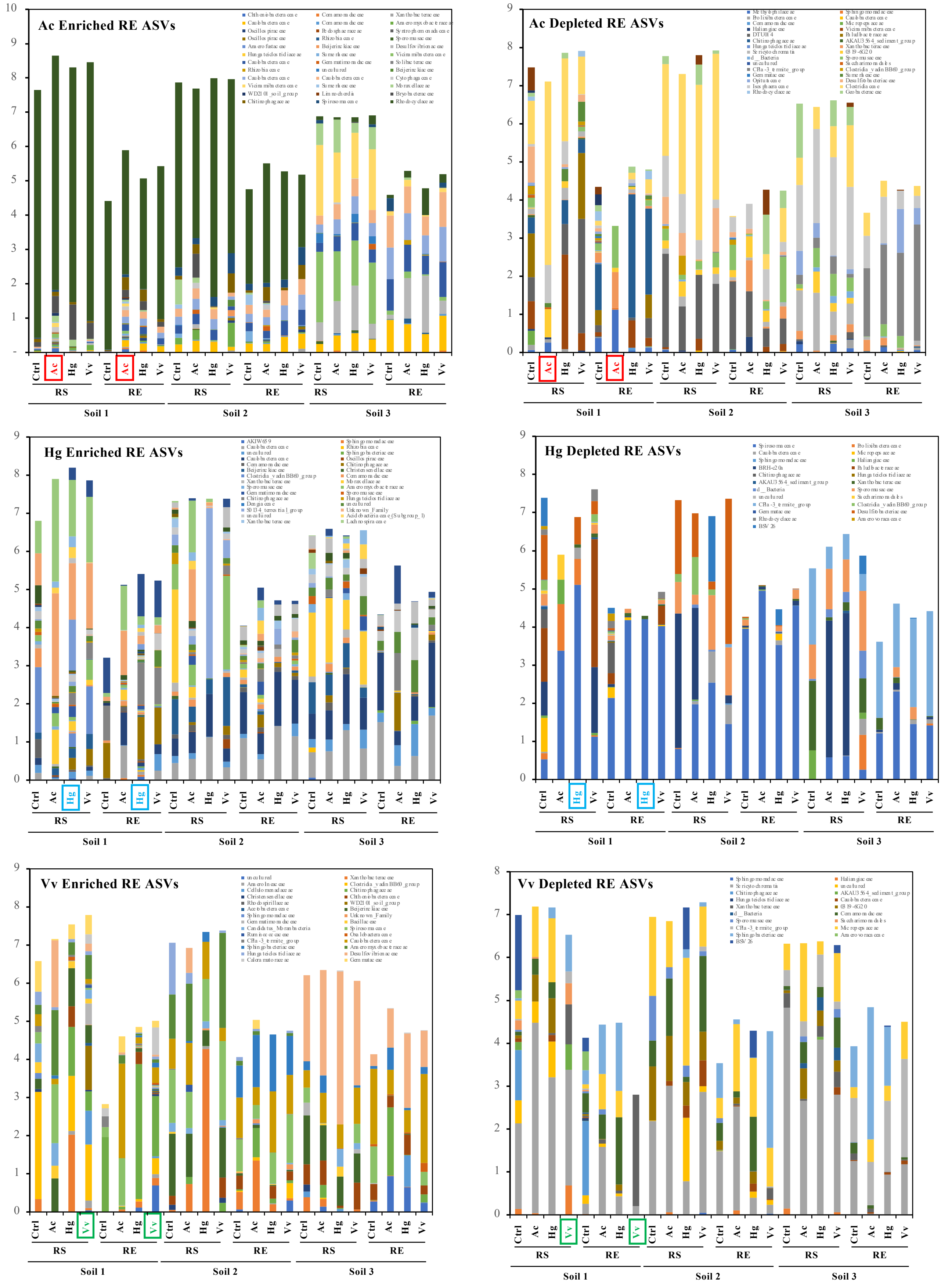

Fig. S16. Relative abundances of the protist enriched (right) and depleted (left) RE ASVs grouped at Family level (colors of taxa bar plot represent relative distribution of each taxa) in each treatment of Soil 1. Each Figure was prepared for enriched/depleted ASVs detected by DESeq based on the **absolute abundances** by each protist exclusively in RE samples. The color in the x axis for RE samples indicate the source of the ASVs (where those ASVs were enriched or depleted by represented protist isolate), and the the color in the x axis for RS samples indicate its corresponding rhizosphere. The data represented in **Fig. 1f** was prepared by subtracting the total relative abundance of ASVs in protist treatment (colored samples in X axis) from its control (the first control on the left).

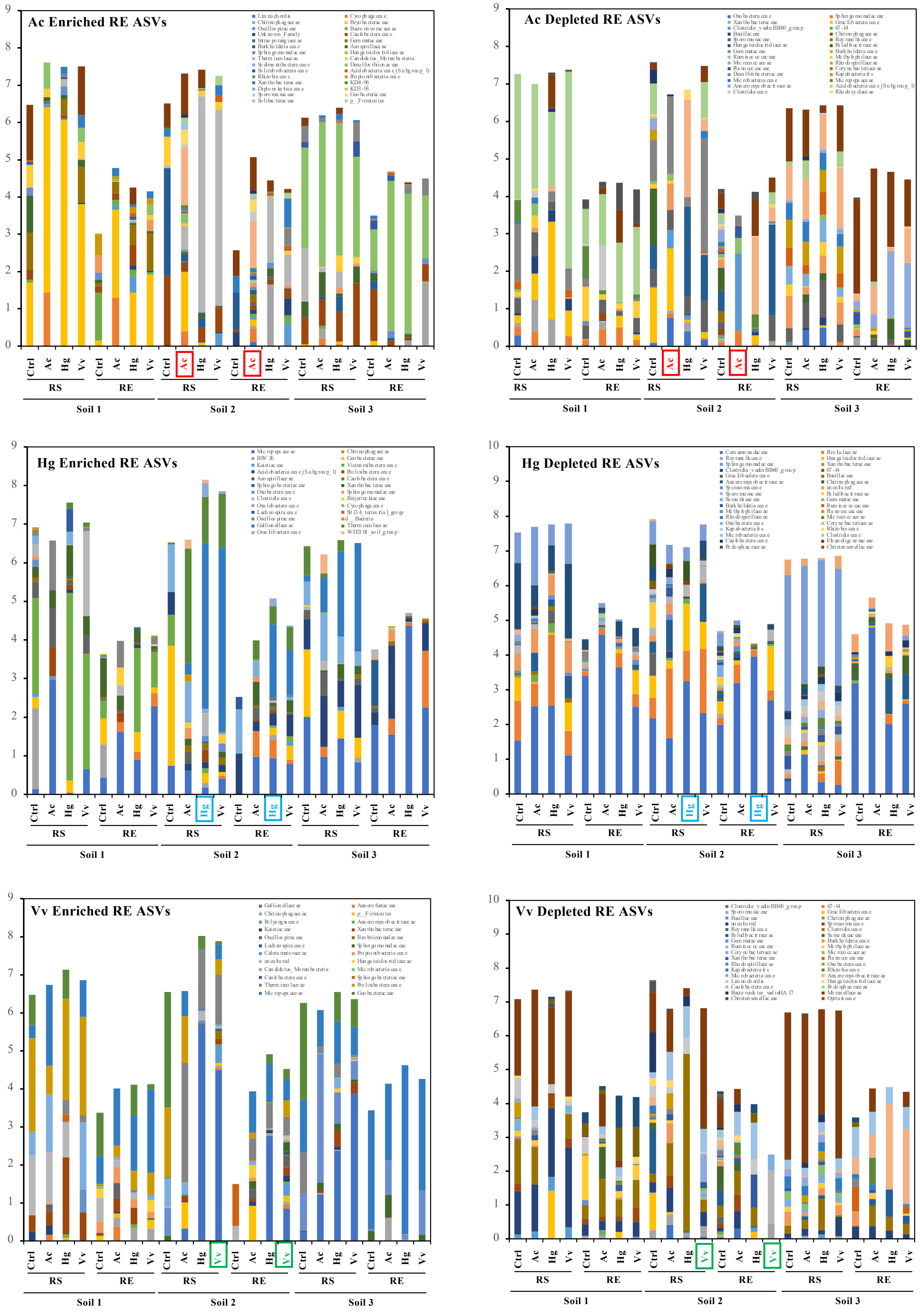

Fig. S17. Relative abundances of the protist enriched (right) and depleted (left) RE ASVs grouped at Family level (colors of taxa bar plot represent relative distribution of each taxa) in each treatment of Soil 2. Each Figure was prepared for enriched/depleted ASVs detected by DESeq based on the **absolute abundances** by each protist exclusively in RE samples. The color in the x axis for RE samples indicate the source of the ASVs (where those ASVs were enriched or depleted by represented protist isolate), and the the color in the x axis for RS samples indicate its corresponding rhizosphere. The data represented in **Fig. 1f** was prepared by subtracting the total relative abundance of ASVs in protist treatment (colored samples in X axis) from its control (the first control on the left).

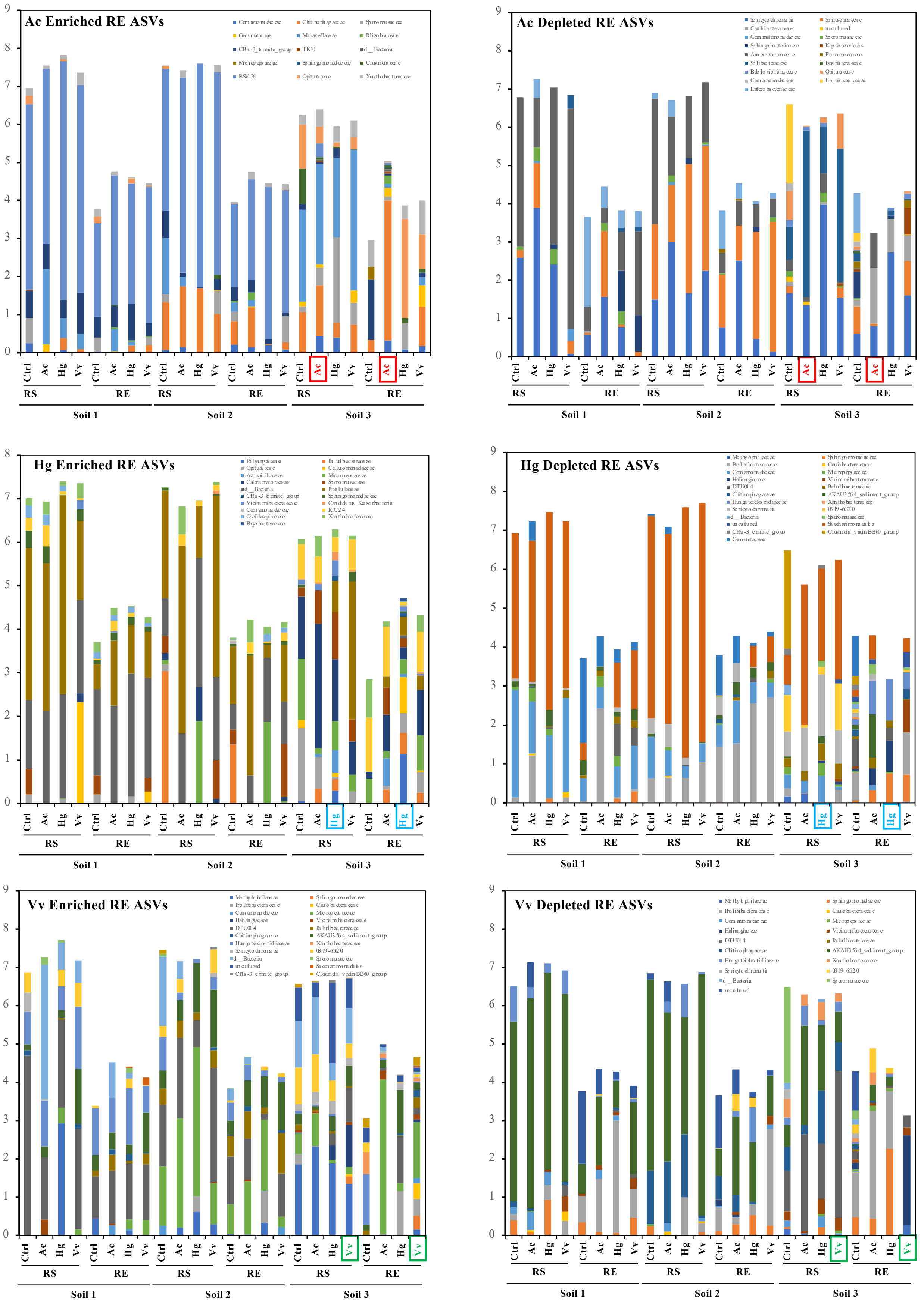

Fig. S18. Relative abundances of the protist enriched (right) and depleted (left) RE ASVs grouped at Family level (colors of taxa bar plot represent relative distribution of each taxa) in each treatment of Soil 3. Each Figure was prepared for enriched/depleted ASVs detected by DESeq based on the **absolute abundances** by each protist exclusively in RE samples. The color in the x axis for RE samples indicate the source of the ASVs (where those ASVs were enriched or depleted by represented protist isolate), and the the color in the x axis for RS samples indicate its corresponding rhizosphere. The data represented in **Fig. 1f** was prepared by subtracting the total relative abundance of ASVs in protist treatment (colored samples in X axis) from its control (the first control on the left).

		RE			
		**Ctrl**	**Ac**	**Hg**	**Vv**
**RS**	**Ctrl**	**75.80**	68.91	67.40	71.26
	**Ac**	57.98	**91.94**	57.81	58.99
	**Hg**	66.73	68.24	**83.36**	68.41
	**Vv**	71.09	69.25	70.76	**79.16**

Fig. S19. Random Forest predictions of the effects of protists on RE communities that was predicted by their effect on the corresponding RS communities. We trained Random Forest with RS samples, and the RE samples were used as test data.

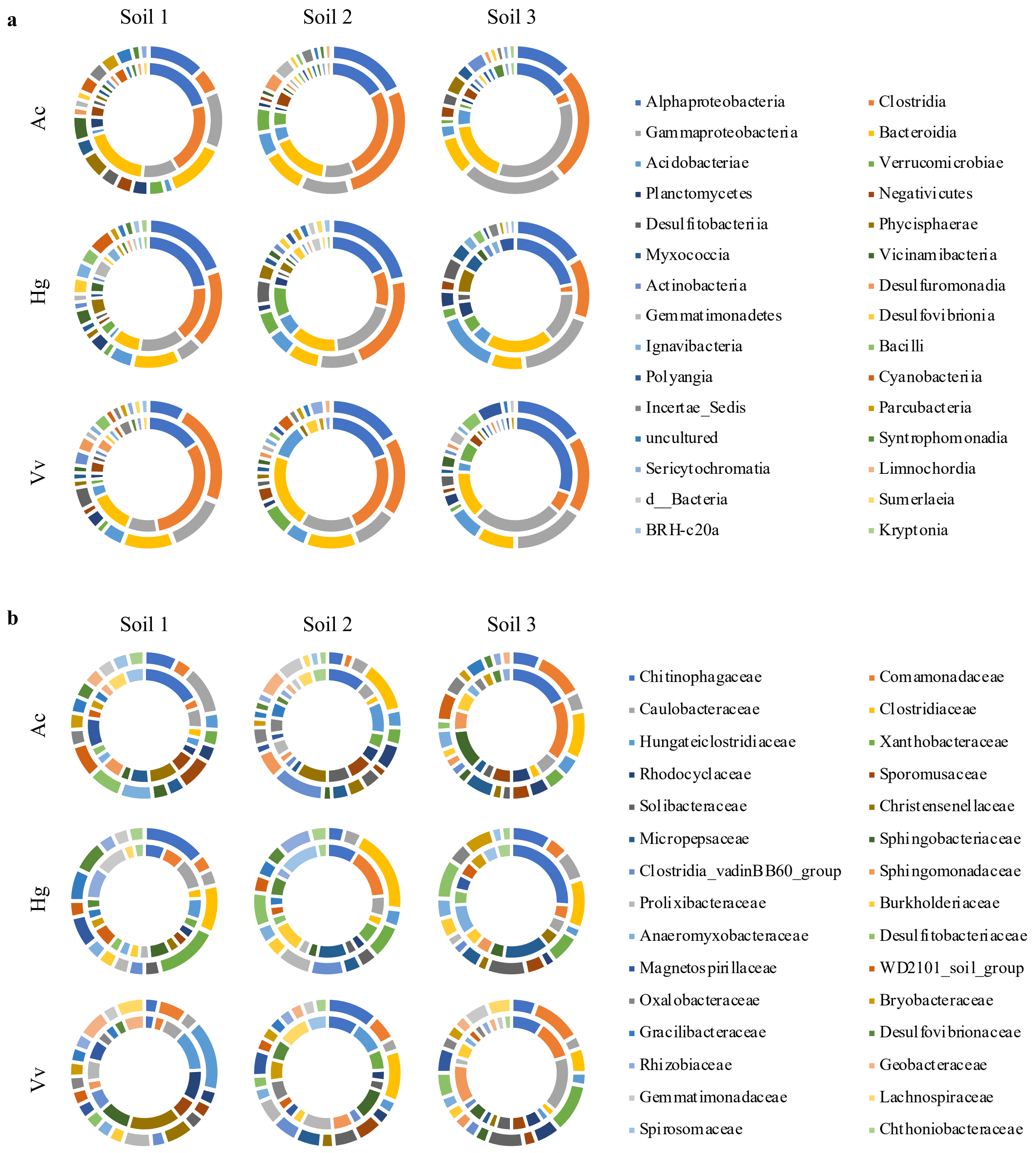

Fig. S20. Pie graph showing the similarities in the Class (**a**) and Family (**b**) level distribution of protist affected ($p<0.01$) bacterial ASVs detected by Random Forest in RS (outer circle) and its corresponding RE (inner circle) communities in each treatment. Each protist treatment compared with its corresponding control. The data is the total number of ASVs grouped at top 20 Classes and top 30 Families.

			RE								
			Soil 1			Soil 2			Soil 3		
			Ac	Hg	Vv	Ac	Hg	Vv	Ac	Hg	Vv
RS	Soil 1	Ac	18	5	3	6	1	4	3	4	1
		Hg	3	21	3	4	7	4	3	2	1
		Vv	4	0	15	6	0	1	4	3	5
	Soil 2	Ac	6	2	4	24	4	1	0	0	1
		Hg	0	8	1	1	20	1	2	1	1
		Vv	4	5	3	4	3	12	3	6	2
	Soil 3	Ac	7	2	3	3	3	1	13	5	4
		Hg	2	4	5	3	7	1	2	10	3
		Vv	0	2	1	2	2	1	1	1	5

Fig. S21. Matrix showing the total numbers of shared protist affected ASVs between RS and RE treatments detected by Random Forest. The gradient orange color indicates the higher numbers in each row for protist affected ASVs.

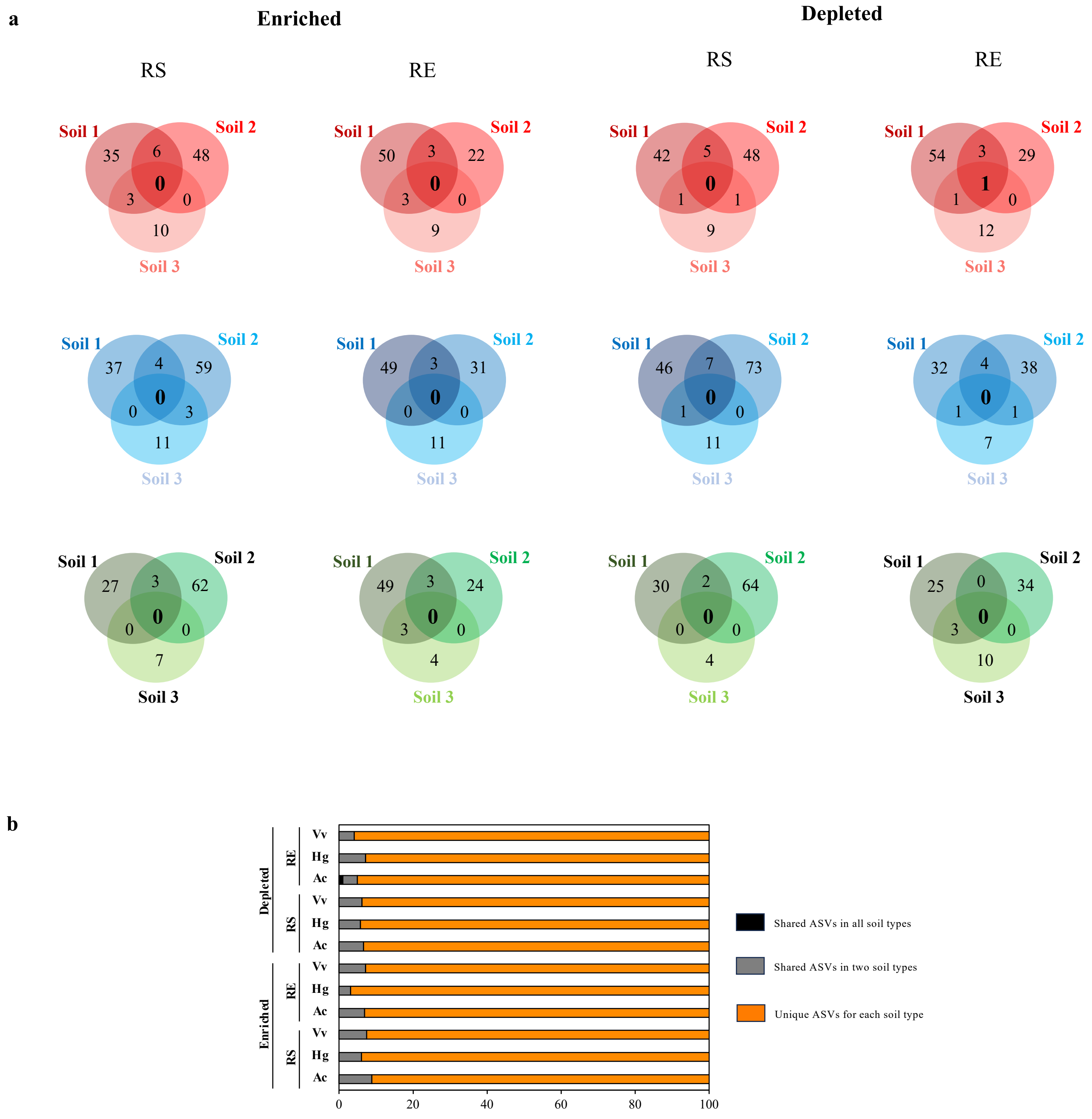

Fig. S22. a, Venn diagram showing overlap of the protist enriched and depleted RS and RE ASVs detected by DESeq based on the **relative abundances** among the 3 soils for each protist. Red, *Acanthamoeba castellanii* (Ac); blue, *Heteromita globosa* (Hg); green, *Vermamoeba vermiformis* (Vv). **b**, the percentage values of unique and shared ASVs.

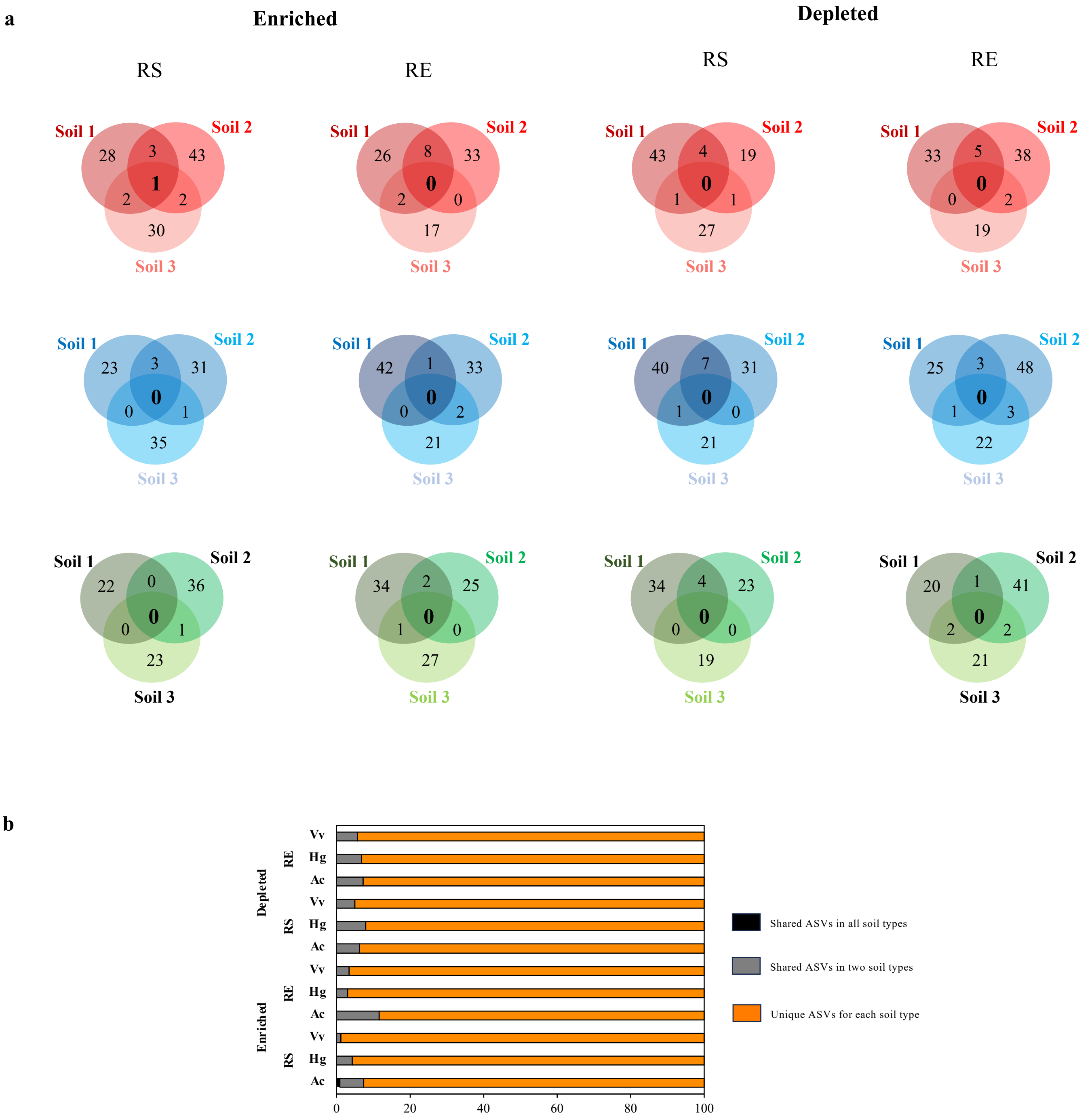

Fig. S23. a, Venn diagram showing overlap of the protist enriched and depleted RS and RE ASVs detected by DESeq based on the **absolute abundances** among the 3 soils for each protist. Red, *Acanthamoeba castellanii* (Ac); blue, *Heteromita globosa* (Hg); green, *Vermamoeba vermiformis* (Vv). **b**, the percentage values of unique and shared ASVs.

a

		Soil 1	Soil 2	Soil 3
Soil 1	Ac	100	67.85714	57.14286
	Hg	100	55.76923	50
	Vv	100	70.90909	63.63636
Soil 2	Ac	40	100	40
	Hg	64.70588	100	73.52941
	Vv	77.77778	100	59.25926
Soil 3	Ac	66.66667	75	100
	Hg	54.54545	45.45455	100
	Vv	66.66667	66.66667	100

b

		Soil 1	Soil 2	Soil 3
Soil 1	Ac	100	69.49153	57.62712
	Hg	100	67.56757	51.35135
	Vv	100	75	71.42857
Soil 2	Ac	72.72727	100	66.66667
	Hg	86.04651	100	62.7907
	Vv	82.35294	100	61.76471
Soil 3	Ac	57.14286	71.42857	100
	Hg	66.66667	66.66667	100
	Vv	61.53846	53.84615	100

Fig. S24. The matrix showing the percentage presence of protist-enriched (**a**) and depleted (**b**) RE ASVs in soils. Columns, ASVs that were enriched/depleted in protist treatments compared to its control, Rows indicates their presence in each soil.

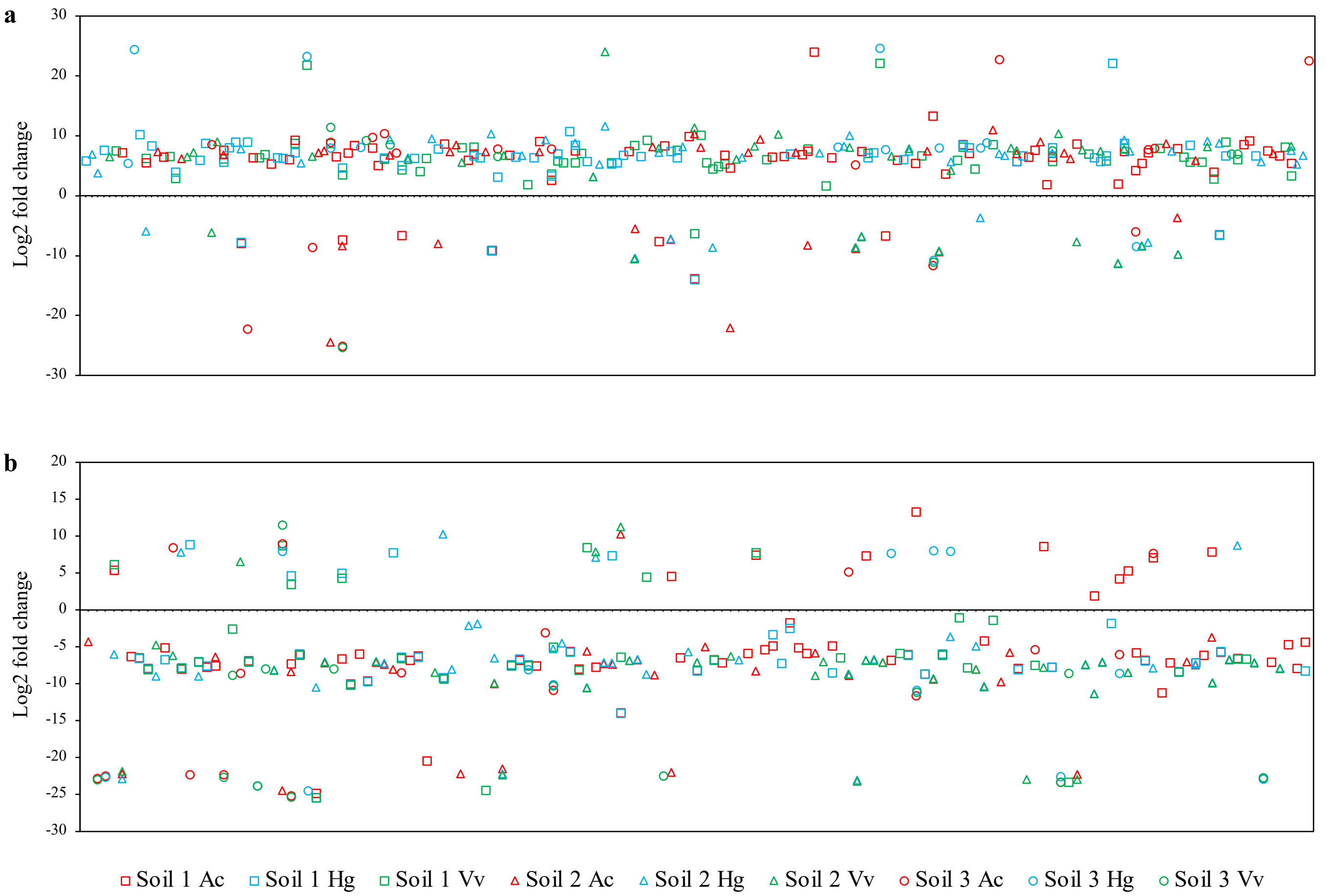

Fig. S25. Chaotic patterns of enriched/depleted ASVs in three soils as some of the enriched/depleted ASVs in one soil showed opposite patterns in other soils. The data shows fold change of the protist enriched (**a**) and depleted (**b**) RE ASVs and their distribution in other treatments. Only significantly enriched/depleted ASVs (p<0.01) detected by DESeq based on the **relative abundances** are shown. Each value was calculated comparing protist treatment with its corresponding control.

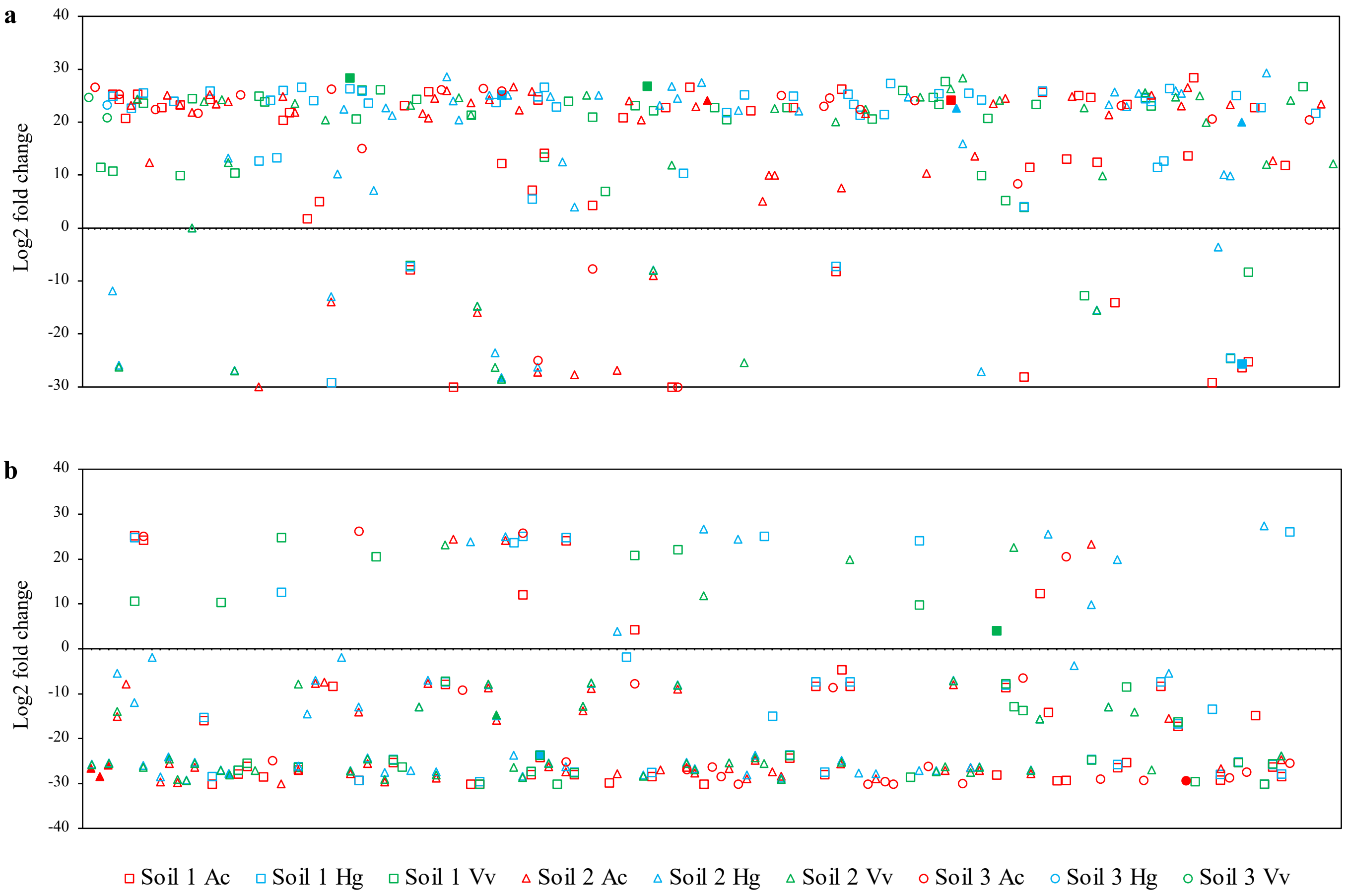

Fig. S26. Chaotic patterns of enriched/depleted ASVs in three soils as some of the enriched/depleted ASVs in one soil showed opposite patterns in other soils. The data shows fold change of the protist enriched (**a**) and depleted (**b**) RE ASVs and their distribution in other treatments. Only significantly enriched/depleted ASVs (*p*<0.01) detected by DESeq based on the **absolute abundances** are shown. Each value was calculated comparing protist treatment with its corresponding control.

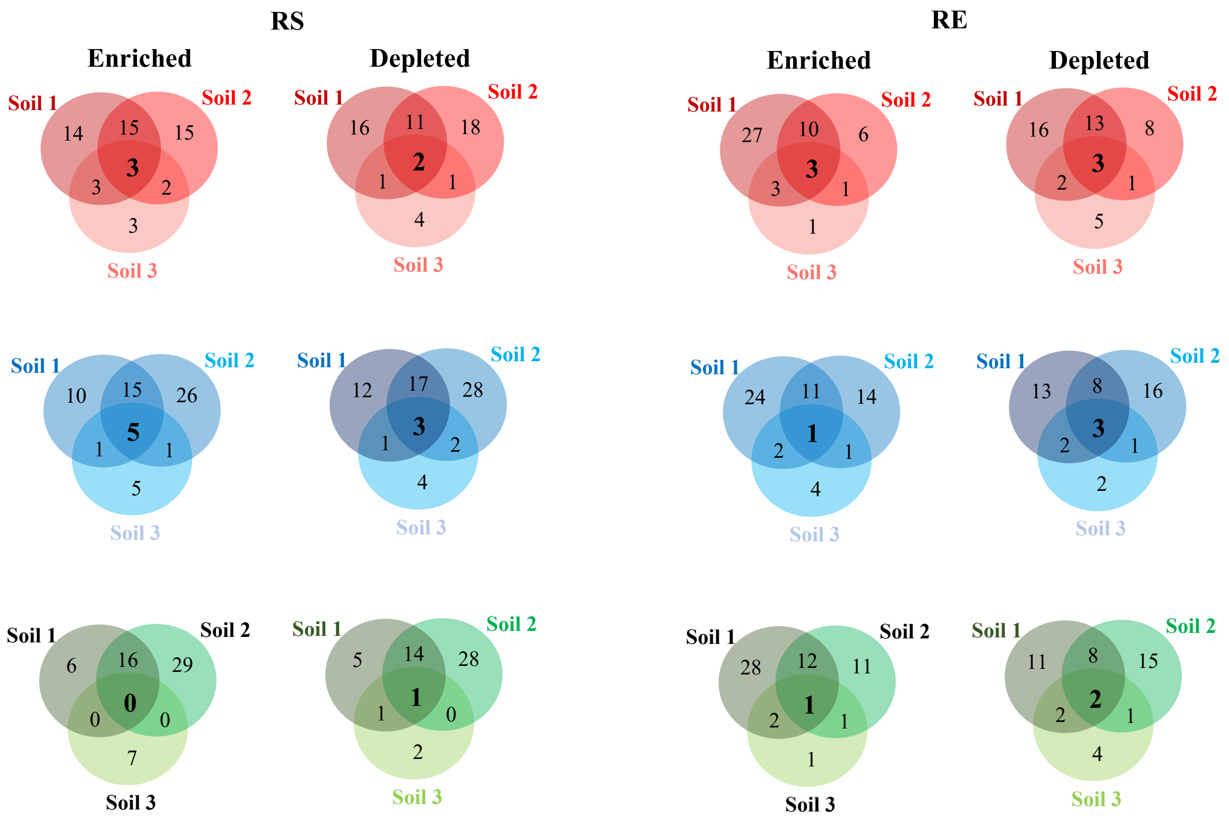

Fig. S27. Venn diagram showing overlap of the protist enriched and depleted RS and RE ASVs (based on the **relative abundances**) grouped at Family level among the 3 soils for each protist. Red, *Acanthamoeba castellanii* (Ac); blue, *Heteromita globosa* (Hg); green, *Vermamoeba vermiformis* (Vv). **b**, the percentage values of unique and shared ASVs.

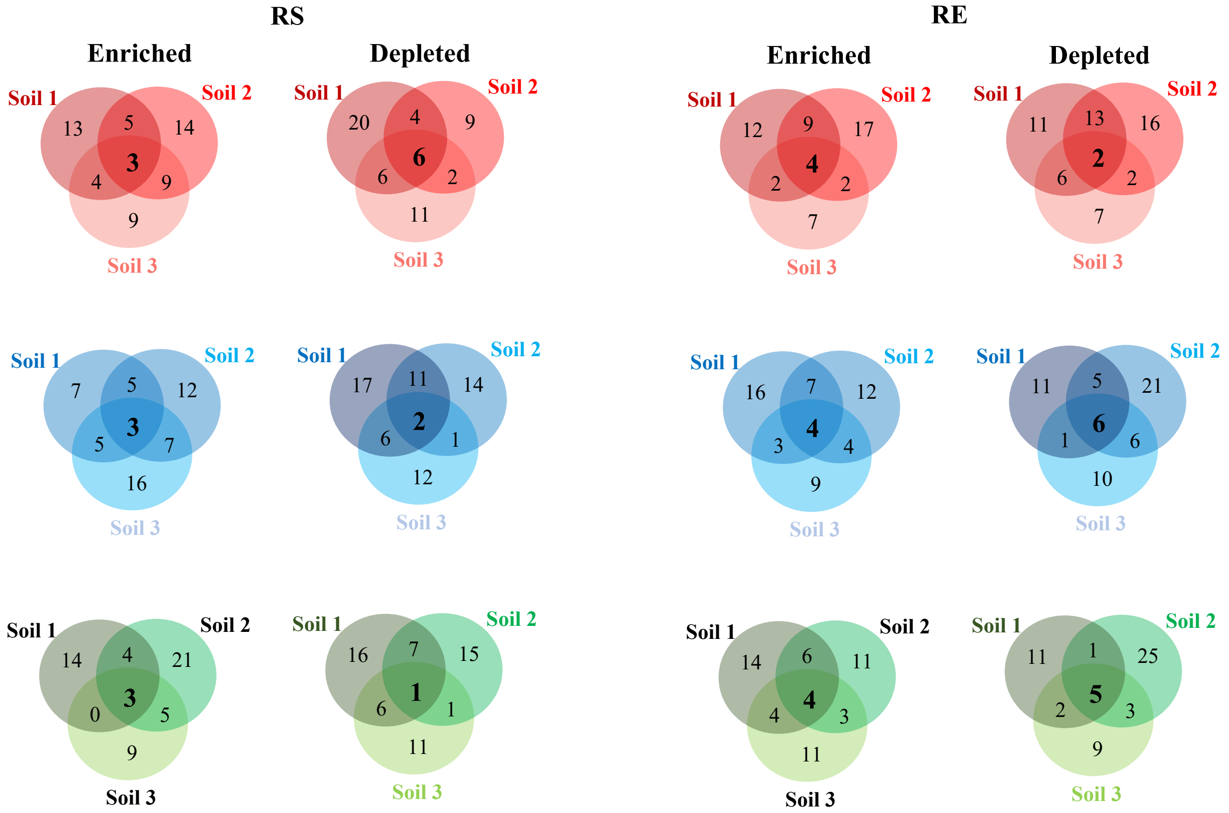

Fig. S28. Venn diagram showing overlap of the protist enriched and depleted RS and RE ASVs (based on the **absolute abundances**) grouped at Family level among the 3 soils for each protist. Red, *Acanthamoeba castellanii* (Ac); blue, *Heteromita globosa* (Hg); green, *Vermamoeba vermiformis* (Vv). **b**, the percentage values of unique and shared ASVs.

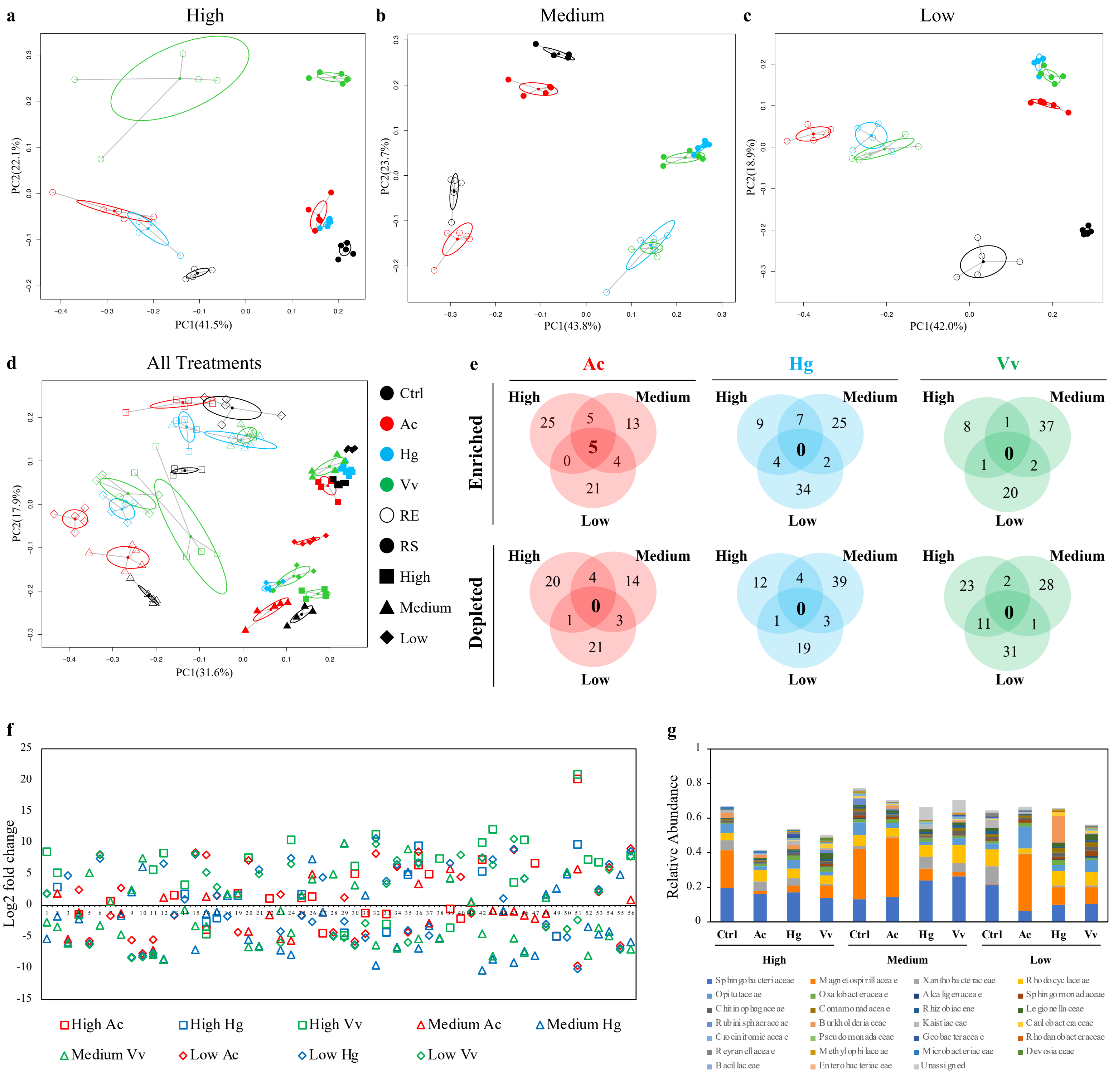

Fig. S29. Trophic regulation of rhizosphere soil (RS) and root endophytic (RE) bacterial communities by protists in three different nutrient levels. **a-d,** Principal Coordinate Analysis (PCoA) based on the Bray–Curtis dissimilarity index of the relative abundances of bacterial communities for dilution rates of ×1.00 (**a**, high,), ×0.75 (**b**, medium,), and ×0.50 (**c**, low) of the nutrient media (Kimura B nutrient solution [pH: 5,8]) showing the effect of protists with confidence ellipses based on the eigenvalues of the covariance matrix (**d**, all treatments together). Black, control without protists (Ctrl); red, *Acanthamoeba castellanii* (Ac); blue, *Heteromita globosa* (Hg); green, *Vermamoeba vermiformis* (Vv); filled circles, rhizosphere soil (RS); empty circles, root endophytes (RE). **e,** Venn diagram showing overlap of the protist enriched and depleted RE ASVs among the 3 dilution rates for each protist. See Fig. S33 for those of RS ASVs. **f**, Fold change of 56 RE ASVs that showed chaotic patterns (enriched or depleted at one dilution rate, each also exhibiting a contrasting trend in at least one of another dilution rates). Each value was calculated comparing each protist treatment with its corresponding control in each dilution rate using DESeq based on relative abundances. **g**, The relative abundances of the 56 RE ASVs that showed chaotic patterns in RE communities grouped at the Family level. See Fig. S35 for the relative abundances of the 56 RE ASVs in RS communities.

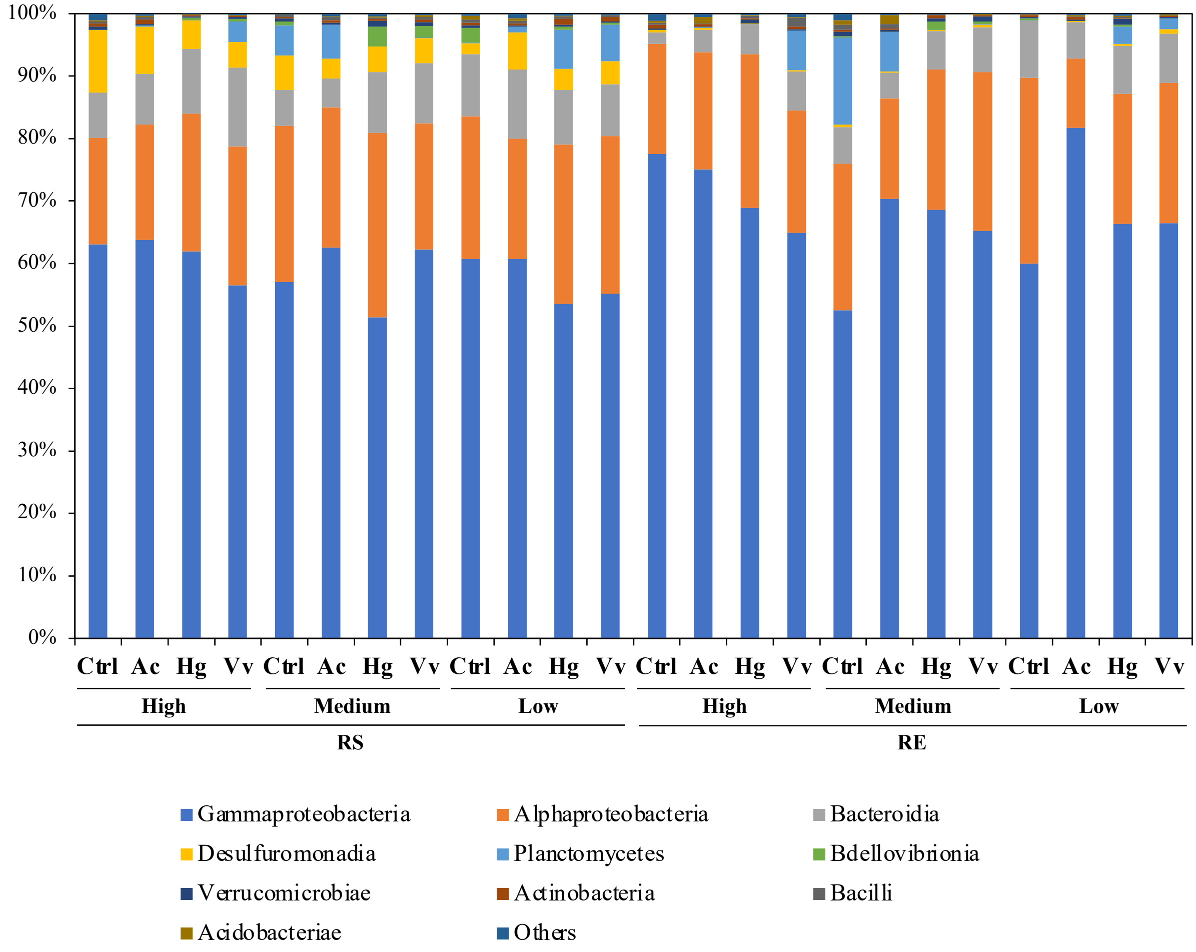

Fig. S30. Taxa barplot representing relative abundance of bacterial taxa at class level (n=5). Ctrl, control without protists; Ac, *Acanthamoeba castellanii*; Hg, *Heteromita globosa*; Vv, *Vermamoeba vermiformis*; High, dilution rate of ×1.00; Medium, dilution rate of ×0.75; Low, dilution rate of ×0.50; RS, rhizosphere soil; RE, root endophytes.

Table S9. PERMANOVA results based on Bray-Curtis dissimilarities calculated from the **relative abundances** of bacterial taxa for the effects of Protists, Nutrient (dilution rates), and Habitat (RS and RE).

Factors	Df	SumOfSqs	R2	F	p values	SF
**Protists**	3	1.1533	0.07913	13.8386	0.001	***
**Habitat**	1	3.7803	0.25936	136.0807	0.001	***
**Nutrient**	2	1.5399	0.10565	27.7155	0.001	***
**Protists:Habitat**	3	0.524	0.03595	6.2876	0.001	***
**Protists:Nutrient**	6	3.6227	0.24855	21.7346	0.001	***
**Habitat:Nutrient**	2	0.453	0.03108	8.1529	0.001	***
**Protists:Habitat:Nutrient**	6	0.9189	0.06305	5.5132	0.001	***
**Residual**	93	2.5835	0.17725			
**Total**	116	14.5757	1			

SF, significant factor (***, <0.001; **, <0.01; *, <0.05)

Table S10. PERMANOVA results based on Bray-Curtis dissimilarities calculated from the **relative abundances** of bacterial taxa for the effects of Protists and Habitat (RS and RE).

Factors	Df	SumOfSqs	R2	F	p values	SF
**Protists**	3	1.2062	0.10887	5.2371	0.001	***
**Habitat**	1	1.363	0.12302	17.7529	0.001	***
**Protists:Habitat**	3	0.2187	0.01974	0.9496	0.535	
**Residual**	108	8.2916	0.74837			
**Total**	115	11.0795	1			

SF, significant factor (***, <0.001; **, <0.01; *, <0.05)

Table S11. PERMANOVA results based on Bray-Curtis dissimilarities calculated from the **relative abundances** of bacterial taxa for the effects of Protists and Nutrients on RE communities.

Factors	Df	SumOfSqs	R2	F	p values	SF
**Protists**	3	1.1038	0.16439	11.22	0.001	***
**Nutrient**	2	1.2482	0.1859	19.032	0.001	***
**Protists:Nutrient**	6	2.8538	0.42504	14.505	0.001	***
**Residual**	46	1.5084	0.22466			
**Total**	57	6.7143	1			

SF, significant factor (***, <0.001; **, <0.01; *, <0.05)

Table S12. PERMANOVA results based on Bray-Curtis dissimilarities calculated from the **relative abundances** of bacterial taxa for the effects of Protists and Nutrients on RS communities.

Factors	Df	SumOfSqs	R2	F	p values	SF
**Protists**	3	0.5542	0.13861	7.9767	0.001	***
**Nutrient**	2	0.7338	0.18353	15.8428	0.001	***
**Protists:Nutrient**	6	1.6449	0.41141	11.8377	0.001	***
**Residual**	46	1.0653	0.26645			
**Total**	57	3.9982	1			

SF, significant factor (***, <0.001; **, <0.01; *, <0.05)

Table S13. PERMANOVA results based on Bray-Curtis dissimilarities calculated from the log transformed **absolute abundances** of bacterial taxa for the effects of Protists, Nutrient (dilution rates), and Habitat (RS and RE).

Factors	Df	SumOfSqs	R2	F	p values	SF
**Protists**	3	1.4162	0.09056	10.7711	0.001	***
**Habitat**	1	3.8469	0.24599	87.7708	0.001	***
**Nutrient**	2	1.5574	0.09959	17.7669	0.001	***
**Protists:Habitat**	3	0.3105	0.01986	2.3616	0.001	***
**Protists:Nutrient**	6	3.4575	0.2211	13.1479	0.001	***
**Habitat:Nutrient**	2	0.3649	0.02334	4.163	0.001	***
**Protists:Habitat:Nutrient**	6	0.6524	0.04172	2.4808	0.001	***
**Residual**	92	4.0322	0.25785			
**Total**	115	15.6381	1			

SF, significant factor (***, <0.001; **, <0.01; *, <0.05)

Table S14. PERMANOVA results based on Bray-Curtis dissimilarities calculated from the log transformed **absolute abundances** of bacterial taxa for the effects of Protists and Habitat (RS and RE).

Factors	Df	SumOfSqs	R2	F	p values	SF
**Protists**	3	1.4162	0.09056	5.0671	0.001	***
**Habitat**	1	3.8469	0.24599	41.2904	0.001	***
**Protists:Habitat**	3	0.313	0.02002	1.12	0.247	
**Residual**	108	10.062	0.64343			
**Total**	115	15.6381	1			

SF, significant factor (***, <0.001; **, <0.01; *, <0.05)

Table S15. PERMANOVA results based on Bray-Curtis dissimilarities calculated from the log transformed **absolute abundances** of bacterial taxa for the effects of Protists and Nutrients on RE communities.

Factors	Df	SumOfSqs	R2	F	p values	SF
**Protists**	3	0.8458	0.14443	6.4014	0.001	***
**Nutrient**	2	0.983	0.16786	11.16	0.001	***
**Protists:Nutrient**	6	2.0013	0.34175	7.5736	0.001	***
**Residual**	46	2.0258	0.34595			
**Total**	57	5.8558	1			

SF, significant factor (***, <0.001; **, <0.01; *, <0.05)

Table S16. PERMANOVA results based on Bray-Curtis dissimilarities calculated from the log transformed **absolute abundances** of bacterial taxa for the effects of Protists and Nutrients on RS communities.

Factors	Df	SumOfSqs	R2	F	p values	SF
**Protists**	3	0.8835	0.14886	6.7521	0.001	***
**Nutrient**	2	0.9434	0.15894	10.8143	0.001	***
**Protists:Nutrient**	6	2.1021	0.35417	8.0325	0.001	***
**Residual**	46	2.0064	0.33804			
**Total**	57	5.9354	1			

SF, significant factor (***, <0.001; **, <0.01; *, <0.05)

a

RS			RE High Ac	RE High Hg	RE High Vv	RE Medium Ac	RE Medium Hg	RE Medium Vv	RE Low Ac	RE Low Hg	RE Low Vv
RS	High	Ac	16	5	8	5	7	8	3	6	6
		Hg	9	12	4	4	5	8	2	2	1
		Vv	8	3	21	4	2	3	9	14	13
	Medium	Ac	5	3	5	10	2	2	7	6	5
		Hg	10	6	3	7	18	14	5	6	5
		Vv	7	6	4	3	13	19	2	3	2
	Low	Ac	3	2	8	5	3	2	16	15	13
		Hg	3	2	10	5	1	1	11	21	19
		Vv	5	3	11	6	3	2	6	21	21

b

RS			RE High Ac	RE High Hg	RE High Vv	RE Medium Ac	RE Medium Hg	RE Medium Vv	RE Low Ac	RE Low Hg	RE Low Vv
RS	High	Ac	10	7	2	5	5	3	4	4	4
		Hg	9	7	3	3	4	2	5	5	5
		Vv	5	7	11	3	3	1	9	7	6
	Medium	Ac	3	3	0	12	14	12	3	3	4
		Hg	6	4	1	15	32	27	1	3	2
		Vv	2	2	0	12	26	24	1	3	2
	Low	Ac	2	0	0	5	3	1	21	16	16
		Hg	1	1	1	4	3	2	10	12	11
		Vv	1	1	1	4	3	2	12	13	12

Fig. S31. Matrix showing the total numbers of shared protist enriched (**a**) and depleted (**b**) ASVs detected by DESeq based on the **relative abundances** between RS and RE treatments. The gradient green and red color indicates the higher numbers in each row for enriched and depleted ASVs, respectively.

a

		RE								
		High			Medium			Low		
RS		Ac	Hg	Vv	Ac	Hg	Vv	Ac	Hg	Vv
High	Ac	11	10	8	6	4	5	3	6	7
	Hg	8	12	10	6	4	3	3	4	3
	Vv	6	6	15	4	1	0	4	8	9
Medium	Ac	5	7	7	14	6	5	5	9	6
	Hg	0	2	2	4	6	5	2	2	0
	Vv	2	2	7	6	5	7	0	4	0
Low	Ac	5	5	8	8	2	3	11	12	10
	Hg	5	5	5	11	4	3	8	16	16
	Vv	4	4	7	9	3	2	6	15	15

b

		RE								
		High			Medium			Low		
RS		Ac	Hg	Vv	Ac	Hg	Vv	Ac	Hg	Vv
High	Ac	6	5	2	3	1	1	1	1	1
	Hg	6	9	7	2	2	3	2	2	1
	Vv	6	6	8	2	2	3	4	3	1
Medium	Ac	0	1	1	6	4	5	4	2	4
	Hg	2	3	2	4	11	10	2	3	2
	Vv	2	4	2	2	9	9	3	3	2
Low	Ac	2	1	2	2	1	1	7	5	6
	Hg	1	1	2	1	1	1	6	7	6
	Vv	4	4	4	2	2	2	5	5	7

Fig. S32. Matrix showing the total numbers of shared protist enriched (**a**) and depleted (**b**) ASVs detected by DESeq based on the **absolute abundances** between RS and RE treatments. The gradient green and red color indicates the higher numbers in each row for enriched and depleted ASVs, respectively.

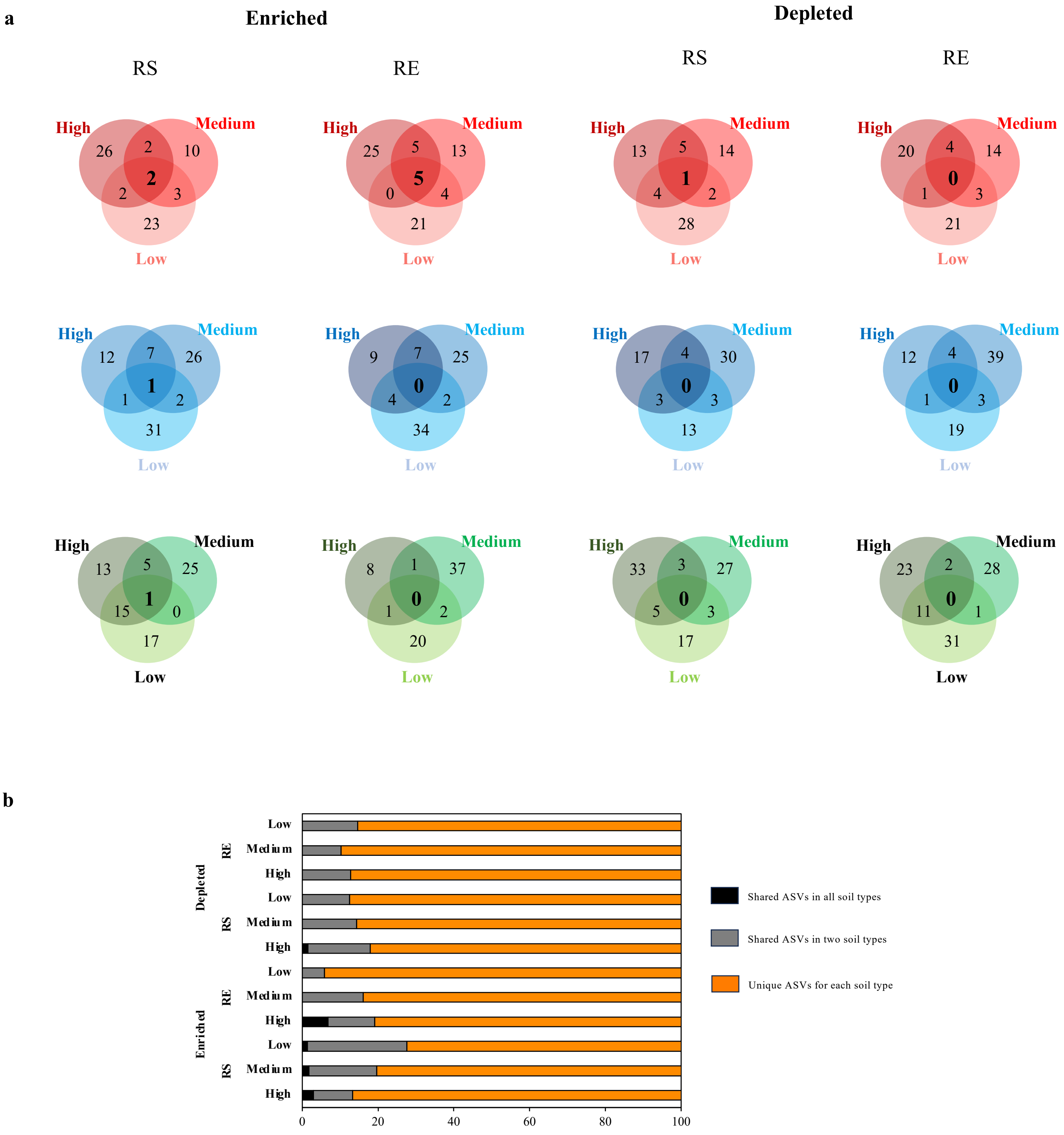

Fig. S33. a, Venn diagram showing overlap of the protist enriched and depleted RS and RE ASVs detected by DESeq based on the **relative abundances** among the dilution rates for each protist. Red, *Acanthamoeba castellanii* (Ac); blue, *Heteromita globosa* (Hg); green, *Vermamoeba vermiformis* (Vv). **b**, the percentage values of unique and shared ASVs.

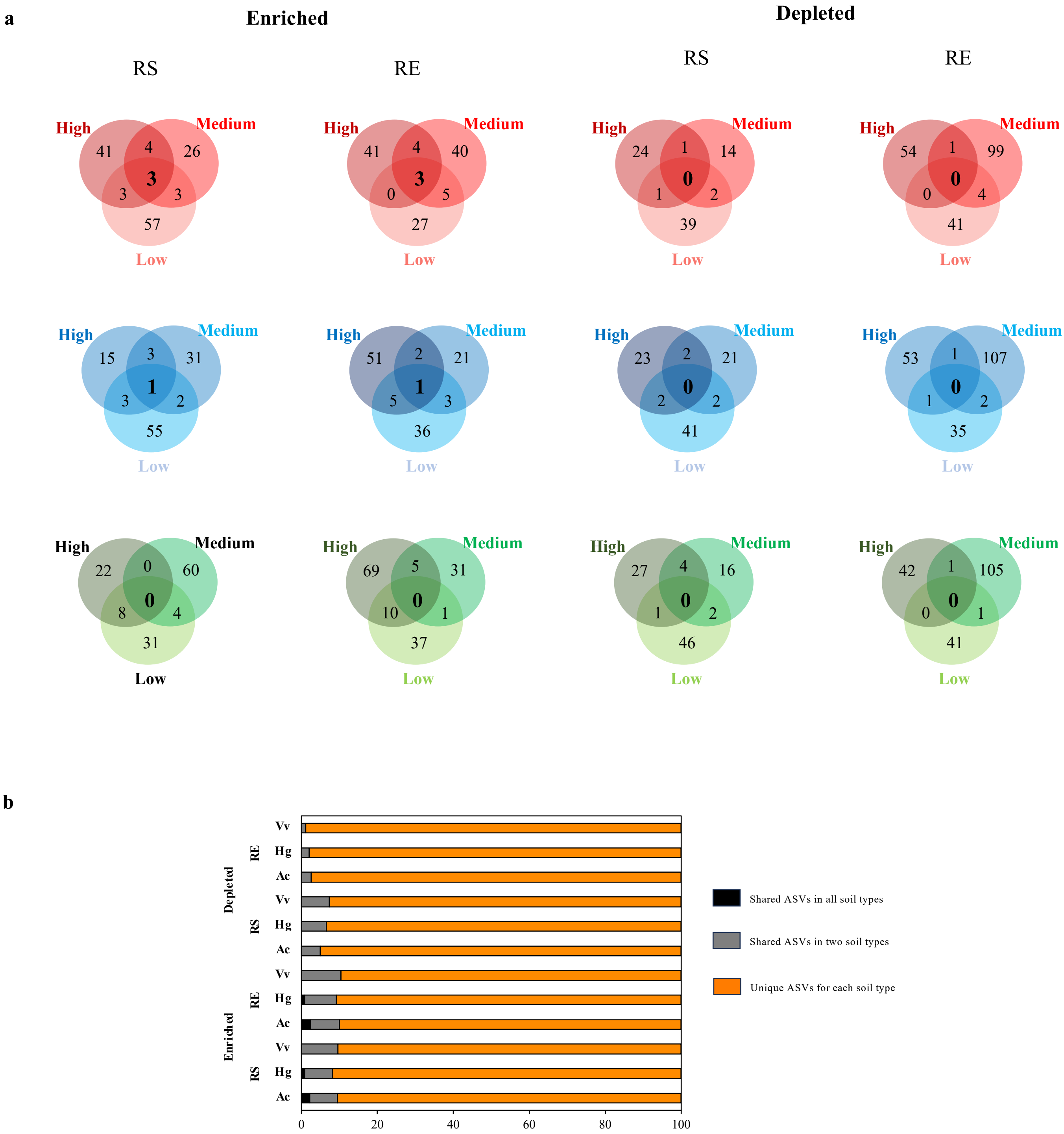

Fig. S34. a, Venn diagram showing overlap of the protist enriched and depleted RS and RE ASVs detected by DESeq based on the **absolute abundances** among the dilution rates for each protist. Red, *Acanthamoeba castellanii* (Ac); blue, *Heteromita globosa* (Hg); green, *Vermamoeba vermiformis* (Vv). **b**, the percentage values of unique and shared ASVs.

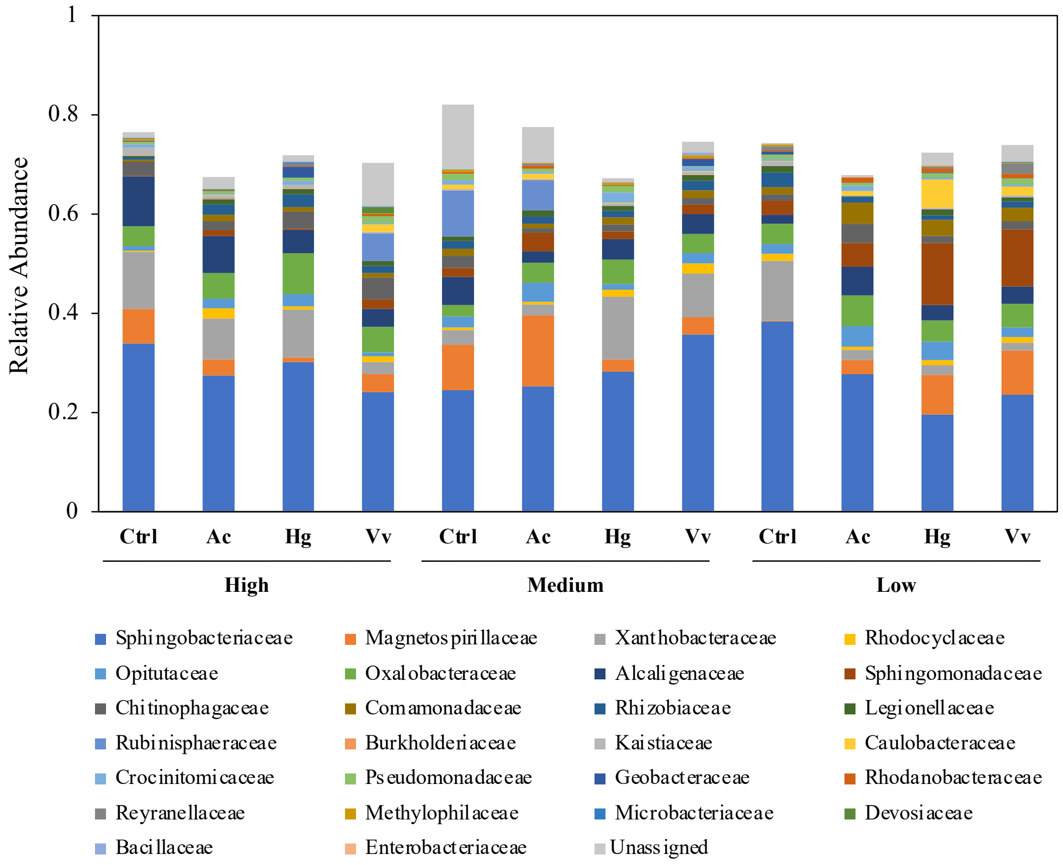

Fig. S35. The relative abundances of the 56 RE ASVs that showed chaotic patterns in RS communities grouped at the Family level.

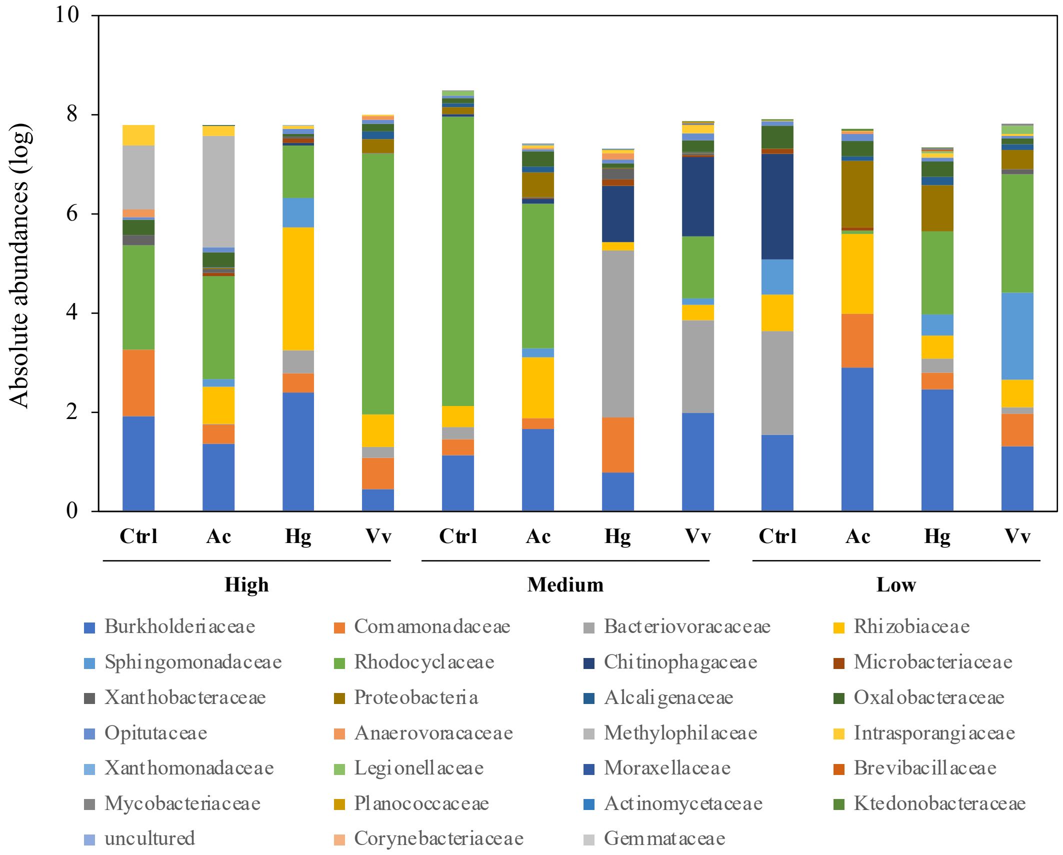

Fig. S36. The relative abundances of the 46 RE ASVs that showed chaotic patterns in RS communities grouped at the Family level. Colors indicate relative distribution of each family.

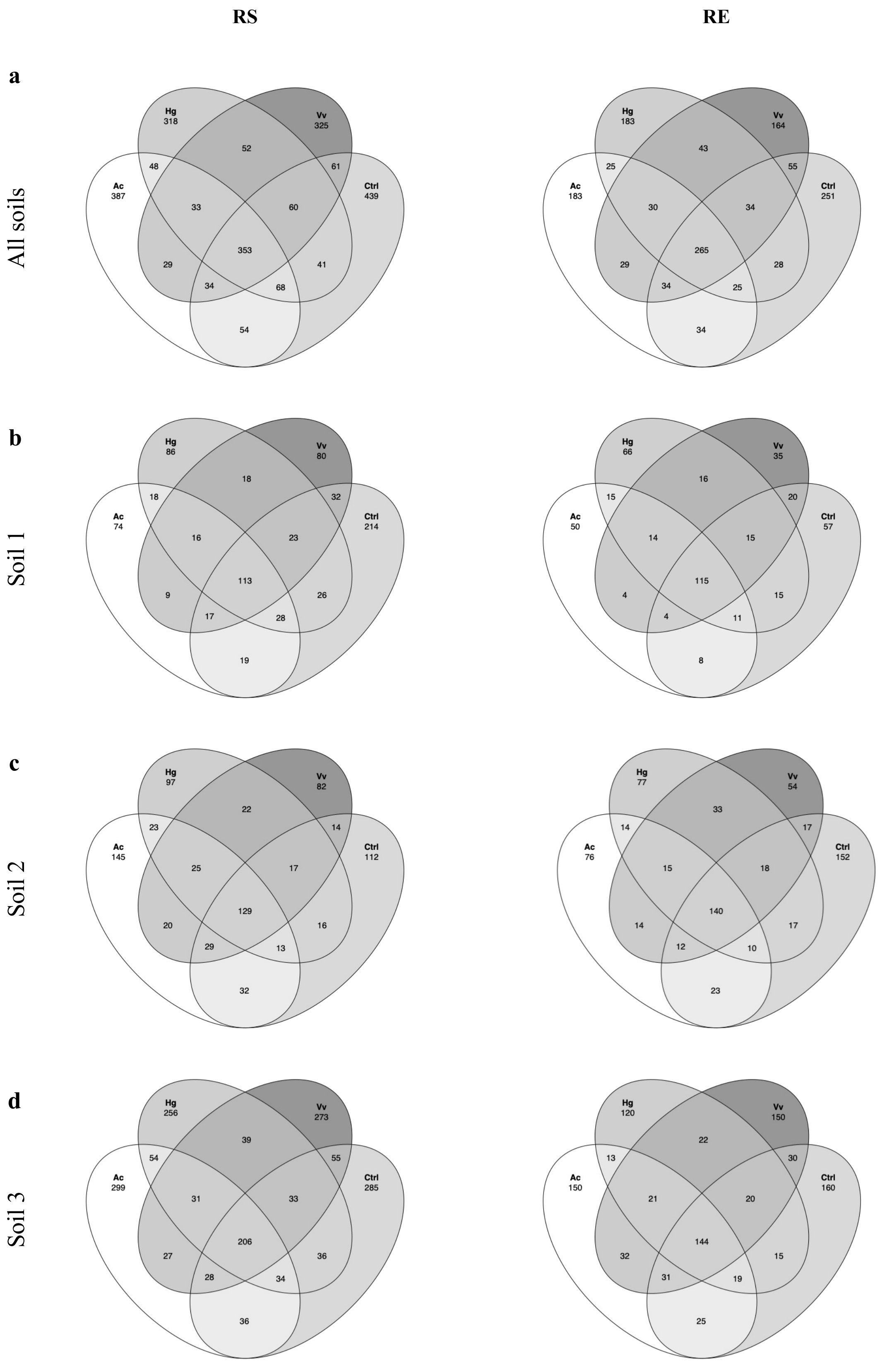

Fig. S37. Venn diagram showing overlap of the ASVs among the protist treatments in rhizosphere soil (RS) and roots (RE) in all soils (**a**), Soil 1 (**b**), Soil 2 (**c**), and Soil 3 (**d**). Numbers indicate total ASVs.

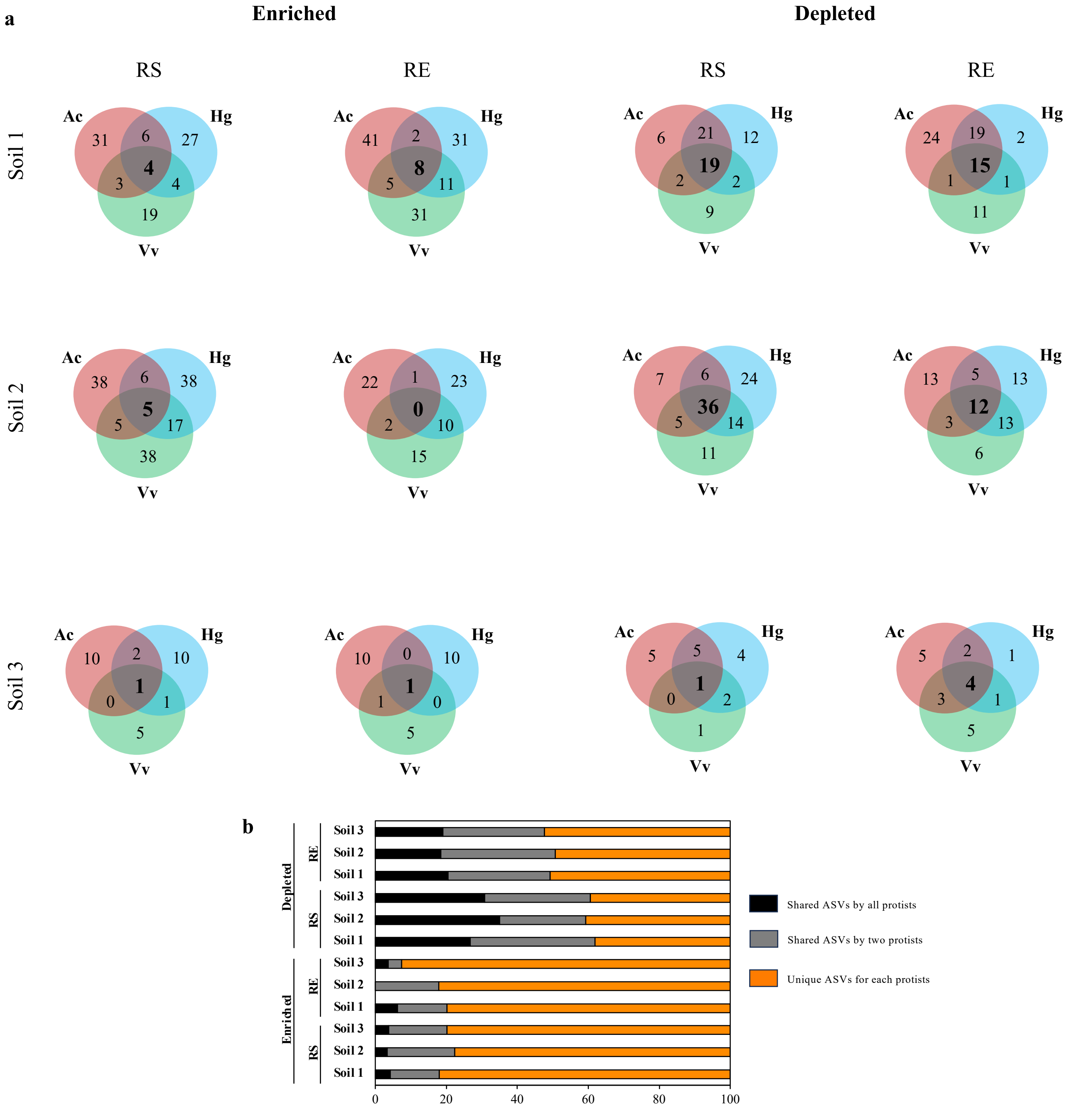

Fig. S38. a, Venn diagram showing overlap of the protist enriched and depleted RS and RE ASVs detected by DESeq based on the **relative abundances** among the 3 protists for each soil. Red, *Acanthamoeba castellanii* (Ac); blue, *Heteromita globosa* (Hg); green, *Vermamoeba vermiformis* (Vv). **b**, the percentage values of unique and shared ASVs.

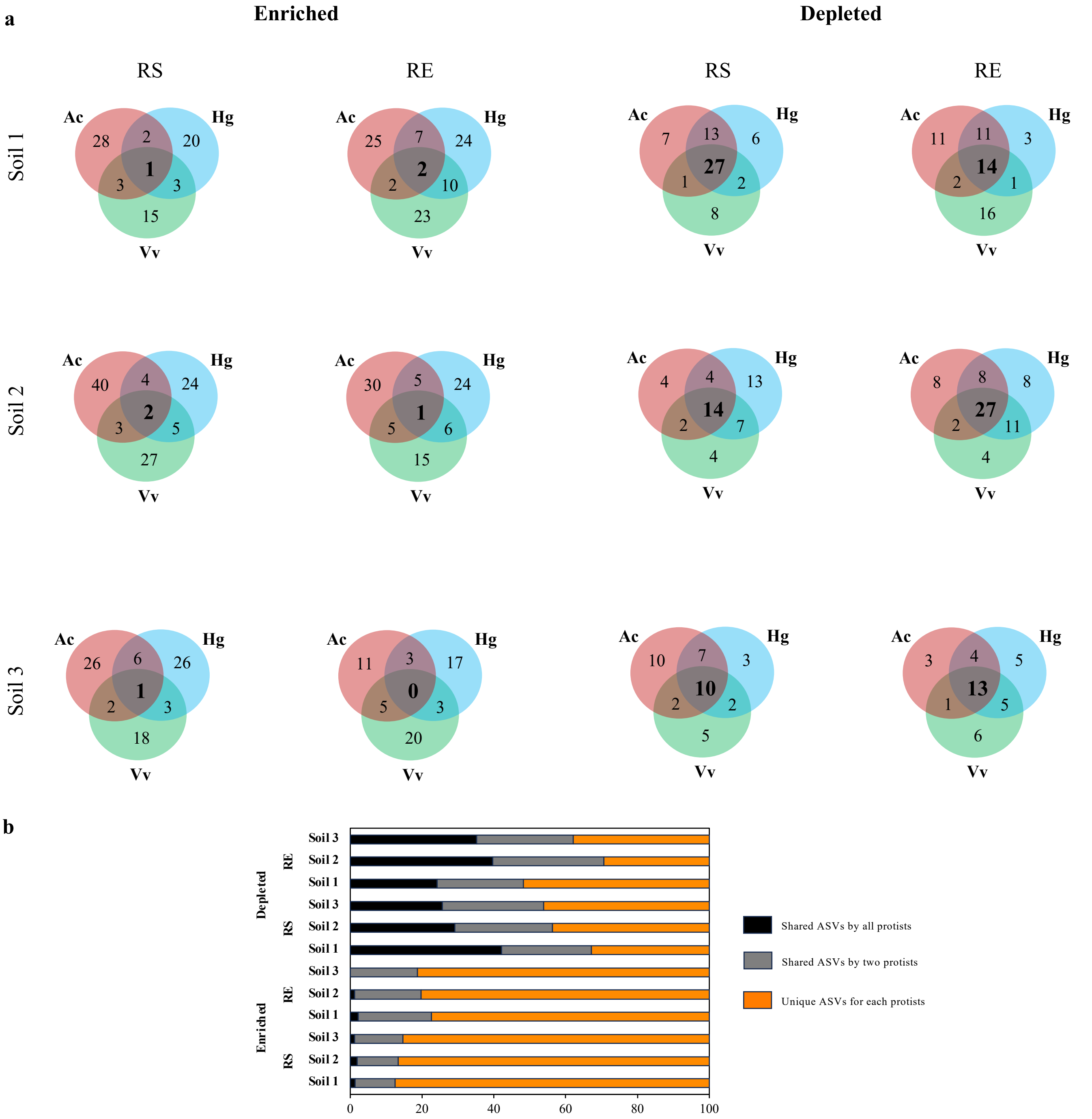

Fig. S39. a, Venn diagram showing overlap of the protist enriched and depleted RS and RE ASVs detected by DESeq based on the **absolute abundances** among the 3 protists for each soil. Red, *Acanthamoeba castellanii* (Ac); blue, *Heteromita globosa* (Hg); green, *Vermamoeba vermiformis* (Vv). **b**, the percentage values of unique and shared ASVs.

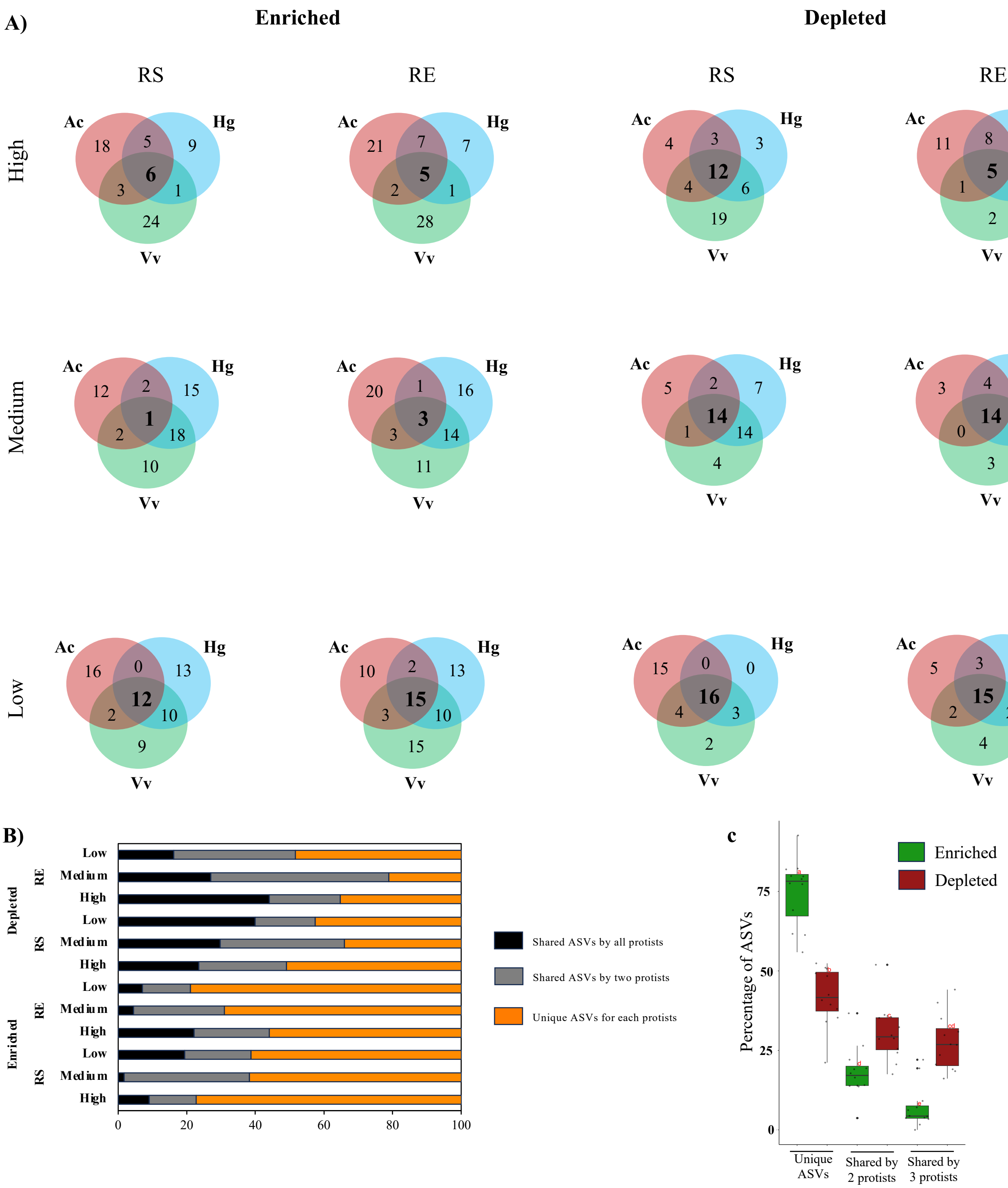

Fig. S40. a, Venn diagram showing overlap of the protist enriched and depleted RS and RE ASVs detected by DESeq based on the **relative abundances** among the 3 protists for each dilution rate. Red, *Acanthamoeba castellanii* (Ac); blue, *Heteromita globosa* (Hg); green, *Vermamoeba vermiformis* (Vv). **b**, the percentage values of unique and shared ASVs. **c**, Box plot representing within group variance of unique and shared ASVs among the 3 protist treatments. Dark green, enriched ASVs, dark red, depleted ASVs. The data is represents all treatments from both Experiment 1 and 2. The central line in the boxplot represents the median, box hinges represent first and third quartiles. Lines indicate minimum and maximum values. Different letters represent significant differences (p<0.05, ANOVA with Tukey's post hoc test).

A)

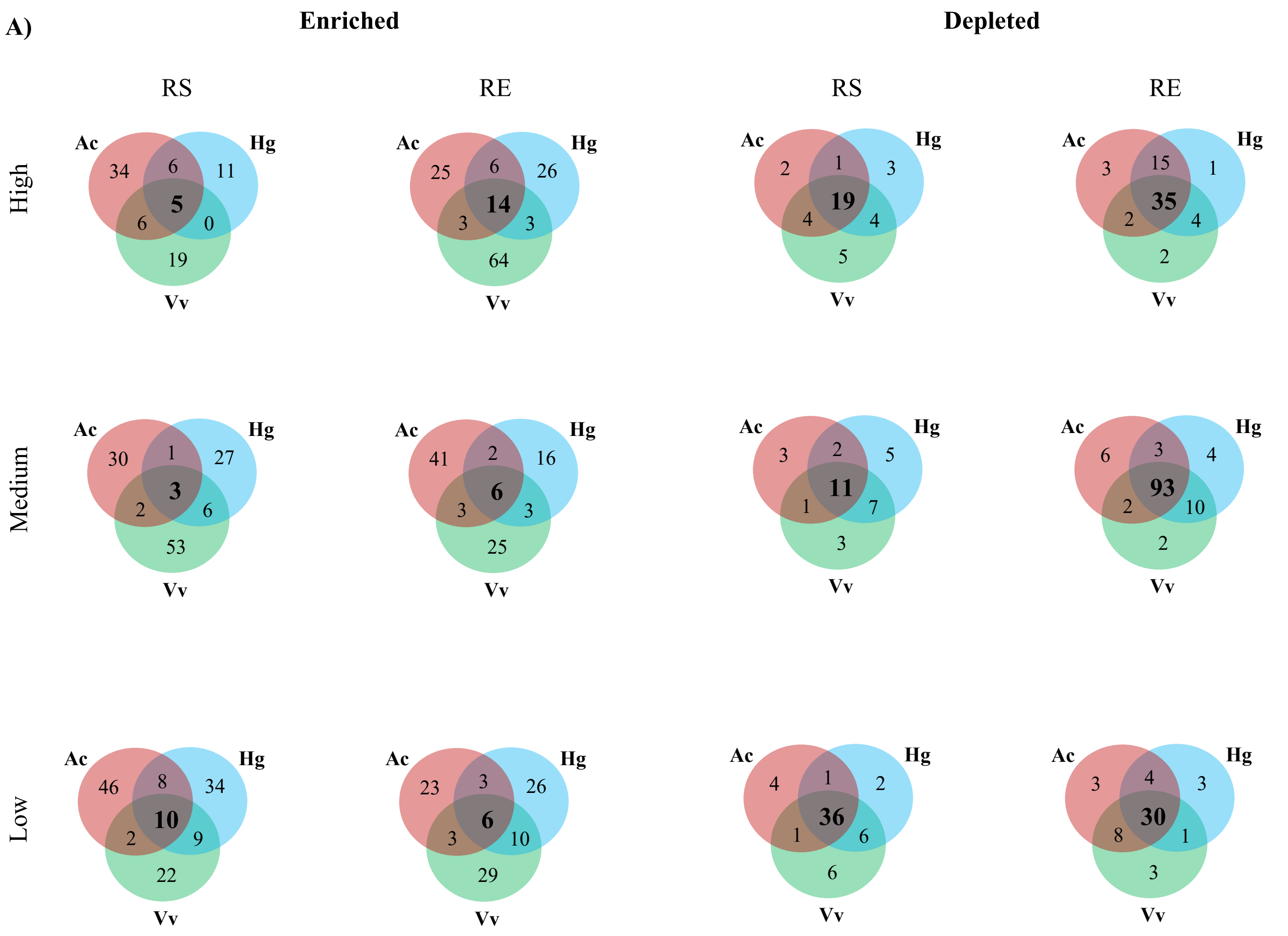

B)

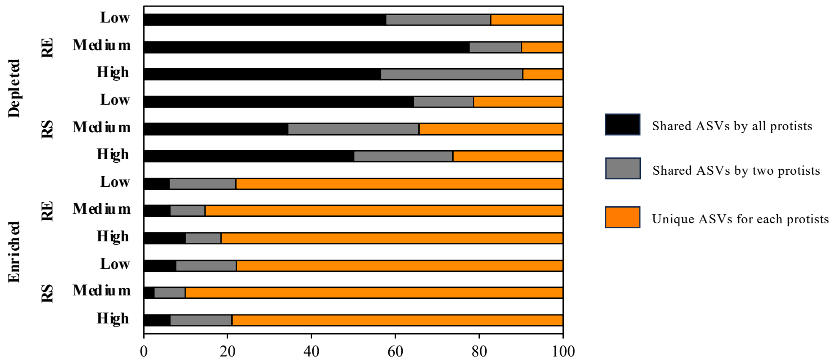

Fig. S41. a, Venn diagram showing overlap of the protist enriched and depleted RS and RE ASVs detected by DESeq based on the **absolute abundances** among the 3 protists for each dilution rate. Red, *Acanthamoeba castellanii* (Ac); blue, *Heteromita globosa* (Hg); green, *Vermamoeba vermiformis* (Vv). **b**, the percentage values of unique and shared ASVs.

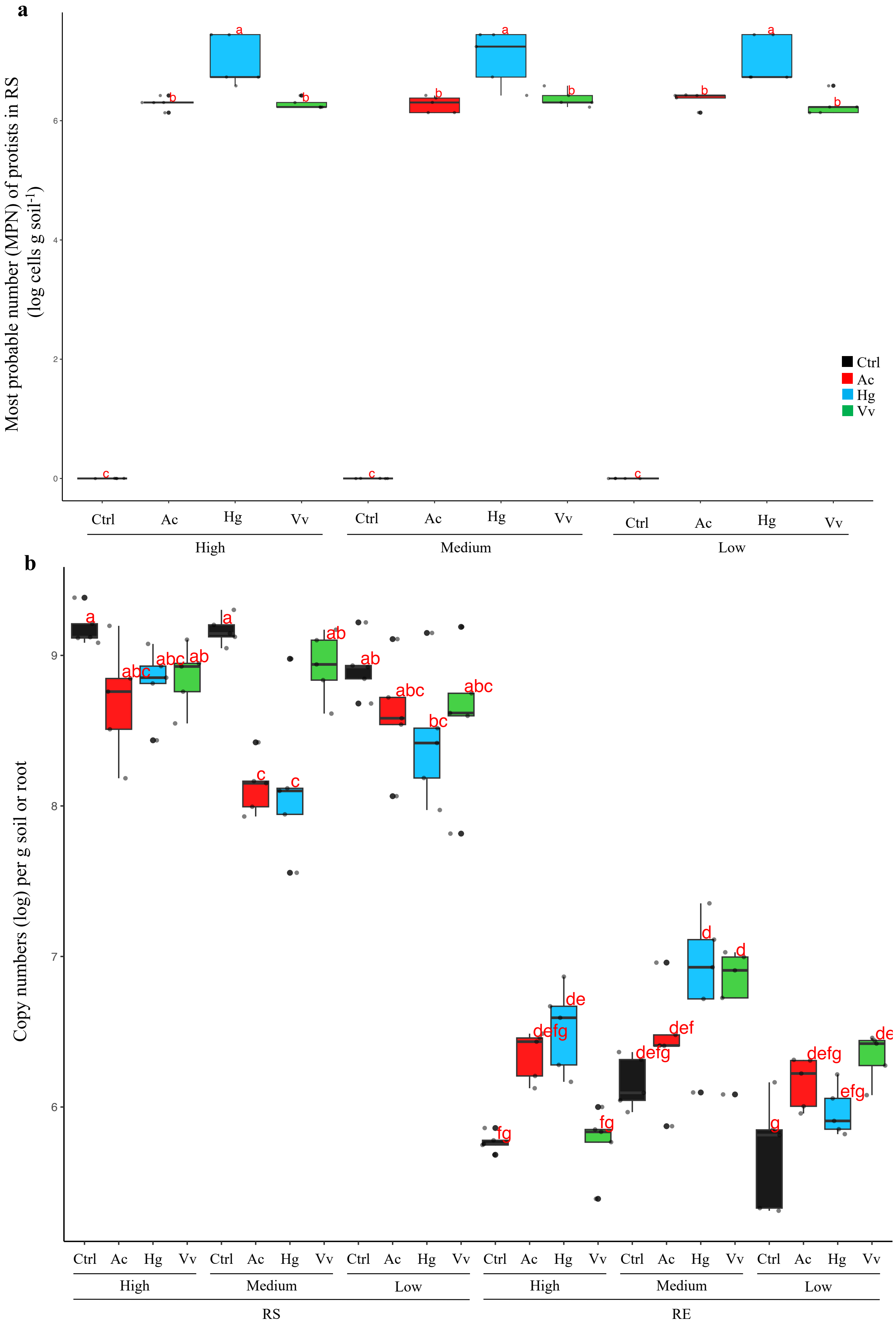

Fig. S42. Box plot representing within group variance of number of protists (**a**) and 16S rRNA gene copy numbers (**b**). The central line in the boxplot represents the median, box hinges represent first and third quartiles. Lines indicate minimum and maximum values. Different letters represent significant differences (p<0.05, ANOVA with Tukey's post hoc test). Black, control without protists (Ctrl); red, *Acanthamoeba castellanii* (Ac); blue, *Heteromita globosa* (Hg); green, *Vermamoeba vermiformis* (Vv); RS, rhizosphere soil; RE, root endophytes.

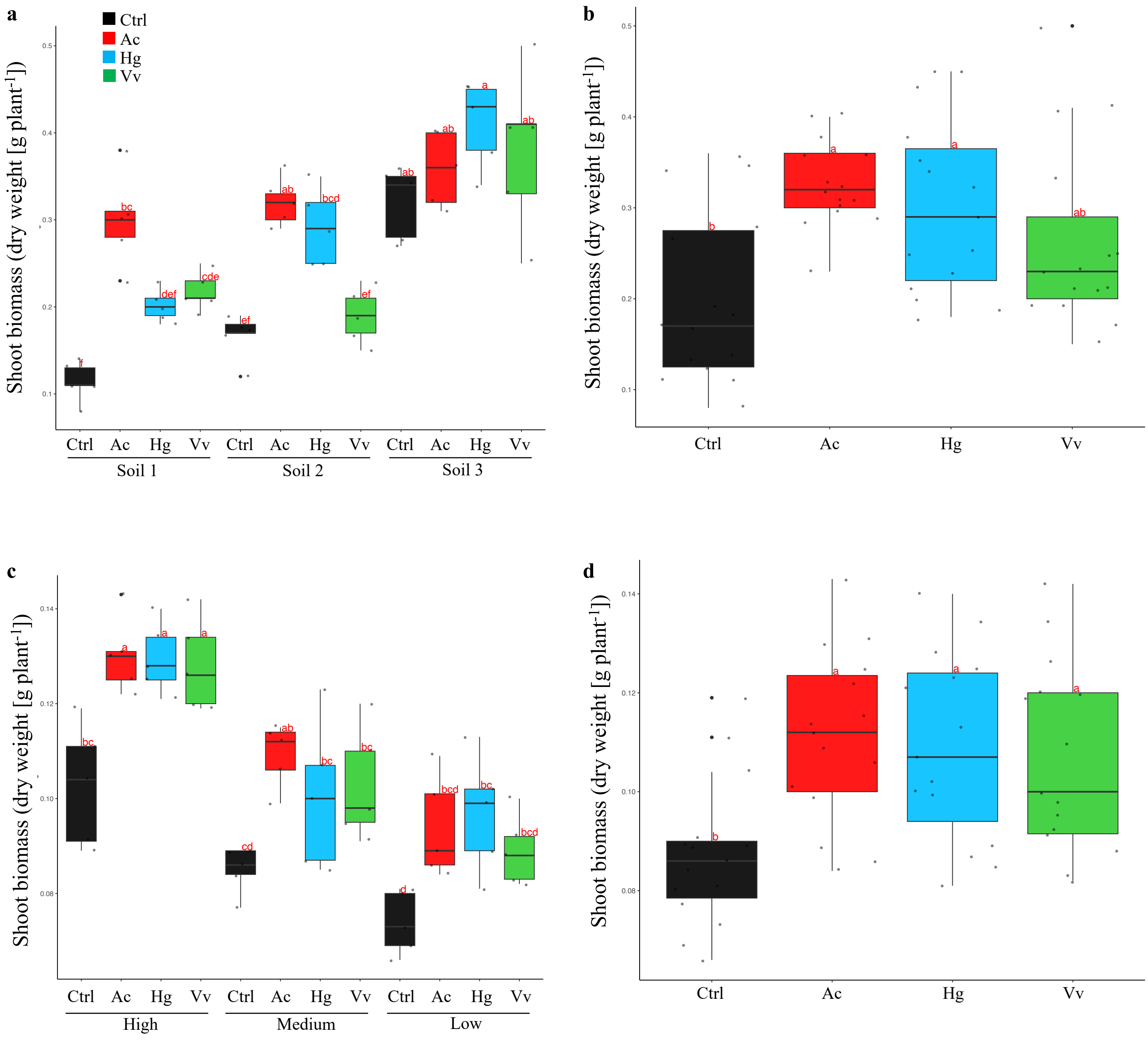

Fig. S43. Box plot representing within group variance of plant biomass in Experiment 1 (**a-b**) and 2 (**c-d**) for each soil/dilution rate (**a,c**) and for each protist in all soils/dilution rates (**b,d**). The central line in the boxplot represents the median, box hinges represent first and third quartiles. Lines indicate minimum and maximum values. Different letters represent significant differences (p<0.05, ANOVA with Tukey's post hoc test). Black, control without protists (Ctrl); red, *Acanthamoeba castellanii* (Ac); blue, *Heteromita globosa* (Hg); green, *Vermamoeba vermiformis* (Vv); RS, rhizosphere soil; RE, root endophytes.

#CODES

#Qiime2 CODES

```
conda activate qiime2-2021.11

#Soil 1, Ach or ACH; Soil 2, Shd or SHD; Soil 3, Ngn or NGN.

#----------------------------------------------------------

qiime tools import \
  --type 'SampleData[PairedEndSequencesWithQuality]' \
  --input-path Data \
  --input-format CasavaOneEightSingleLanePerSampleDirFmt \
  --output-path demux-paired-end.qza


#----------------------------------------------------------

qiime demux summarize \
  --i-data demux-paired-end.qza \
  --o-visualization demux.qzv


#----------------------------------------------------------

qiime tools view demux.qzv


#----------------------------------------------------------

mv demux-paired-end.qza demux.qza


#----------------------------------------------------------

qiime dada2 denoise-paired \
  --i-demultiplexed-seqs demux.qza \
  --p-trim-left-f 17 \
  --p-trim-left-r 21 \
  --p-trunc-len-f 210 \
```

```
  --p-trunc-len-r 140 \
  --o-representative-sequences rep-seqs.qza \
  --o-table table.qza \
  --o-denoising-stats denoising_stats.qza
  --p-n-reads-learn 10000 \
  --p-n-threads 0 \
  --verbose


#-----------------------------------------------------------

qiime feature-table summarize \
  --i-table table.qza \
  --o-visualization table.qzv \
  --m-sample-metadata-file metadata.tsv

qiime feature-table tabulate-seqs \
  --i-data rep-seqs.qza \
  --o-visualization rep-seqs.qzv

qiime metadata tabulate \
  --m-input-file denoising_stats.qza \
  --o-visualization stats-dada2.qzv

#-----------------------------------------------------------

qiime feature-table filter-features \
  --i-table filtered_table.qza \
  --p-min-frequency 2 \
  --o-filtered-table feature-frequency-filtered-table.qza

qiime feature-table filter-seqs \
  --i-data filtered-seq.qza \
  --i-table feature-frequency-filtered-table.qza \
  --o-filtered-data feature-frequency-filtered-rep-seqs.qza \
  --verbose

#-----------------------------------------------------------

qiime feature-table summarize \
  --i-table feature-frequency-filtered-table.qza \
  --o-visualization feature-frequency-filtered-table.qzv \
  --m-sample-metadata-file metadata.tsv
```

```
qiime feature-table tabulate-seqs \
  --i-data feature-frequency-filtered-rep-seqs.qza \
  --o-visualization feature-frequency-filtered-rep-seqs.qzv

#-----------------------------------------------------------


qiime feature-classifier classify-sklearn \
  --i-classifier 515f-806r-average-classifier.qza \
  --i-reads filtered-seq.qza \
  --p-n-jobs -1 \
  --o-classification taxonomy.qza \
  --verbose



qiime taxa barplot \
  --i-table feature-frequency-filtered-table.qza \
  --i-taxonomy taxonomy.qza \
  --m-metadata-file metadata.tsv \
  --o-visualization taxa-bar-plots.qzv

#-----------------------------------------------------------

qiime taxa filter-table \
  --i-table feature-frequency-filtered-table.qza \
  --i-taxonomy taxonomy.qza \
  --p-include p__ \
  --p-exclude Unassigned,Archaea,Mitochondria,Chloroplast  \
  --output-dir filtered



qiime feature-table summarize \
  --i-table filtered/filtered_table.qza \
  --o-visualization filtered/filtered-table.qzv \
  --m-sample-metadata-file metadata.tsv

qiime taxa barplot \
  --i-table filtered/filtered_table.qza \
  --i-taxonomy taxonomy.qza \
  --m-metadata-file metadata.tsv \
  --o-visualization filtered/taxa-bar-plots-filtered.qzv
```

```
qiime taxa filter-seqs \
  --i-sequences feature-frequency-filtered-rep-seqs.qza \
  --i-taxonomy taxonomy.qza \
  --p-include p__ \
  --p-exclude Unassigned,Archaea,Mitochondria,Chloroplast \
  --o-filtered-sequences filtered/filtered-seq.qza

#---------------------------------------------------------


qiime phylogeny align-to-tree-mafft-fasttree \
  --i-sequences feature-frequency-filtered-rep-seqs.qza \
  --p-n-threads auto \
  --o-alignment aligned-rep-seqs.qza \
  --o-masked-alignment masked-aligned-rep-seqs.qza \
  --o-tree unrooted-tree.qza \
  --o-rooted-tree rooted-tree.qza \
  --verbose


qiime diversity core-metrics-phylogenetic \
  --i-phylogeny rooted-tree.qza \
  --i-table feature-frequency-filtered-table.qza \
  --p-sampling-depth 10000 \
  --m-metadata-file metadata.tsv \
  --output-dir core-metrics-results


#---------------------------------------------------------

qiime diversity alpha-group-significance \
  --i-alpha-diversity core-metrics-results/faith_pd_vector.qza \
  --m-metadata-file metadata.tsv \
  --o-visualization core-metrics-results/faith-pd-group-significance.qzv

qiime diversity alpha-group-significance \
  --i-alpha-diversity core-metrics-results/evenness_vector.qza \
  --m-metadata-file metadata.tsv \
  --o-visualization core-metrics-results/evenness-group-significance.qzv


qiime tools export \
  --input-path filtered/core-metrics-results/bray_curtis_distance_matrix.qza \
```

```
  --output-path filtered/core-metrics-results/export/bray_curtis_distance_matrix

qiime tools export \
  --input-path filtered/core-metrics-results/bray_curtis_pcoa_results.qza \
  --output-path filtered/core-metrics-results/export/bray_curtis_pcoa_results


#----------------------------------------------------------


qiime tools export --input-path core-metrics-results/shannon_vector.qza --output-path core-metrics-results
mv core-metrics-results/alpha-diversity.tsv filtered/core-metrics-results/shannon.tsv

qiime tools export --input-path core-metrics-results/observed_features_vector.qza --output-path core-metrics-results
mv core-metrics-results/alpha-diversity.tsv core-metrics-results/observed_features.tsv

qiime tools export --input-path filtered/core-metrics-results/faith_pd_vector.qza --output-path filtered/core-metrics-results
mv filtered/core-metrics-results/alpha-diversity.tsv filtered/core-metrics-results/faith_pd.tsv

qiime tools export --input-path filtered/core-metrics-results/evenness_vector.qza --output-path filtered/core-metrics-results
mv filtered/core-metrics-results/alpha-diversity.tsv filtered/core-metrics-results/evenness.tsv


#----------------------------------------------------------

qiime taxa barplot \
  --i-table filtered/core-metrics-results/rarefied_table.qza \
  --i-taxonomy taxonomy.qza \
  --m-metadata-file metadata.tsv \
  --o-visualization filtered/core-metrics-results/taxa-bar-plots-rarefied.qzv

#----------------------------------------------------------

qiime feature-table filter-samples \
  --i-table filtered/core-metrics-results/rarefied_table.qza \
  --m-metadata-file metadata.tsv \
  --p-where "[SoilType]='Shd'" \
  --o-filtered-table filtered/core-metrics-results/rarefied_Shd_table.qza
```

```
qiime diversity core-metrics-phylogenetic \
  --i-phylogeny filtered/rooted-tree.qza \
  --i-table filtered/core-metrics-results/rarefied_Shd_table.qza \
  --p-sampling-depth 10000 \
  --m-metadata-file metadata.tsv \
  --output-dir filtered/core-metrics-results/Shd-core-metrics



qiime tools export \
  --input-path filtered/core-metrics-results/shd-core-metrics/bray_curtis_distance_matrix.qza \
  --output-path shdfiltered/core-metrics-results/export/bray_curtis_distance_matrix

qiime tools export \
  --input-path filtered/core-metrics-results/shd-core-metrics/bray_curtis_pcoa_results.qza \
  --output-path shdfiltered/core-metrics-results/export/bray_curtis_pcoa_results

qiime tools export \
  --input-path filtered/core-metrics-results/ach-core-metrics/bray_curtis_distance_matrix.qza \
  --output-path achfiltered/core-metrics-results/export/bray_curtis_distance_matrix

qiime tools export \
  --input-path filtered/core-metrics-results/ach-core-metrics/bray_curtis_pcoa_results.qza \
  --output-path achfiltered/core-metrics-results/export/bray_curtis_pcoa_results


qiime tools export \
  --input-path filtered/core-metrics-results/ngn-core-metrics/bray_curtis_distance_matrix.qza \
  --output-path ngnfiltered/core-metrics-results/export/bray_curtis_distance_matrix

qiime tools export \
  --input-path filtered/core-metrics-results/ngn-core-metrics/bray_curtis_pcoa_results.qza \
  --output-path ngnfiltered/core-metrics-results/export/bray_curtis_pcoa_results



#R CODES
#Llibraries
library(phyloseq)
library(qiime2R)
library(microbiome)
library(DESeq2)
```

```
library(ggplot2)
library(randomForest)
library(rfPermute)
library(vegan)

#Names of the samples: Soil 1, Ach or ACH; Soil 2, Shd or SHD; Soil 3, Ngn or NGN; RS,
Rhizosphere, RE, root; ProHab, grouping each profits separately for RS and RE.
#Important information: Here we provided the codes with one example for phlyseq object.
Please notice that based on the aim of each specific analysis, samples are separated with
following code:
#for instance, Soil1_RS_Ac <- subset_samples(physeq, SoilType == "Ach" & Habitat ==
"Rhizosphere" & Protists %in% c("Ctrl", "Ac"))

#PERMANOVA
d <- read.delim("bray_curtis_distance-matrix.tsv",header=TRUE,row.names=1)
d <- as.dist(as(d,"matrix"))
df <- read.delim("metadata.tsv",header=TRUE,row.names=1)
df <- as(df, "data.frame")
adonis_GP1 <- adonis2(d ~ ProHab, data = df)
adonis_GP1

#PCoA
groups_GP1 <- df[["ProHab"]]
dispGP1 <- betadisper(d, groups_GP1, type=c("median"))
anova(dispGP1)

labs <- paste("PCoA", 1:4, "(", round(100*dispGP1$eig / sum(dispGP1$eig), 2), "%)")
plot(dispGP1, xlab=labs[1], ylab=labs[2], lwd=3, cex=2, col=c("red", "black", "deepskyblue",
"limegreen"))


#Phyloseq object

phy<-qza_to_phyloseq("table.qza", "rooted-tree.qza", "taxonomy.qza","metadata.tsv")

ASVs <- read_qza("table.qza")

metadata <- read.table("metadata.tsv", , sep='\t', header=T, row.names=1, comment="")
metadata <- metadata[-1,]

tree <- read_qza("rooted-tree.qza")

taxonomy <- read_qza("taxonomy.qza")
tax_table <- do.call(rbind, strsplit(as.character(taxonomy$data$Taxon), ";"))
```

```
colnames(tax_table) <- c("Kingdom","Phylum","Class","Order","Family","Genus","Species")
rownames(tax_table) <- taxonomy$data$Feature.ID

physeq <- phyloseq(
  otu_table(ASVs$data, taxa_are_rows = T),
  phy_tree(tree$data),
  tax_table(tax_table),
  sample_data(metadata))


#Venn diagram
table(meta(physeq)$Protists, useNA = "always")
physeq.rel <- microbiome::transform(physeq, "compositional")
Pro <- unique(as.character(meta(physeq.rel)$Protists))
print(Pro)
list_core <- c() # an empty object to store information
for (n in Pro){ # for each variable n in DiseaseState
    #print(paste0("Identifying Core Taxa for ", n))
    ps.sub <- subset_samples(physeq.rel, Protists == n) # Choose sample from DiseaseState by n
    core_m <- core_members(ps.sub, # ps.sub is phyloseq selected with only samples from g
                        detection = 0.001, # 0.001 in atleast 90% samples
                        prevalence = 0)
    print(paste0("No. of core taxa in ", n, " : ", length(core_m))) # print core taxa identified in each
DiseaseState.
    list_core[[n]] <- core_m # add to a list core taxa for each group.
    #print(list_core)
}


#DESeq2

diagdds = phyloseq_to_deseq2(Soil1_RS_Ac, ~ Protists)
diagdds = DESeq(diagdds, test="Wald", fitType="parametric")

res = results(diagdds, contrast=c("Protists","Ac","Ctrl"), cooksCutoff = FALSE)
alpha = 0.05
sigtab = res[which(res$padj < alpha), ]
sigtab = cbind(as(sigtab, "data.frame"), as(tax_table(physeq)[rownames(sigtab), ], "matrix"))
head(sigtab)

write.csv(sigtab, "Soil1_RS_Ac.csv")


#Random Forest
```

```
#For predictions of the shift in RE community based on the effect of protists on the RS
community

phy_test <- subset_samples(physeq, Habitat == "Root")
phy_train <- subset_samples(physeq, Habitat == "Rhizosphere")

y <- ifelse(unlist(sample_data(phy_train)[,"Protists"]) == "Ac", 1, 0)
X <- otu_table(phy_train)
X <- apply(X, 2, function(x) x+1/sum(x+1))
X <- t(log10(X))
y_test <- ifelse(unlist(sample_data(phy_test)[,"Protists"]) == "Ac", 1, 0)
X_test <- otu_table(phy_test)
X_test <- apply(X_test, 2, function(x) x+1/sum(x+1))
X_test <- t(log10(X_test))

rf <- randomForest(y = factor(y), x = X, ytest = factor(y_test), xtest = X_test, importance = TRUE,
ntree = 1000)
rf

rp <- rfPermute(y = factor(y), x = X, ytest = factor(y_test), xtest = X_test, importance = TRUE,
ntree = 1000, nrep = 1000)
rp

#The same code was used for the confirmation of DESeq results except the following
modification
Rhizo_Soil1 <- subset_samples(physeq, Habitat == "Rhizosphere" & SoilType == "Ach")

test_set <- sample(sample_names(Rhizo_Soil1), 30)
phy_train <- subset_samples(Rhizo_Soil1, !sample_names(Rhizo_Soil1) %in% test_set)
phy_test <- subset_samples(Rhizo_Soil1, sample_names(Rhizo_Soil1) %in% test_set)

#The results were obtained with the following comment
tax <- data.frame(tax_table(physeq))
imp <- importance(rp)
imp_tax <- merge(imp, tax, by = "row.names")
a <- imp_tax[rev(order(imp_tax$MeanDecreaseGini)), ]

a5 = a[which(a$MeanDecreaseGini.pval < 0.05), ]
write.csv(a5, " Rhizo_Soil1_Ac.csv")
```
